# On the conservation of white-clawed crayfish in the Iberian Peninsula: Unraveling its genetic diversity and structure, and origin

**DOI:** 10.1371/journal.pone.0292679

**Published:** 2023-10-13

**Authors:** María Martínez-Ríos, Laura Martín-Torrijos, Gloria Casabella-Herrero, Perla Tedesco, Annie Machordom, Javier Diéguez-Uribeondo

**Affiliations:** 1 Department of Mycology, Real Jardín Botánico, RJB-CSIC, Madrid, Spain; 2 Department of Veterinary Medical Sciences *Alma Mater Studiorum*, University of Bologna, Ozzano dell’Emilia, Italy; 3 Department of Biodiversity and Evolutionary Biology, Museo Nacional de Ciencias Naturales (MNCN-CSIC), Madrid, Spain; Complutense University of Madrid: Universidad Complutense de Madrid, SPAIN

## Abstract

European crayfish species are a clear example of the drastic decline that freshwater species are experiencing. In particular, the native species of the Iberian Peninsula, the white clawed-crayfish (WCC) *Austropotamobius pallipes*, is listed as “endangered” by the IUCN and included in Annex II of the EU Habitat Directive and requires especially attention. Currently, implemented conservation management strategies require a better understanding of the genetic diversity and phylogeographic patterns, as well as of its evolutionary history. For this purpose, we have generated the largest datasets of two informative ribosomal mitochondrial DNA regions, *i*.*e*., cytochrome oxidase subunit I and *16S*, from selected populations of the WCC covering its geographical distribution. These datasets allowed us to analyze in detail the (i) genetic diversity and structure of WCC populations, and (ii) divergence times for Iberian populations by testing three evolutionary scenarios with different mtDNA substitution rates (low, intermediate, and high rates). The results indicate high levels of haplotype diversity and a complex geographical structure for WCC in the Iberian Peninsula. The diversity found includes new unique haplotypes from the Iberian Peninsula and reveals that most of the WCC genetic variability is concentrated in the northern and central-eastern regions. Despite the fact that molecular dating analyses provided divergence times that were not statistically supported, the proposed scenarios were congruent with previous studies, which related the origin of these populations with paleogeographic events during the Pleistocene, which suggests an Iberian origin for these WCC. All results generated in this study, indicate that the alternative hypothesis of an introduced origin of the Iberian WCC is highly improbable. The result of this study, therefore, has allowed us to better understand of the genetic diversity, structure patterns, and evolutionary history of the WCC in the Iberian Peninsula, which is crucial for the management and conservation needs of this endangered species.

## Introduction

Freshwater ecosystems are among the most diverse and vulnerable in the world [1] while barely constitute the 1% of the Earth’s surface [2]. Freshwaters are experiencing declines in biodiversity far greater than those in the most affected terrestrial ecosystems [3] and the protection of its biodiversity requires “immediate action” [4]. Freshwater species often exhibit a high degree of endemism and high extinction rates [5], which has triggered that the populations of freshwater species have declined by an average of more than 80% in the last 50 years [6]. European crayfish species are a clear example of the drastic decline that populations of freshwater species are suffering. Their populations have experienced a rapid decline due to multiple causes such as overexploitation, water pollution, flow modification, habitat destruction, and populations fragmentation [7], but, mainly, due to the emerging crayfish plague disease caused by the pathogen *Aphanomyces astaci* and spread by American invasive crayfish species [1]. This is especially relevant since freshwater crayfish are keystone species for freshwater ecosystems [1] and play a crucial role as ecosystem engineers and in the food webs due to their relatively large body size, long life span and omnivorous feeding habits [8–10]. Thus, the disappearance of crayfish could significantly alter freshwater ecosystem processes and services, species abundance, and diversity [11], triggering changes in habitat structure and functioning [12]. Therefore, European freshwater crayfish due to their rapid decline and their key role in the ecosystem urgently require management and conservation measures based on a proper knowledge of their life history and current genetic and demographic situation.

To date, there are six native crayfish species in Europe: the narrow-clawed crayfish, (*Pontastacus leptodactylus*), the thick‐clawed crayfish (*Pontastacus pachypus*), the noble crayfish (*Astacus astacus*), the white-clawed crayfish (WCC) (*Austropotamobius pallipes*), the stone crayfish (*Austropotamobius torrentium*) [13]; and the recently described *Austropotamobius bihariensis* [14]. Most of them are included in the IUCN Red list, under categories of “endangered”, “vulnerable”, or “data deficient” but with declining trend in their populations [15]. Specifically, the WCC is one of the most threatened crayfish species and, therefore, is included: (i) in the Annex II of the Habitats Directive, which enforce EU members states to take all actions needed to keep and preserve the species and its habitats [16], (ii) under the “endangered” category in the IUCN Red list [15], (iii) under the “vulnerable” category in the Spanish Catalogue of Threatened Species [17], and (iv) as “on risk of extinction” category in the Catalogues of Threatened species by most of the Local Governments (*i*.*e*., Andalucía, Aragón, Cataluña, Extremadura, Galicia, La Rioja, Navarra and País Vasco). Thus, because of the above reasons, action and management plans have been implemented in Spain since 1996 [18–20]. For a more efficient application of these plans, a better understanding the evolutionary history, genetic diversity and structure patterns of the endangered species is of key importance [21–26]. Thus, the evolutionary history can allow us to unravel the origin of their populations and to explain their current biogeographic patterns. Furthermore, the identification of the genetic diversity of this threatened species would enable us to design programs to maintain its genetic variability and, consequently, its adaptative potential and fast evolutionary response in changing environments [1].

The WCC species is distributed across western, central, and southern Europe [13,27]. Its taxonomic status is complex since there seems to be a mito-nuclear discordance. Molecular studies based on nuclear DNA supported the existence of one species, because of the absence of variability [23,28]. However, the studies based on mitochondrial DNA (mtDNA) indicate that the WCC is a species complex with two main monophyletic lineages: (i) *A*. *pallipes*, which inhabits the northernmost area of the distribution range of the WCC, and (ii) *Austropotamobius italicus*, which is distributed from the Balkan to the Iberian Peninsula. The lineage *A*. *italicus* comprises four clades that are distributed from eastern to western Europe: *A*. *i*. *carsicus*, *A*. *i*. *meridionalis*, *A*. *i*. *carinthiacus*, and *A*. *i*. *italicus* [23,29–32].

The first genetic studies on the Iberian WCC populations showed that these belonged to the *A*. *i*. *italicus* clade [31] and suggested a scare genetic diversity [31,33]. In order to explain these results, two hypotheses were discussed on the origin of the white-clawed crayfish in the Iberian Peninsula [34]: (i) one based on an anthropogenic origin, the consequence of an introduction of crayfish from Italy to Spain, and (ii) a second based on the native nature of WCC in its westernmost distribution range. This last hypothesis was explained by several scenarios that involved a successive number of postglacial ancient and recent bottlenecks, in addition to the impact of crayfish plague. The last hypothesis was supported by further genetic studies [23,35–39]. These investigations reported a clear genetic differentiation because of higher haplotypic diversity indices than those first described, and the uniqueness of numerous haplotypes [36–39]. Moreover, this genetic diversity showed a consistent genetic structure [37–39] that recent human translocations could hardly explain. Indeed, the evolutionary history and biogeographical patterns of the WCC are in line with these results. Divergence time estimates suggested that the dispersion of *A*. *italicus* from its ancestral range and the subsequent differentiation may have taken place during the Pleistocene [23] as well as a recent split between *A*. *i*. *italicus* populations from the Iberian and Italian Peninsula that could have taken place during the early last glacial maximum, about 34,800 years ago [36]. However, the hypothesis of a recent introduction first proposed by Grandjean et al. [34], followed by Trontelj et al. [33] was later claimed by Clavero et al. [40], which add again great confusion into the matter.

The interpretations of the origin of the Iberian WCC have been based on the selected DNA regions used in these previous studies. Most genetic studies performed in crayfish have been based on mtDNA regions, specifically the *16S* rRNA (*16S*) and/or cytochrome *c* oxidase subunit one (*COI*) mtDNA genes. Although these mitochondrial genes evolve at different rates [41–43], both are useful markers for detecting intraspecific genetic variation in crustaceans [44–47] and, in particular, in the WCC [29,33,36,38,39,48]. However, a sequence analysis of a larger length of these regions has proved to be more informative and to clarify the genetic diversity in the Iberian Peninsula [37–39]. In molecular dating, node divergence times studies can also vary according to the calibrations of the nodes applied. In previous genetic crayfish studies, authors have used different substitution rates previously estimated for both *16S* and *COI* mtDNA markers, such as substitution rates for arthropods [49,50], decapod crustaceans [33,51], grapsid crabs [36,52] or snapping shrimps [36,53].

Therefore, in this study, we aimed to elucidate the genetic diversity and structure, and the origin of the WCC in the Iberian Peninsula by generating and analyzing the information from the largest *16S* and *COI* mtDNA sequences set from the whole geographical range of populations of WCC collected so far. For this purpose, we studied: (i) the genetic diversity and structure of WCC populations using two datasets with different concatenated length fragments of these mtDNA genes, and (ii) the divergence times for Iberian and Italian populations by testing three selected evolutionary scenarios with different mtDNA substitution rates (low, intermediate, and high rates). The results of this study are crucial for the management and conservation needs of this endangered species.

## Materials and methods

### Crayfish sampling

All experimental procedures, animal manipulations, and field sampling were conducted in accordance with both EU and Spanish legislation. All analyses were performed following the regulations of the Spanish Ministry of Economy (MINECO). No additional permits were necessary for the laboratory studies, as ethical approval under Spanish law is not required for working with arthropod invertebrates. Furthermore, this study was carried out in strict accordance with the recommendations and protocols established in prior research. A total of 159 specimens of WCC were collected from 35 Iberian and Italian populations (32 from the Iberian Peninsula and three from the Italian Peninsula; S1 Table) from less sampled areas in previous analyses. Sampling was carried out in collaboration with the local environmental officers following the issuance of collection permits. Crayfish were captured using nets, traps, and by hand. A walking leg was sampled from each crayfish and preserved in 100% ethanol. Crayfish were released into its location soon after the tissue was sampled. The number of crayfishes captured for each population was unequal due to the limitations of local authorities according to the population size.

### DNA extractions, amplification, and sequencing

Genomic DNA was extracted from the sampled tissue from each individual following the protocol described in Martín-Torrijos et al. [39]. Mitochondrial *16S* and *COI* genes were used as markers in this study for detecting intraspecific genetic variation in crayfish. The primers pair used to amplify the mitochondrial *16S* gene, 1472 [54] and Tor12sc [55], amplified a fragment that included partial sequences of the 12S rRNA, the *16S* and the val-tRNA. The primer pairs used to amplify the mitochondrial *COI* gene was C/N 2769 [56] and LCO1490 [57]. Both primers pairs were used in a single round PCR following the protocols in Matallanas et al. [38], including negative and positive controls. Three microliters of aliquots of the amplification products were analyzed to check positive amplicons in 1% agarose TAE gels stained with SBYR1Safe (Thermo Fisher Scientific, Waltham, MA, USA). The amplified products were purified using a QIAquick PCR Purification Kit (Qiagen, Germany). Double-stranded PCR products were sequenced using an automated sequencer (Macrogen, Spain). Sequences were assembled and edited using the program Geneious v6.14 8 [58], and a BLAST search was run on each sequence to confirm they belonged to WCC species.

### Database samples and datasets

To compare the resolution of two previously used approaches to study the genetic diversity and structure of WCC populations, we generated two datasets based on the total length of the *16S* and *COI* mtDNA regions (S1 Table): (i) dataset 1, which included the sequences from WCC populations sampled in this study and 1191 WCC samples from 202 populations download from GenBank and generated in previous studies [23,36,38,39,59]. These sequences had a length of 414 base pairs (bp) for *16S* sequences and 534 bp for *COI* sequences (concatenated length of 948 bp) and covered the whole geographical distribution range of the WCC as well as all lineages and clades previously defined for this species complex (*A*. *pallipes*, *A*. *i*. *carsicus*, *A*. *i*. *meridionalis*, *A*. *i*. *italicus*, and *A*. *i*. *carinthiacus*); and (ii) dataset 2, which included the sequences from WCC crayfish sampled in this study and 515 WCC samples corresponding to 72 populations from the Iberian Peninsula and Italy download from GenBank and generated in previous studies [36,38,39]. These sequences had a longer region length of 1298 bp for *16S* sequences and 1151 bp for *COI* sequences (concatenated length of 2449 bp).

### Genetic diversity analyses: Genetic diversity indices and haplotype networks

We used the program DNAsp v.5.10.01 [60] to estimate the following genetic diversity indices for the populations in dataset 1 and dataset 2: the number of polymorphic (segregating) sites (S), the number of haplotypes (H), the haplotype diversity (Hd, the probability that two haplotypes drawn at random from the population are not the same [61]), the nucleotide diversity (π, the average number of nucleotide differences per site between two sequences [61]), the Tajima’s D (D, the relationship between the segregating sites and the average number of nucleotide differences estimated from pairwise comparison [62]), and Fu’s Fs (Fs, statistical tests of neutrality of mutations against a class of alternative models, under which DNA polymorphisms tend to exhibit excesses of rare alleles or young mutations [63]). Additionally, we performed a rarefaction of the H for Iberian and Italian populations from *A*. *i*. *italicus* clade in order to equal the sample size in both regions and compare these results, since the number of the populations and, consequently, crayfish, was higher for the Iberian Peninsula in both datasets (S1 Table). The number of Iberian crayfish was higher than that of the Italian in both datasets. Therefore, we equaled the number of Iberian crayfish to the Italians by a random selection process. We repeat this process independently for 30 times and, then, we calculated the mean rarefied number of unique haplotypes obtained for the Iberian region (*i*.*e*., the mean expected number of Iberian haplotypes if its sample size was the same as in Italian region) and the mean rarefied number of shared haplotypes between these two regions. We statically analyzed the rarefied data with those observed applying a Pearson chi-squared test to detect differences between the number of observe and expected haplotypes. Finally, we represented the mutational changes between the sequences throughout the most parsimonious haplotype network using TCS v.1.21 [64] and the genealogical relationships were visualized using PopArt v1.7.2 [65].

### Phylogenetic analyses

To confirm the genetic identity of the sampled crayfish populations, we performed phylogenetic analyses with the different haplotypes of the mtDNA sequences of the crayfish collected here and those found in dataset 1, which included all the genetic groups defined for the WCC species (*i*.*e*., lineages and clades). We used JModelTest v.2.1.10 [66] to identify the best nucleotide substitution model for the *16S* and the *COI* mtDNA regions, under the Bayesian Information Criterion (BIC). We used the Bayesian Inference (BI) phylogenetic approximation, implemented in MrBayes v.3.2.6 [67]. Phylogenetic analyses were run individually for both genes (*i*.*e*., the *16S* the *COI* mtDNA genes), and combined (*i*.*e*., the concatenated genes). Sequences of stone crayfish (*A*. *torrentium*), noble crayfish (*A*. *astacus*), and narrow-clawed crayfish (*P*. *leptodactylus*) were used as outgroups. Nodes with posterior probabilities (pp) values greater than 0.95 in BI were considered supported. Resulting phylogenetic trees were visualized using FigTree v.1.4.3 [68].

### Genetic structure analyses

We examined the WCC genetic structure in both datasets with a spatial analysis of the molecular variance of the populations using SAMOVA v2.0 [69]. This tool defines K groups of genetically homogeneous populations, maximizing the differentiation from each other and identifying genetic barriers between these groups. The aim is to maximize the proportion of total genetic variance due to differences between groups of populations (K). We run SAMOVA v2.0 from K = 2 to K = 20 (the maximum K that the programme allows to compute) for each dataset with 1,000 simulated annealing processes per run, using the concatenated matrix of mtDNA genes. The best genetic structure was determined by the highest statistically significant value of F_CT_ (*i*.*e*., fixation index between groups of populations) for a given K.

### Divergence times

We used BEAST v1.8.4 (Bayesian evolutionary analysis by sampling trees) [70], to estimate the timing of divergence between Iberian and Italian populations of the WCC, using the *16S* and *COI* mtDNA sequences from dataset 1. We used previously reported divergence rates for each mitochondrial gene as method of calibration of nodes. We selected different substitution rate values for each mtDNA marker, creating three divergence scenarios: (i) for the first scenario (hereafter, high rates scenario), we selected the highest substitution rate reported and used for each mitochondrial gene in previous studies, *i*.*e*., a substitution rate of 0.012 per site per million years for *16S* gene [71] and 0.026 for *COI* gene [36,53]; (ii) for the second scenario (hereafter, low rates scenario), we selected the lowest substitution rate reported and used for each mitochondrial gene in previous studies, *i*.*e*., a substitution rate of 0.008 per site per million years for *16S* gene [72] and 0.0115 for *COI* gene [49,50]; (iii) for the third scenario (hereafter, intermediate rates scenario), we selected an intermediate substitution rate for each gene, *i*.*e*., a substitution rate of 0.010 per site per million years for *16S* gene [72] and 0.014 for *COI* gene [36,52,73]. The “Nucleotide substitution model” prior was set to HKY + G for both *16S* and *COI* markers for all the scenarios. The “Clock model” prior was set as uncorrelated relaxed clock with a lognormal distribution [74,75]. The “Tree” prior used was the birth-death process model for speciation [76,77].

Each BEAST analysis was run with independently with 100,000,000 generations and parameters were sampled every 10,000 generations. Trace files were checked in TRACER [68] for convergence between MCMC chains and to ensure that all effective sample size (ESS) values of the parameters were >200 after elimination of the 20% sampled trees from each run as burn-in. Resulting phylogenetic trees were visualized using FigTree v.1.4.3 (version 1.4.3; http://tree.bio.ed.as.uk/software/figtree/) with the mean and 95% highest posterior density (HPD) interval for each node age.

## Results

### DNA extractions, amplification, and sequencing

We obtained sequences for the two mtDNA regions for all the 159 specimens of the 35 populations of WCC from the Iberian Peninsula and Italy collected in this study (S1 Table). All sequences showed high quality. However, the *COI* sequences for the GIR5 population showed double peaks in 16 positions. These double peaks were only considered when the electropherograms were well-defined and there was a clear difference from the baseline (S1 Fig). The two reads (forward and reverse) were not always identical in height of the double peaks due to the PCR yield may vary depending on the hybridization of the primers and the polymerase activity. Since we have never obtained these types of results in previous analyses for the same mtDNA regions in crayfish, we repeated the DNA extractions, amplifications, and sequencing. As a results, we found again these ambiguous sites at the same positions in the same GIR5 crayfishes (S2 Table). This allowed us to verify that the presence double peaks obtained in the *COI* region are not due to errors in the PCRs, sequencing, or contaminations. Therefore, we decided to exclude all sequences of the crayfishes from the GIR5 population from the following analyses except from the phylogenetic analyses to check the genetic identity of these crayfishes.

### Database samples and datasets

The dataset 1 included a total of 1344 WCC samples from 249 populations (34 populations from this study and 202 more obtained from GenBank), with a length of 414 base pairs (bp) for *16S* sequences and 534 bp for *COI* sequences, *i*.*e*., a concatenated length of 948 bp. This dataset covered the worldwide geographical distribution range and all lineages and clades previously defined to the WCC species complex (*A*. *pallipes*, *A*. *i*. *carsicus*, *A*. *i*. *meridionalis*, *A*. *i*. *italicus*, and *A*. *i*. *carinthiacus*). The dataset 2 included a total of 668 WCC samples from 106 populations (34 populations from this study and 72 more obtained from GenBank), with a longer region length of 1298 bp for *16S* sequences and 1151 bp for *COI* sequences, *i*.*e*., a concatenated length of 2449 bp. These populations were from the Iberian and Italian peninsulas.

### Genetic diversity indices and haplotype networks

We estimated the genetic diversity indices for the populations in each dataset. The WCC populations from dataset 1 showed 132 polymorphic sites and hosted 92 haplotypes. This dataset had a high mean haplotype diversity (Hd = 0.735). Populations CRO3, CRO6, GRA9, IT6, BOS6, BOS10, CRO17, CRO18, CRO19, CRO20, SO3, TE1 and TE7 had the highest haplotype diversity (Hd = 1), but for nine of them only two specimens were analyzed (S3 Table). The 18% of the Iberian populations showed high values of haplotype diversity (1≥Hd≥0.5), the 12% had low values (0.5>Hd>0), and 70% did not show haplotype diversity (Hd = 0). The 20% of the Italian population showed high values of haplotype diversity (1≥Hd≥0.5), the 3.3% had low values (0.5>Hd>0), and 76.7% did not show haplotype diversity (Hd = 0) (S3 Table). The nucleotide diversity was low for all European populations (0≤π≤0.018) (S3 Table). Tajima´s D obtained negative values for 21 populations, indicating fewer haplotypes than the number of S, while this value was positive for 26 population, indicating more haplotypes than S. The other populations showed Tajima´s D values were not statistically significant. Fu´s Fs value was negative for 18 populations, evidence of an excess of alleles, and was positive for 36 populations, evidence of a deficiency of alleles. The other populations showed Fu´s Fs values that were not statistically significant (n/c; S3 Table). There were 14 of the 154 Iberian populations and five of the 30 Italian populations with positive values for Tajima´s D and Fu´s Fs indices, while only four Iberian populations had negative values for both indices. The populations included in the dataset 2 showed 53 polymorphic sites and hosted 49 haplotypes. This dataset showed also a high mean haplotype diversity (Hd = 0.744). The populations with the highest haplotype diversity (Hd = 1) were BU82, CU1, CU3, CU4, HU6, NA7, SO3, TE1, TE7 and ZA2. The 53% of the Iberian populations showed high values of haplotype diversity (0.5≤Hd≤1), the 19% had low values (0.18<Hd<0,5), and 43% did not show haplotype diversity (Hd = 0). Two of the four Italian population showed high values of haplotype diversity (0.5≤Hd≤1), while the other two did not show haplotype diversity (Hd = 0) (S4 Table). The nucleotide diversity was low for all European populations (0≤π≤0.007) (S4 Table). Tajima´s D obtained negative values for 24 populations, and was positive for 35. Fu´s Fs value was negative for 11 populations and positive for 65 populations (S4 Table). There were 18 of the 106 Iberian populations and two of the four Italian populations with positive values for Tajima´s D and Fu´s Fs indices, while only eight Iberian populations had negative values for both indices.

**Fig 1:**
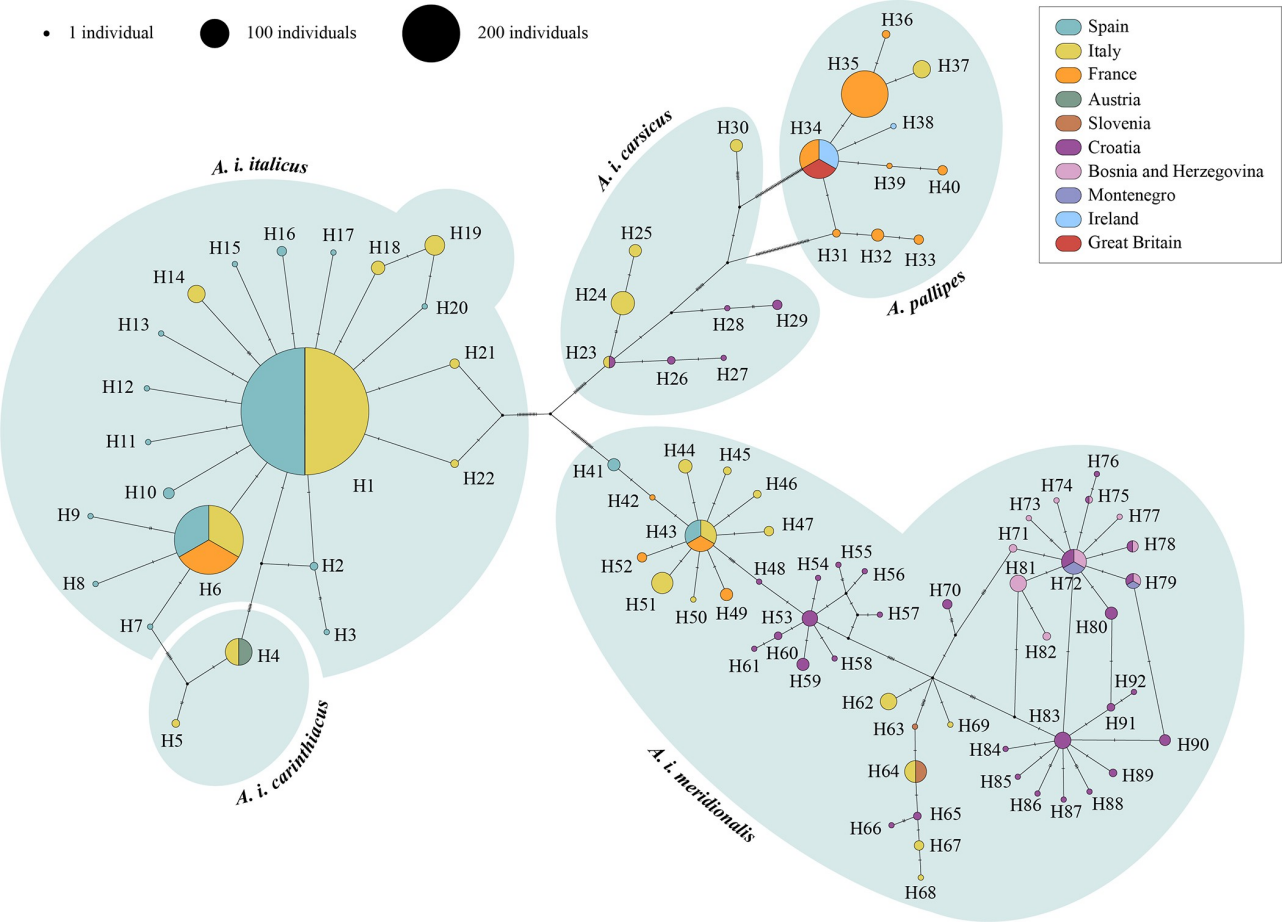
White clawed crayfish dataset 1 haplotype network. Haplotype network of white-clawed crayfish populations from dataset 1 (sequences from the whole geographical distribution range, covering all lineages and clades previously defined to the WCC species complex) based on 948 bp of the concatenated mitochondrial *16S* rRNA and cytochrome oxidase subunit I regions. Haplotypes are organized into the lineages and clades based on the mtDNA previously described to this crayfish species: *Austropotamobius pallipes*, *Austropotamobius italicus carsicus*, *A*. *i*. *meridionalis*, *A*. *i*. *italicus*, and *A*. *i*. *carinthiacus* (bluish shadows). The haplotypes are colored by the countries in which they were found. Each chart of the legend represents a different European country.

**Fig 2:**
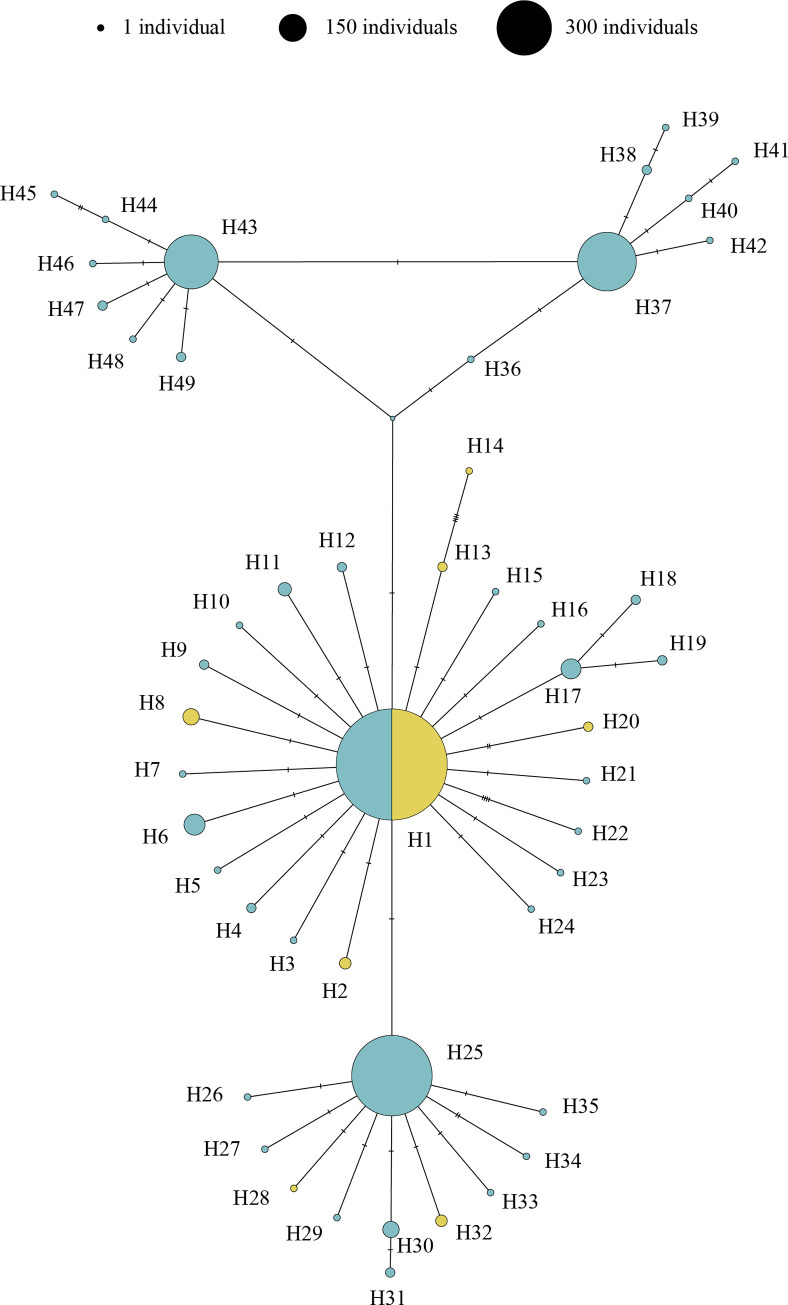
White clawed crayfish dataset 2 haplotype network. Haplotype network of white-clawed crayfish populations from dataset 2 (sequences from the Iberian and Italian peninsulas) based on 2449 bp of the concatenated mitochondrial *16S* rRNA and cytochrome oxidase subunit I regions. All the belongs to the *Austropotamobius italicus italicus* clade. The turquoise haplotypes are from Iberian Peninsula, while Italian haplotypes are represented in yellow.

We represented the mutational changes between sequences from each dataset in independent haplotype networks. The most parsimonious haplotype network for sequences from dataset 1 identified a total of 92 haplotypes (Fig 1). These haplotypes can be clustered into five genetic groups (*i*.*e*., lineages and clades) corresponding to those previously defined for the WCC species: *A*. *pallipes*, *A*. *i*. *carsicus*, *A*. *i*. *meridionalis*, *A*. *i*. *italicus*, and *A*. *i*. *carinthiacus*. Most of the specimens clustered into one of the following four haplotypes: H1 (653 specimens), H6 (201 specimens), H34 (50 specimens), and H35 (72 specimens) in dataset 1 (S3 Table). The *A*. *i*. *meridionalis* clade included the highest number of haplotypes (52 of the 92 found haplotypes in this dataset), followed by *A*. *i*. *italicus* clade (20 haplotypes), *A*. *pallipes* lineage (11 haplotypes), *A*. *i*. *carsicus* clade (seven haplotypes) and, finally, *A*. *i*. *carinthiacus* clade (two haplotypes). All the Iberian populations were clustered with the *A*. *i*. *italicus* clade, except the GIR10 population, which belonged to the *A*. *i*. *meridionalis* clade. Of the 20 haplotypes belonging to *A*. *i*. *italicus* clade, 13 were unique from Iberian populations (H2, H3, H7-H13, H15-H17 and H20 for 870 crayfish), five were unique from Italian populations (H14, H18, H19, H21 and H22 for 172 crayfish), and only two haplotypes were shared, one with Italian populations (H1) and the other with Italian and French populations (H6) (Table 1, S3 Table). These unique haplotypes from the Iberian Peninsula corresponded to the following 15 populations: BU4, BU53, BU98, BU99, CU7, CU9, GIR1, GRA2, GRA9, JA7, MA4, TE1, TE5, TE7, and ZA4 (S3 Table). The most parsimonious haplotype network for sequences from dataset 2 identified 49 haplotypes (Fig 2) of the *A*. *i*. *italicus* clade: one of them (H1) shared by Iberian and Italian populations, seven of them unique from Italian populations (H2, H8, H13, H14, H20, H28 and H32) and, the other 41 of them unique from Iberian populations (Fig 2, Table 1). These unique haplotypes from the Iberian Peninsula were distributed in most of the Iberian populations (S4 Table). Most of the specimens showed one of the four most frequent haplotypes from dataset 2: H1 (287 specimens), H25 (147 specimens), H37 (78 specimens) and H43 (66 specimens) (S4 Table).

**Table 1:** Populations, individuals and haplotype comparison of datasets 1 and 2. Comparison of unique and shared haplotypes between Iberian and Italian populations of the *A*. *i*. *italicus* clade obtained for the raw and rarefied data in the datasets analyzed, *i*.*e*., dataset 1 (a concatenated mtDNA with a length of 948 bp from 249 populations distributed over the worldwide geographical distribution range of the white-clawed crayfish) and dataset 2 (concatenated mtDNA with length of 2449 bp from 106 populations from the Iberian Peninsula and 4 populations from the Italian Peninsula). (n) indicates sample size.

		Dataset 1 (948 bp)	Dataset 2 (2449 bp)
***A*. *i*. *italicus* populations**	n	184	106
***A*. *i*. *italicus* haplotypes**	n	20	49
**Italian Peninsula**	populations	30	4
	individuals	172	27
**Iberian Peninsula**	populations	154	102
	individuals	870	647
**Italian unique haplotypes**	n	5	7
**Iberian unique haplotypes**	raw data n	13	41
	rarefied data n	5.6	5.8
**Shared haplotypes**	raw data n	2	1
	rarefied data n	2	1

We observed a difference in the number of new haplotypes reported in each dataset. The 31 populations from the Iberian Peninsula incorporated in this study (S1 Table) have added only 2 new haplotypes of *A*. *i*. *italicus* clade in dataset 1 (S3 Table, Fig 1), while they represented 14 new haplotypes when were analyzed in dataset 2 (S4 Table, Fig 2). Moreover, comparing the unique haplotypes from the Iberian populations in both datasets we found that the 870 Iberian crayfish from the dataset 1 (948 bp) were distributed in 13 haplotypes, while the 647 crayfish from the dataset 2 (2449 bp) were distributed in 41 haplotypes (Table 1). Regarding the rarefaction data that we performed to remove a potential effect of the sample size in the number of unique haplotypes for Iberian and Italian populations of the *A*. *i*. *italicus* clade between both regions, we randomly selected 172 Iberian crayfish, with their respective haplotypes, for the dataset 1 and 27 Iberian crayfish for the dataset 2. We calculated the mean rarefied number of haplotypes for the Iberian Peninsula after repeating the 30 random selective process and we obtained 5.6 and 5.8 unique haplotypes for dataset 1 and dataset 2, respectively (Table 1). The p-value for the Pearson chi-squared test was 1 (X-squared = 1.6975·10^−30^, df = 1), *i*.*e*., there were no statistical differences between the number of unique haplotypes in the Iberian and Italian regions. The rarefied number of shared haplotypes between these regions was the same as those of raw data; two shared haplotypes in dataset 1 and one shared haplotype in dataset 2 (Table 1).

### Phylogenetic analyses

The phylogenetic analyses showed that the independent analyses of the two mtDNA regions provided congruent topologies. However, the crayfishes of the GIR5 population were located at different positions depending on the mitochondrial region used in each phylogeny. Thus, the phylogeny based on the *16S* region, show that all sequences of these crayfishes from the GIR5 population clustered with haplotypes corresponding to the *A*. *i*. *meridionalis* clade (S2 Fig) except one (*i*.*e*., GIR5_3) that clustered within *A*. *i*. *italicus* clade. On the other hand, the analysis of the *COI* region showed that all these crayfishes clustered within the *A*. *i*. *italicus* clade, except one (*i*.*e*., GIR5_6), which clustered along with the *A*. *pallipes* clade (S3 Fig). Due to these mtDNA incongruences, we decided to exclude all sequences of the crayfishes from the GIR5 population from the genetic diversity and structure analyses and from the molecular dating. Regarding the other analyzed 34 populations from the Iberian and Italian peninsulas, all the sequences of their crayfish clustered with crayfish samples belonging to the *A*. *i*. *italicus* clade, as also shown below in the phylogenies obtained for the divergence time estimates (see Divergence times´ section). These sequences correspond to haplotypes H1, H6, H7, H11, H17, H18, H21, and H22, defined by dataset 1 (Fig 1).

### Genetic structure analyses

The SAMOVA analyses results are shown in Table 2. For dataset 1, the analysis defined 20 groups for the 249 population of WCC as the optimal genetic structure (see S5 Table). The G_d1_1 group clustered the majority of the populations (175 populations). The maximum F_CT_ value (F_CT_ = 0.96897) was obtained for K_d1_ = 20, maximizing the genetic variance between groups (96.9% of variance, P < 0.0001). The genetic variation among populations within groups and within populations were low (1.28% and 1.82%, respectively). For dataset 2, the analysis clustered the 107 populations into 18 groups as the optimal genetic structure (see S6 Table). The main groups, *i*.*e*., those which contained a greater number of populations, where G_d2_1 (62 populations), G_d2_4 (11 populations), G_d2_5 (5 populations) and G_d2_12 (7 populations), while the other groups were formed by one or two populations. The maximum F_CT_ value (F_CT_ = 0.72861) was obtained for K_d2_ = 18, *i*.*e*., when the percentage of genetic variation among groups was the maximum (72.86% of variation, P < 0.0001). The genetic variation among populations within groups was low (5.08%) while we obtained a slight genetic variation within populations (22.06%).

**Table 2:** SAMOVA. Spatial Analysis of Molecular Variance (SAMOVA) for dataset 1 and 2. Values for degrees of freedom (df), Sum of squares, Variance Components, percentage of variation (% Variation), Fixation indices (Fix. Indices) and p-Values are provided for each source of variation.

Source of variation		df	Sum of squares	Variance Components	% Variation	Fix. Indices	*p*-Values
**Dataset 1 SAMOVA groups (K = 20)**	Among groups	19	13574.902	18.83679	96.90	F_CT_ = 0.96897	<0.001
	Among populations within groups	216	371.473	0.24979	1.28	F_SC_ = 0.41403	<0.001
	Within populations	1108	391.708	0.35353	1.82	F_ST_ = 0.98181	<0.001
	Total	1343	14338.083	19.44010	-	-	-
**Dataset 2 SAMOVA groups (K = = 18)**	Among groups	17	347.953	0.85110	72.86	F_CT_ = 0.72861	<0.001
	Among populations within groups	88	55.646	0.05933	5.08	F_SC_ = 0.18715	<0.001
	Within populations	562	144.823	0.25769	22.06	F_ST_ = 0.77940	<0.001
	Total	667	548.422	1.16813	-	-	-

**Fig 3:**
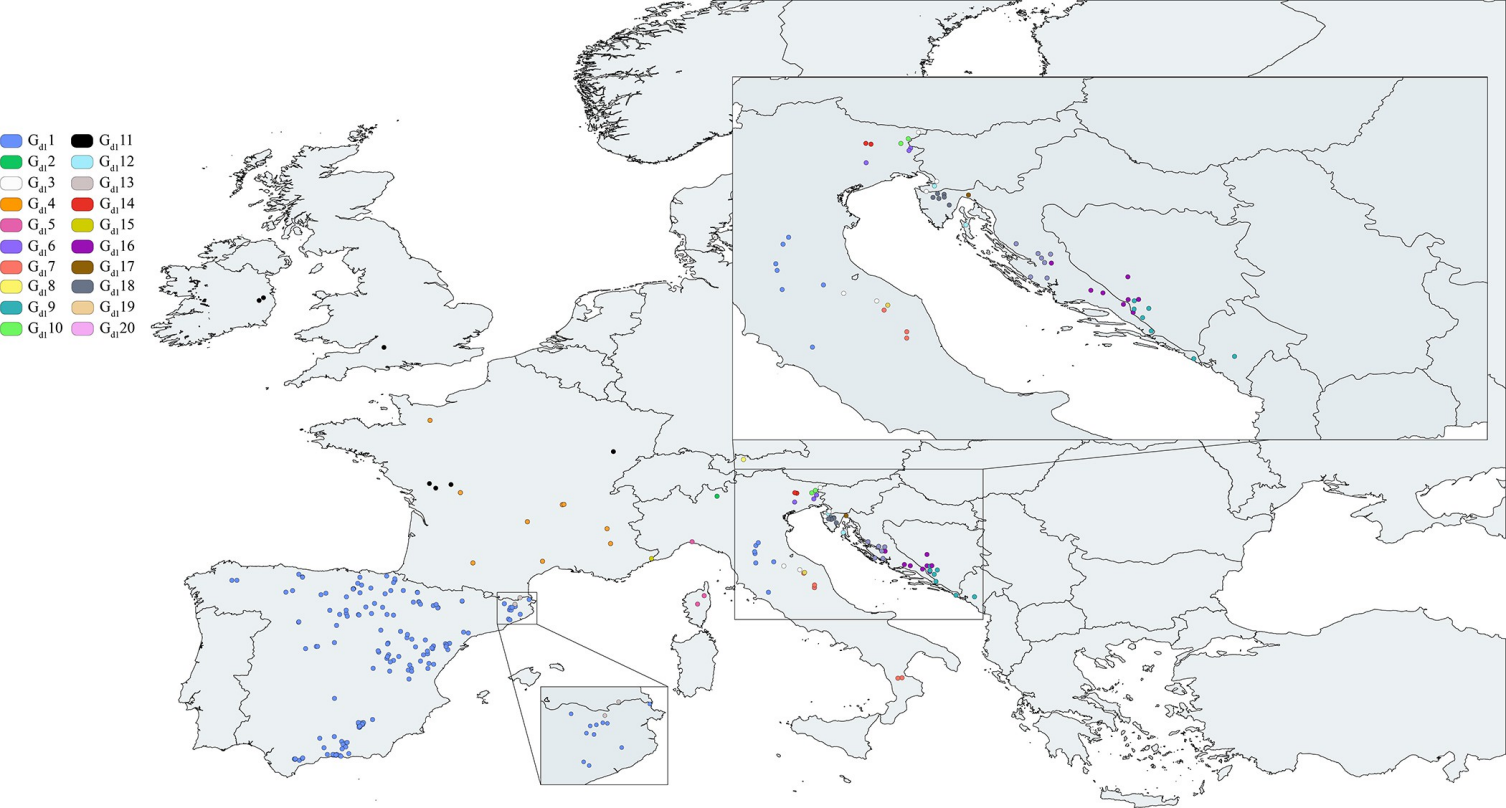
White clawed crayfish dataset 1 optimum genetic structure. Genetic structure of the white-clawed crayfish of populations from dataset 1 (sequences from the whole geographical distribution range, covering all lineages and clades previously defined to the WCC species complex) based on 948 bp of the concatenated mitochondrial 16S rRNA and cytochrome oxidase subunit I regions. European populations were clustered into 20 genetic groups (G_d1_1-G_d1_20) (S5 Table). Each color of the legend represents a different genetic group. Basemap datasets from Natural Earth [69].

**Fig 4:**
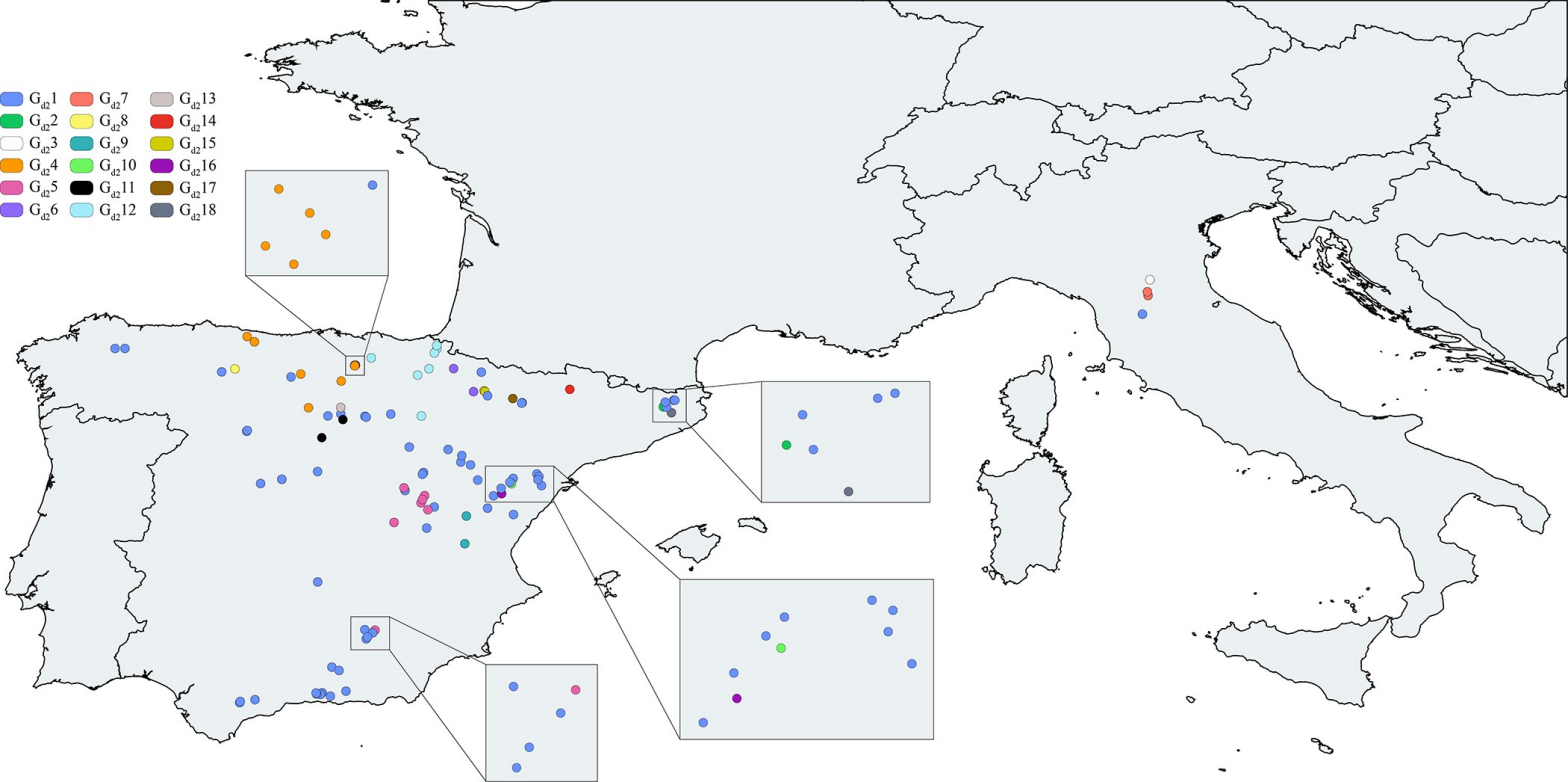
White clawed crayfish dataset 2 optimum genetic structure. Genetic structure of the white-clawed crayfish of populations from dataset 2 (sequences from the Iberian and Italian peninsulas) based on 2449 bp of the concatenated mitochondrial 16S rRNA and cytochrome oxidase subunit I regions. Iberian and Italian populations were clustered into 18 genetic groups (G_d2_1-G_d2_18) (S6 Table). Each color of the legend represents a different genetic group. Basemap datasets from Natural Earth [69].

The distributions of the population groups are shown in Figs 3 and 4. These genetic structure results showed that the Iberian populations had their diversity distributed in 15 of the 18 genetically differentiated groups in dataset 2 (2449 bp), sharing only one genetic group (G_d2_1) with Italian populations (Fig 4, S6 Table). However, this Iberian structure did not appear in dataset 1 (948 bp), where all the Iberian populations belonged to a unique genetic group shared with some Italian populations, as shown in Fig 3.

### Divergence times

**Fig 5:**
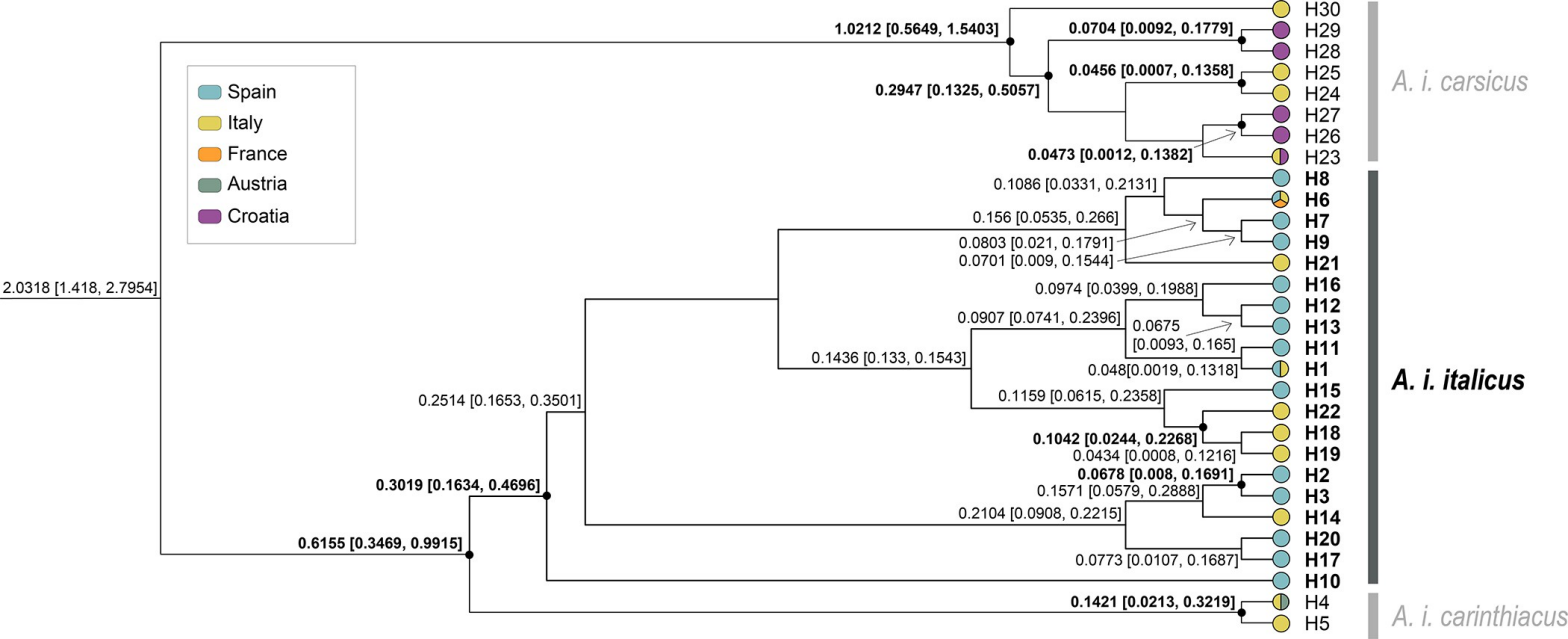
Bayesian phylogenetic tree based on low rates scenario from dataset 1. Bayesian inference phylogenetic tree with the divergence time estimates for haplotypes of the *Austropotamobius italicus italicus* clade based on the low rates scenario from dataset 1 (sequences from the whole geographical distribution range, covering all lineages and clades previously defined to the WCC species complex) based on 948 bp of the concatenated mitochondrial 16S rRNA and cytochrome oxidase subunit I regions. Node branches show the estimated mean and range of the divergence time in Mya. The nodes support with high posterior probability values (pp≥0.95) show a black circle, and the divergence times are in bold (complete tree in S4 Fig). The haplotypes are colored by the countries in which they were found. Each color of the legend represents a different European country.

**Fig 6:**
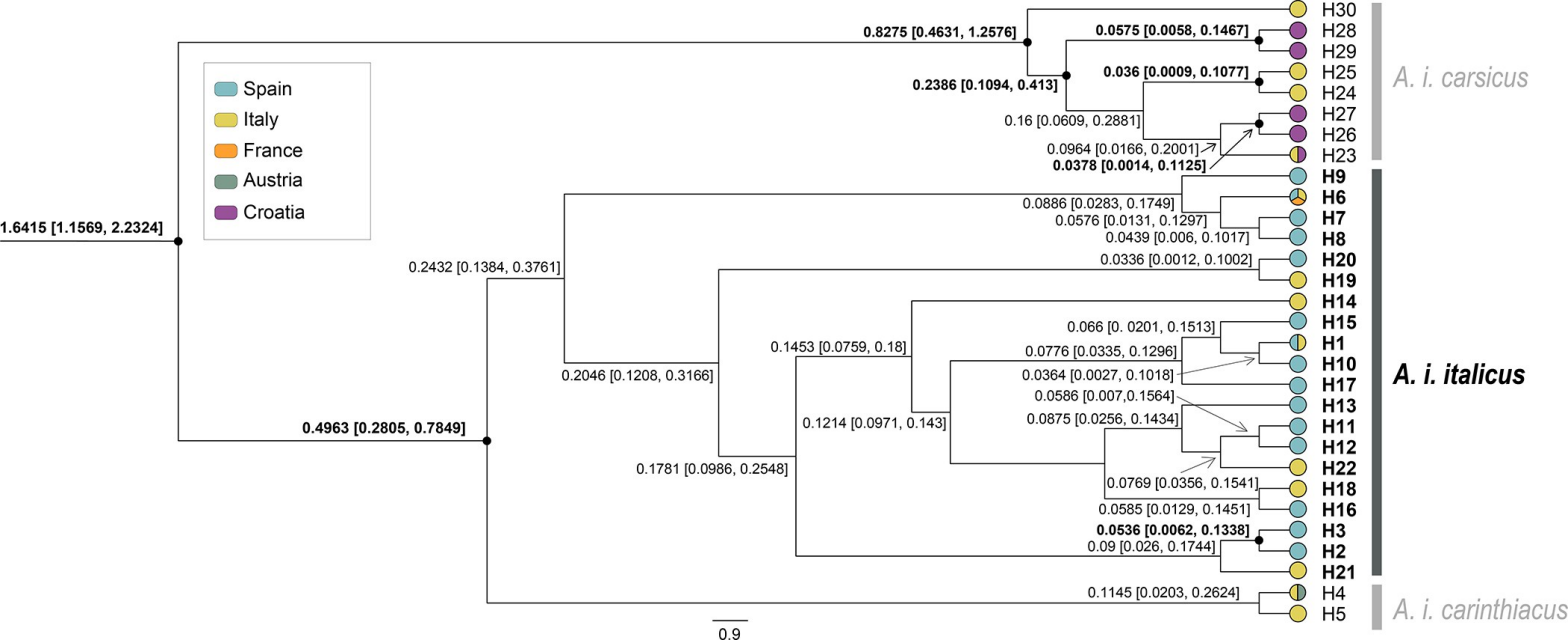
Bayesian phylogenetic tree based on intermediate rates scenario from dataset 1. Bayesian inference phylogenetic tree with the divergence time estimates for haplotypes of the *Austropotamobius italicus italicus* clade based on the intermediate rates scenario from dataset 1 (sequences from the whole geographical distribution range, covering all lineages and clades previously defined to the WCC species complex) based on 948 bp of the concatenated mitochondrial 16S rRNA and cytochrome oxidase subunit I regions. Node branches show the estimated mean and range of the divergence time in Mya. The nodes support with high posterior probability values (pp≥0.95) show a black circle, and the divergence times are in bold (complete tree in S5 Fig). The haplotypes are colored by the countries in which they were found. Each color of the legend represents a different European country.

**Fig 7:**
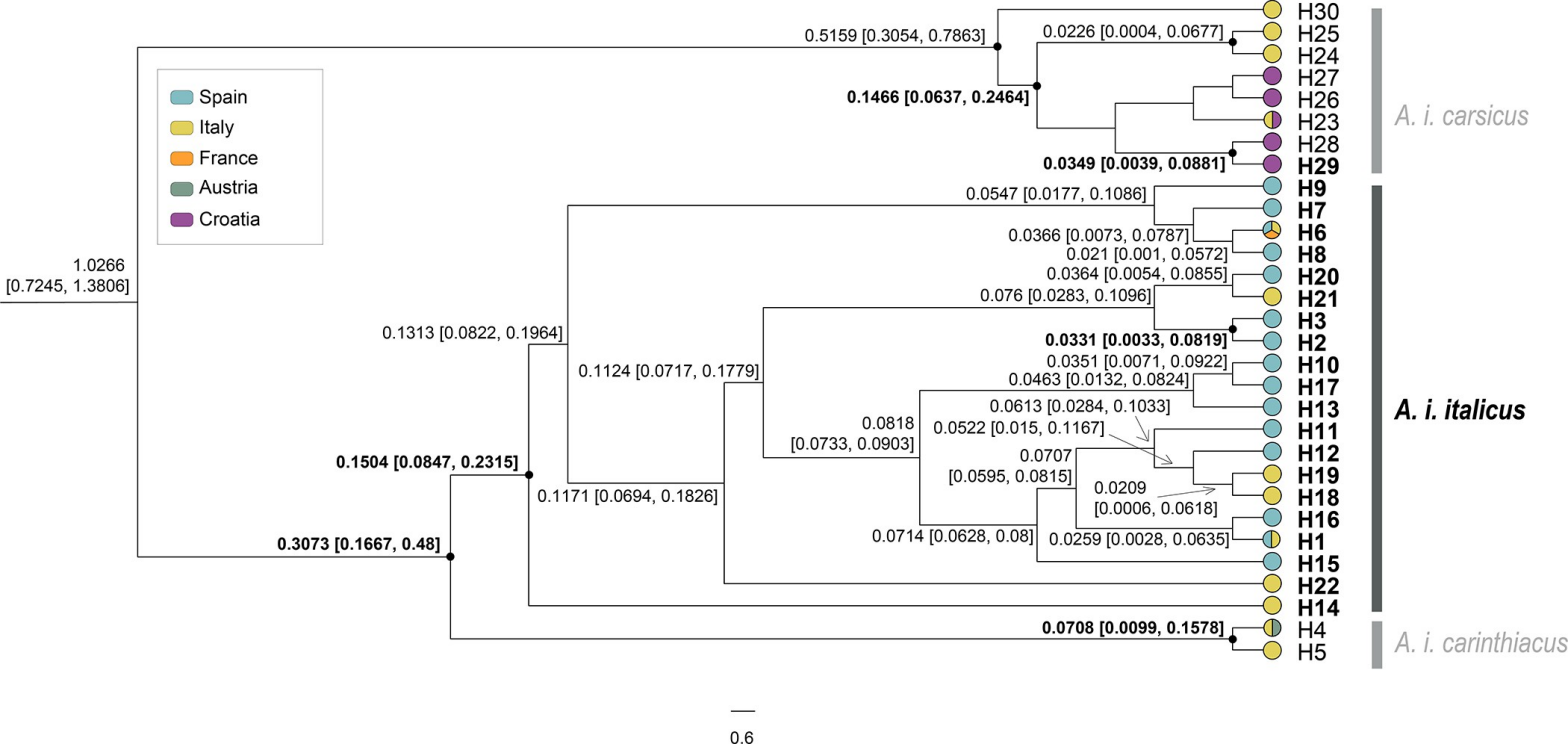
Bayesian phylogenetic tree based on high rates scenario from dataset 1. Bayesian inference phylogenetic tree with the divergence time estimates for haplotypes of the *Austropotamobius italicus italicus* clade based on the high rates scenario from dataset 1 (sequences from the whole geographical distribution range, covering all lineages and clades previously defined to the WCC species complex) based on 948 bp of the concatenated mitochondrial 16S rRNA and cytochrome oxidase subunit I regions. Node branches show the estimated mean and range of the divergence time in Mya. The nodes support with high posterior probability values (pp≥0.95) show a black circle, and the divergence times are in bold (complete tree in S6 Fig). The haplotypes are colored by the countries in which they were found. Each color of the legend represents a different European country.

The phylogenetic trees obtained from the three scenarios showed similar topologies. Divergence time estimates between the *Astacus* and *Austropotamobius*, between *Austropotamobius* spp. and within WCC obtained in the different scenarios varied in the mean values, but their 95% HPD overlapped (S4, S5 and S6 Figs). The four monophyletic clades within the *A*. *italicus* lineage were highly supported by posterior probability values (pp≥0.95) in all the scenarios. One of these clades, *i*.*e*., *A*. *i*. *italicus*, comprised the majority of the samples from the Iberian Peninsula. This clade was dated to HPD 0.1634–0.4696 Mya (mean value of 0.3019 Mya) for the low rates scenario (Fig 5); HPD 0.1384–0.3781 Mya (mean value of 0.2432 Mya) for the intermediate rates scenario (Fig 6); and, HPD 0.0847–0.2315Mya (mean value of 0.1504 Mya) for the high rates scenario (Fig 7).

Within *A*. *i*. *italicus*, all scenarios showed several divergence events between the Iberian and Italian populations, and some of their haplotypes related differently. Only the split node between populations belonging the Iberian haplotypes H2 and H3 was estimated with high support (pp ≥0.95) in the three scenarios. For the low rates scenario (Fig 5), the mean value in this node was 0.0678 Mya (HPD 0.008–0.1691 Mya); for the intermediate rates (Fig 6) was 0.0536 Mya (HPD 0.0062–0.1338 Mya); and for the high rates (Fig 7) was 0.0331 Mya (HPD 0.0033–0.0819 Mya). The divergence time for the split node between the Italian haplotypes H22 and H18+H19 in the low rates scenario (Fig 5) was also highly supported; the mean value was 0.1042 Mya, with HPD 0.0244–0.2268 Mya. All other divergence estimates had split nodes with a low posterior probability (pp<0.95), including all those between the Iberian and Italian population haplotypes. Thus, in the low rates scenario (Fig 5), the split nodes between the Iberian and Italian haplotypes placed the origin of Iberian populations between a maximum and a minimum of 0.0579–0.2888 Mya (pp<0.95); between 0.007–0.1744 Mya (pp<0.95) in the intermediate rates scenario (Fig 6); and around 0.015–0.1167 Mya (pp<0.95) in the high rates scenario (Fig 7).

## Discussion

The conservation of the endangered WCC in the Iberian Peninsula requires further assess its genetic diversity and structure, and, due to recent speculations regarding an anthropogenic origin, a better understanding of its evolutionary origin. In this work, we have compiled all available data for the WCC from two mtDNA regions, *i*.*e*., *COI* and *16S*, and generated the largest sequence dataset for these regions for this species. This has allowed us to update and compare previous investigations regarding the genetic diversity and structure of the WCC in the Iberian Peninsula as well as to analyze several evolutionary scenarios.

The haplotype diversity (Hd) of the Iberian and Italian populations showed similar levels in both analyzed datasets. These results showed that there are populations with high levels of haplotype diversity while others only contained a single haplotype. This finding contrasts with the hypothesis of an introduction from Italy, since in this case, we would expect a lower haplotype diversity in the Iberian populations than that of the Italians, or any diversity in the Iberians. The lack of diversity in some Iberian populations can be explained by different reasons, such as the recent intense translocations after the strike of the crayfish plague [19]. However, the high levels of haplotype diversity do not match with the hypothesis of an anthropogenic introduction. Previous studies that compared introduced and native populations in other species demonstrated the absence of haplotype diversity in populations originated from an introduction event, while the native range of a species generally showed a high genetic diversity [78,79].

Moreover, we found that the nucleotide diversity in WCC of the Iberian and Italian populations is low. Previous studies reported nucleotide diversity values for several European regions (including Italian and Iberian peninsulas) similar to those obtained in this study [33]. Some authors have interpreted the lack of genetic diversity of the Iberian populations as indicative of an introduction [29,31,33,80]. However, in both datasets analyzed, we also encountered a low nucleotide diversity for all populations of WCC in Europe. This general low nucleotide diversity could be due to a strong reduction in population size caused by the pathogen *A*. *astaci* responsible for the devastation of up to 80% of the European crayfish populations [1] and other reasons, such as habitat alterations, drought occurrences, water contamination, and the influence of climate change [19]. Therefore, the low nucleotide diversity of the Iberian WCC does not necessarily have to be the consequence of an introduction from Italy.

The Tajima´s D and Fu´s Fs indices indicated that some Iberian and Italian populations (positive values) have suffered a sudden population contraction as it would be expected from a recent population bottleneck, and other Iberian populations (negative values) are experiencing a population expansion. The results of these indices seem to show the dynamics for all European crayfish species as a result of the impact of the crayfish plague [1,81], and is in accordance to the hypothesis of Grandjean et al. [31], that pointed out that this disease caused an abrupt decline of the WCC since the 70´s. Therefore, this decline may have led to the loss of unique and/or shared haplotypes in both the Iberian and Italian peninsulas over the last decades. This makes it difficult to unravel the evolutionary relationships between their populations. Some authors have justified the existence of the unique haplotypes in the Iberian Peninsula as the result of their disappearance in Italy due to historical declines and associated genetic erosion [40]. However, it is quite unlikely that a biased loss of Italian haplotypes had happened since both Italian and Iberian populations have suffered intense bottlenecks in recent history. In fact, a larger loss of haplotype diversity was more likely to occur in the Iberian Peninsula since this has been devastated by the three most virulent haplotypes of the crayfish plague pathogen (*i*.*e*., b, d1 and d2-haplotypes) [82–85]. However, the Italian populations were mainly affected by a single haplotype that has low virulence (*i*.*e*., a-haplotype) [86,87]. Thus, the intense translocations carried out from crayfish plague free-populations in the Iberian Peninsula to conserve the WCC [19] can explain the results showing WCC populations exhibiting an expansion trend.

Furthermore, we found that the majority of haplotypes of *A*. *i*. *italicus* are unique to the Iberian or Italian populations, while the number of shared haplotypes between the populations from both peninsulas is low in both datasets. This contrasts again to what we would be expected in a scenario of an introduced population from Italy to the Iberian Peninsula. High haplotypic diversity as well as unique haplotypes have been interpreted as support for the native origin of crayfish in the Iberian Peninsula [23,36,38,39]. In the analysis of dataset 1, which includes populations from the whole distribution range of the WCC, we found two shared haplotypes with Italian populations, *i*.*e*., the most common haplotype (H1), and another haplotype shared also with France (H6). This is compatible with a previously theory of a Circum-Mediterranean distribution of *A*. *i*. *italicus* before the Last Glacial Period (LGP) in Pleistocene [35,36,88]. This distribution is also found in other freshwater species [89,90] associated with the isolation in glacial refugia during the Last Maximum Glacial. Some authors suggested that the unique haplotypes found in an introduced population can be missing from their native area due to poor sampling [40,91,92]. However, the analysis of rarefied data (Table 1) does not support the claim that an increase on sampling effort Italian Peninsula will result on a higher number of unique haplotypes than that of the Iberian populations. In addition, we should take in consideration that a larger mtDNA region size (*i*.*e*., the size used in the dataset 2) allows to detect a higher variability than that of the short region size (*i*.*e*., the size used in dataset 1) commonly used in previous studies [23,36,38,39,59].

The populations of WCC in the Iberian Peninsula were geographically structured as judged from analyses of dataset 2 (*i*.*e*., the Iberian and Italian populations) and, therefore, support previous studies indicating an origin in the Iberian Peninsula [38,39]. The fact that some other studies [80] were unable to show a genetic structure has been used previously as an indication of the introduced status of other *Austropotamobius* populations. However, the lack of the genetic structure shown in dataset 1 (*i*.*e*., populations from whole distribution range) seems to be mainly a computational artefact since the SAMOVA analysis does not allow to create more than 20 population groups. We speculate that Iberian populations from dataset 1 would show a biogeographic structure if there were no limits to the number of potential population groups.

The divergence time estimates suggested a separation of the Iberian and Italian populations of the WCC several thousands of years ago during the Pleistocene for all the proposed scenarios. Even though the results of molecular dating were not fully conclusive, since split nodes between Iberian and Italian populations were not supported by posterior probability values, the estimated values in the Pleistocene are in accordance to the results of previous research performed by Pedraza et al. [36] and Jelic et al. [23]. The ranges of divergence times obtained for the genus *Astacus* and *Austropotamobius*, and between *Austropotamobius* species, and, even, within WCC largely overlapped with those obtained by these previous studies [23,36]. In addition, the monophyletic clades within the *A*. *italicus* lineage were highly supported by posterior probability values. Under all proposed evolutionary scenarios, the Iberian and Italian populations seem to have diverged in several events during the Pleistocene. These results would be in line with the theory proposed about a unique distribution range for the *A*. *i*. *italicus* clade populations from the Iberian Peninsula eastwards until at least the Central Apennines (*i*.*e*., Circum-Mediterranean distribution) before the LPG in Pleistocene [35,36,88].

Therefore, all these results were congruent with the native origin of WCC populations of the Iberian Peninsula, as previously pointed out by Pedraza-Lara et al. [36], Jelić et al. [23] and Martin-Torrijos et al. [39], while the hypothesis of an introduced origin shows to be highly improbable. Clavero et al. [40] claimed, based on historical documents, that the founder event of the crayfish in the Iberia Peninsula originates from an introduction from the Duchy of Tuscany (Italy) to Spain at the end of the XVI century, an event that was followed by multiple introductions from Italy in the XVII and XVIII centuries (these latter without providing any reference). Even if the Iberian populations sharing haplotypes with Italy originated from there, this introduction could not explain the overall genetic diversity and structure of the WCC found in the Iberian Peninsula. Historical data might provide us with relevant information to help us to reconstruct the evolutionary history of species, but also they can easily avoid refutation through “ad hoc” hypotheses by simply modifying the dates or localities of colonization. Therefore, genetic evidences are always needed. For example, genetic studies on the European noble crayfish, *Astacus astacus* have shown that claims of an anthropogenic origin of this species in Scandinavia were the result of wrong translations of historical documents [93].

Our results suggest the effects of the massive extinction events that the Iberian WCC have suffered during the last decades and in the past. The reduction of genetic diversity leads to the loss of adaptive potential [94,95], and increases the risk of species extinction. However, the WCC Iberian populations still maintain a high structured genetic diversity. We can distinguish four main genetic groups clustering most of the populations, while the other 11 groups harbor a small number of populations and are genetically differentiated. The Iberian Peninsula showed a high number of unique haplotypes distributed among the northern and central-eastern regions. The results generated in this study are of key importance for the conservation of the WCC populations, since we can: (i) define evolutionary significant units based on knowledge of the Iberian genetic diversity, in order to prioritize the conservation of genetic groups and unique haplotypes and maintain the currently genetic diversity, (ii) concentrate the conservation resource in hot spot areas, such as these ones that host the most diverse genetic groups as revealed by the structure analyses, (iii) correlate haplotypes and genetic groups with populations that show a potential increase in resistance/or tolerance to the crayfish plague, as recently detected [83,84], and, finally, (iv) develop breeding and re-stocking programs that maximize the genetic diversity and the capacity to face the disease, and, consequently, the adaptive potential of the WCC populations. In addition, this study emphasizes the importance of establishing a consensus methodology to resolve questions related to the genetic diversity and structure of this crayfish species based on the use of few genes, avoiding thus new misunderstandings that could happen in the future.

Finally, we point out that there is also a need for unraveling the mitochondrial incongruence found in GIR5 population [83]. This population is particularly relevant because has been demonstrated to be the most increased resistant crayfish population from Europe to a highly virulent *A*. *astaci* haplotype (*i*.*e*., d2-haplotype) [83]. In this population, inconsistencies have been detected when analyzing mtDNA regions since several of their individuals belong to two different genetic clades (either to the *A*. *i*. *meridionalis* clade as judged by *16S* region or to *A*. *i*. *italicus* clade as judged by *COI* region). Moreover, the *COI* region has showed double peaks in some variable positions. Once contamination has been discarded as the cause for the presence of double peaks in the *COI* region sequences, the obtained results appear to indicate the existence of different mtDNA sequences within these crayfishes. This phenomenon of heteroplasmy has been recently reported in many organisms, including crustaceans such as the spiny spider crab *Maja brachydactyla* [96,97], or the blue crab *Callinectes sapidus* [98]. There are several reasons that can produce heteroplasmy: (i) *de novo* mutations, (ii) recombination events, (iii) paternal leakage, (iv) biparental inheritance or (v) doubly uniparental inheritance [96,99]. This process could be related with an interpopulation hybridization between *A*. *i*. *meridionalis* and *A*. *i*. *italicus* crayfishes, since both groups are inhabiting the northeastern of the Iberian Peninsula, near the Pyrenees. However, there are other two different possibilities that could be explained this situation. Firstly, the formation of nuclear mitochondrial pseudogenes (NUMTs) [100,101], which are described as the transposition of mitochondrial DNA into the nuclear genome that can retain close homology to the original mitochondrial genes [102]. There are also references to the presence of *COI*-like sequences in many crustaceans, including crayfish [103,104]. Secondly, could be due to a duplication in the mitochondrial genome [101]. Confirm the presence of heteroplasmy or another process is needed to interpret correctly our results. For instance, in genetic population studies, the assumption of maternal mitochondrial inheritance in species with multiple mtDNA copies per individual can provide an overestimation of the effective population size as well as create false negatives in lineage determination [96]. Further investigation is needed to clarify the causes of the mitochondrial incongruence found in these crayfishes, and more importantly to understand whether this genetic incongruence is related to an increased crayfish plague resistance.

## Supporting information

S1 Fig Double peaks in the cytochrome oxidase subunit I region of GIR5 population. Example of double peaks in the forward (up) and reverse (down) reads of from at positions 12 and 108.
(DOCX)

S2 Fig Phylogenetic tree based on the 16S rRNA region including GIR5 population. Bayesian inference phylogenetic tree based on the 16S rRNA region including the crayfishes from GIR5 population (in blue) and the known haplotypes of the WCC from dataset 1 (sequences from the whole geographical distribution range, covering all lineages and clades previously defined to the WCC species complex). Posterior probabilities values are shown above the branches.
(DOCX)

S3 Fig Phylogenetic tree based on the *COI* mtDNA region including GIR5 population. Bayesian inference phylogenetic tree based on the *COI* mtDNA region including the crayfishes from GIR5 population (in blue) and the known haplotypes of the WCC from dataset 1 (sequences from the whole geographical distribution range, covering all lineages and clades previously defined to the WCC species complex). Posterior probabilities values are shown above the branches.
(DOCX)

S4 Fig Complete Bayesian phylogenetic tree based on low rates scenario from dataset 1. Complete version of the Bayesian inference phylogenetic tree with the divergence time estimates for the haplotypes of the white-clawed crayfish species based on the low rates scenario from dataset 1 (sequences from the whole geographical distribution range, covering all lineages and clades previously defined to the WCC species complex) based on 948 bp of the concatenated mitochondrial 16S rRNA and cytochrome oxidase subunit I regions. Node branches show the estimated mean and range of the divergence time in Mya. The nodes support with high posterior probability values (pp≥0.95) show a black circle, and the divergence times are in bold.
(TIF)

S5 Fig Complete Bayesian phylogenetic tree based on intermediate rates scenario from dataset 1. Complete version of the Bayesian inference phylogenetic tree with the divergence time estimates for the haplotypes of the white-clawed crayfish species based on the intermediate rates scenario from dataset 1 (sequences from the whole geographical distribution range, covering all lineages and clades previously defined to the WCC species complex) based on 948 bp of the concatenated mitochondrial 16S rRNA and cytochrome oxidase subunit I regions. Node branches show the estimated mean and range of the divergence time in Mya. The nodes support with high posterior probability values (pp≥0.95) show a black circle, and the divergence times are in bold.
(TIF)

S6 Fig Complete Bayesian phylogenetic tree based on high rates scenario from dataset 1. Complete version of the Bayesian inference phylogenetic tree with the divergence time estimates for the haplotypes of the white-clawed crayfish species based on the high rates scenario from dataset 1 (sequences from the whole geographical distribution range, covering all lineages and clades previously defined to the WCC species complex) based on 948 bp of the concatenated mitochondrial 16S rRNA and cytochrome oxidase subunit I regions. Node branches show the estimated mean and range of the divergence time in Mya. The nodes support with high posterior probability values (pp≥0.95) show a black circle, and the divergence times are in bold.
(TIF)

S1 Table Populations and localities. Populations and locations of the white-clawed crayfish analyzed in the present study (population and individual ID, dataset in which included, collection reference, GenBank accession number, and location).
(DOCX)

S2 Table Positions (base pair) with double peaks of the cytochrome oxidase subunit I region from the GIR5 population. Nucleotides of the obtained sequences of the cytochrome oxidase subunit I region from the GIR5 population in positions with double peaks (on the left) in alignment with the haplotype sequences obtained for dataset 1 (sequences from the whole geographical distribution range, covering all lineages and clades previously defined to the WCC species complex). Dots indicate identity with the consensus sequence, while the variable position that showed double peaks appears in parenthesis. Asterisks indicate nucleotides previously unreported in that position.
(DOCX)

S3 Table Dataset 1 genetic diversity indices. Genetic diversity indices of the populations from dataset 1 (sequences from the whole geographical distribution range, covering all lineages and clades previously defined to the WCC species complex) based on 948 bp of the concatenated mitochondrial 16S rRNA and cytochrome oxidase subunit I regions: sample size (n), the number of polymorphic sites (S), the number of haplotypes (H), the haplotype diversity (Hd), the nucleotide diversity (π), the Tajima’s D (D), and Fu’s Fs (Fs).
(DOCX)

S4 Table Dataset 2 genetic diversity indices. Genetic diversity indices of the populations from dataset 2 (sequences from the Iberian and Italian peninsulas) based on 2449 bp of the concatenated mitochondrial *16S* rRNA and cytochrome oxidase subunit I regions: sample size (n), the number of polymorphic sites (S), the number of haplotypes (H), the haplotype diversity (Hd), the nucleotide diversity (π), the Tajima’s D (D), and Fu’s Fs (Fs).
(DOCX)

S5 Table Genetic structure population groups for dataset 1. Population groups obtained for the optimum genetic structure (K = 20) of the dataset 1 (sequences from the whole geographical distribution range, covering all lineages and clades previously defined to the WCC species complex) based on 948 bp of the concatenated mitochondrial 16S rRNA and cytochrome oxidase subunit I regions.
(DOCX)

S6 Table Genetic structure population groups for dataset 2. Population groups obtained for the optimum genetic structure (K = 18) of the dataset 2 (sequences from the Iberian and Italian peninsulas) based on 2449 bp of the concatenated mitochondrial 16S rRNA and cytochrome oxidase subunit I regions.
(DOCX)
