# Supplementary material for: On the conservation of white-clawed crayfish in the Iberian Peninsula: Unraveling its genetic diversity and structure, and origin
Source: PLoS One. 2023 Oct 13;18(10):e0292679. doi: 10.1371/journal.pone.0292679 (PMC10575519; doi:10.1371/journal.pone.0292679)
Supplement: S1 Fig — Example of double peaks in the forward (up) and reverse (down) reads of from at positions 12 and 108. (DOCX) [file pone.0292679.s001.docx]

**
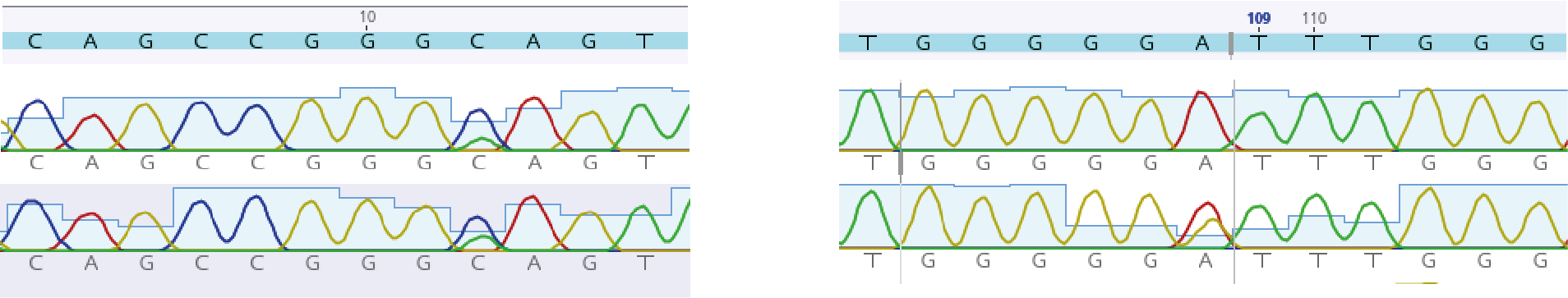
**

**S1 Fig. Double peaks in the cytochrome oxidase subunit I region of GIR5 population**. Example of double peaks in the forward (up) and reverse (down) reads of from at positions 12 and 108.
