# Supplementary material for: On the conservation of white-clawed crayfish in the Iberian Peninsula: Unraveling its genetic diversity and structure, and origin
Source: PLoS One. 2023 Oct 13;18(10):e0292679. doi: 10.1371/journal.pone.0292679 (PMC10575519; doi:10.1371/journal.pone.0292679)
Supplement: S3 Fig — Bayesian inference phylogenetic tree based on the COI mtDNA region including the crayfishes from GIR5 population (in blue) and the known haplotypes of the WCC from dataset 1 (sequences from the whole geographical distribution range, covering all lineages and clades previously defined to the WCC species complex). Posterior probabilities values are shown above the branches. (DOCX) [file pone.0292679.s003.docx]

**
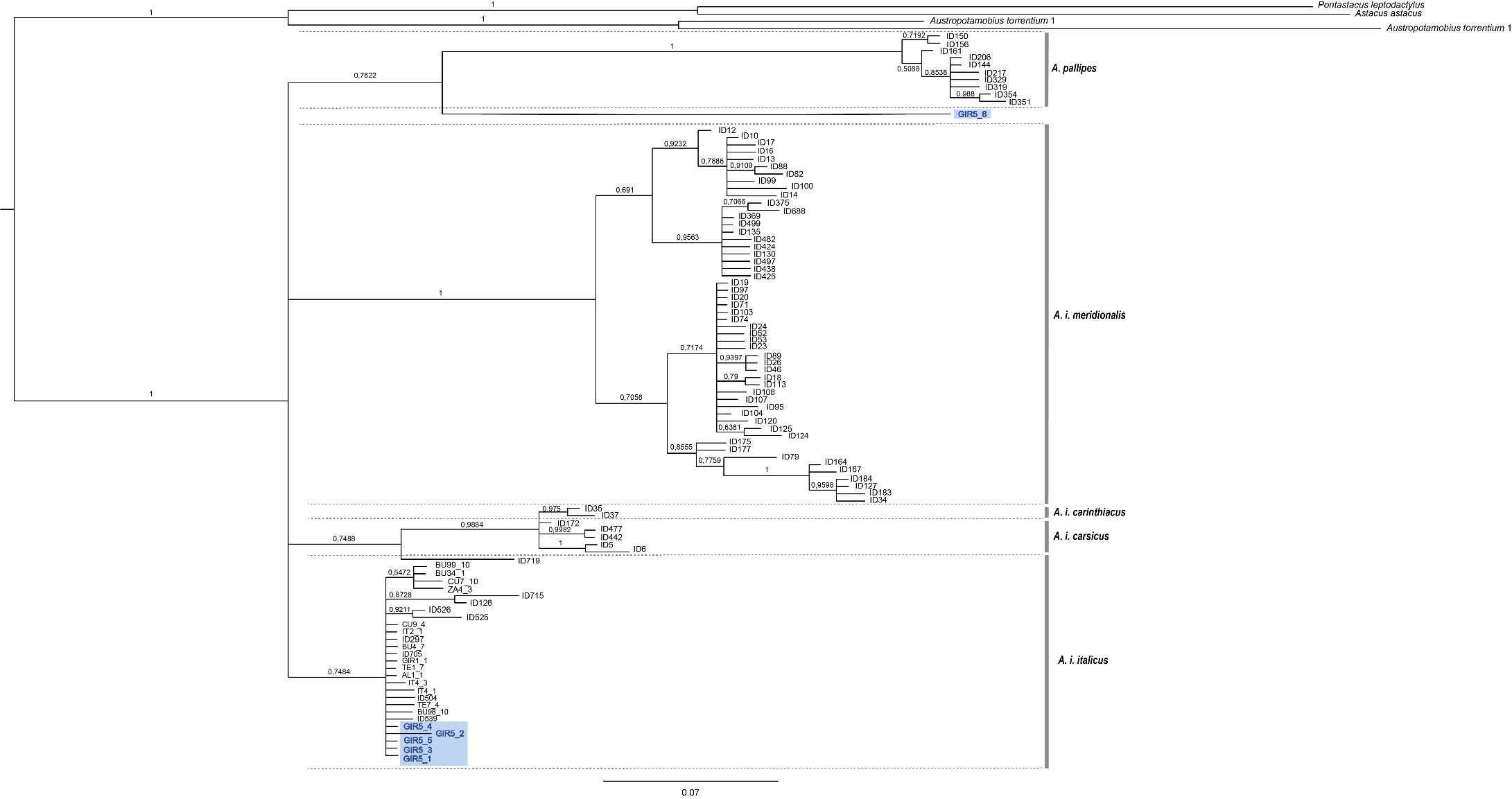
**

**S3 Fig**. **Phylogenetic tree based on the *COI* mtDNA** **region including GIR5 population.** Bayesian inference phylogenetic tree based on the *COI* mtDNA region including the crayfishes from GIR5 population (in blue) and the known haplotypes of the WCC from dataset 1 (sequences from the whole geographical distribution range, covering all lineages and clades previously defined to the WCC species complex). Posterior probabilities values are shown above the branches.
