# Supplementary figures and images for: On the conservation of white-clawed crayfish in the Iberian Peninsula: Unraveling its genetic diversity and structure, and origin
Source: PLoS One. 2023 Oct 13;18(10):e0292679. doi: 10.1371/journal.pone.0292679 (PMC10575519; doi:10.1371/journal.pone.0292679)

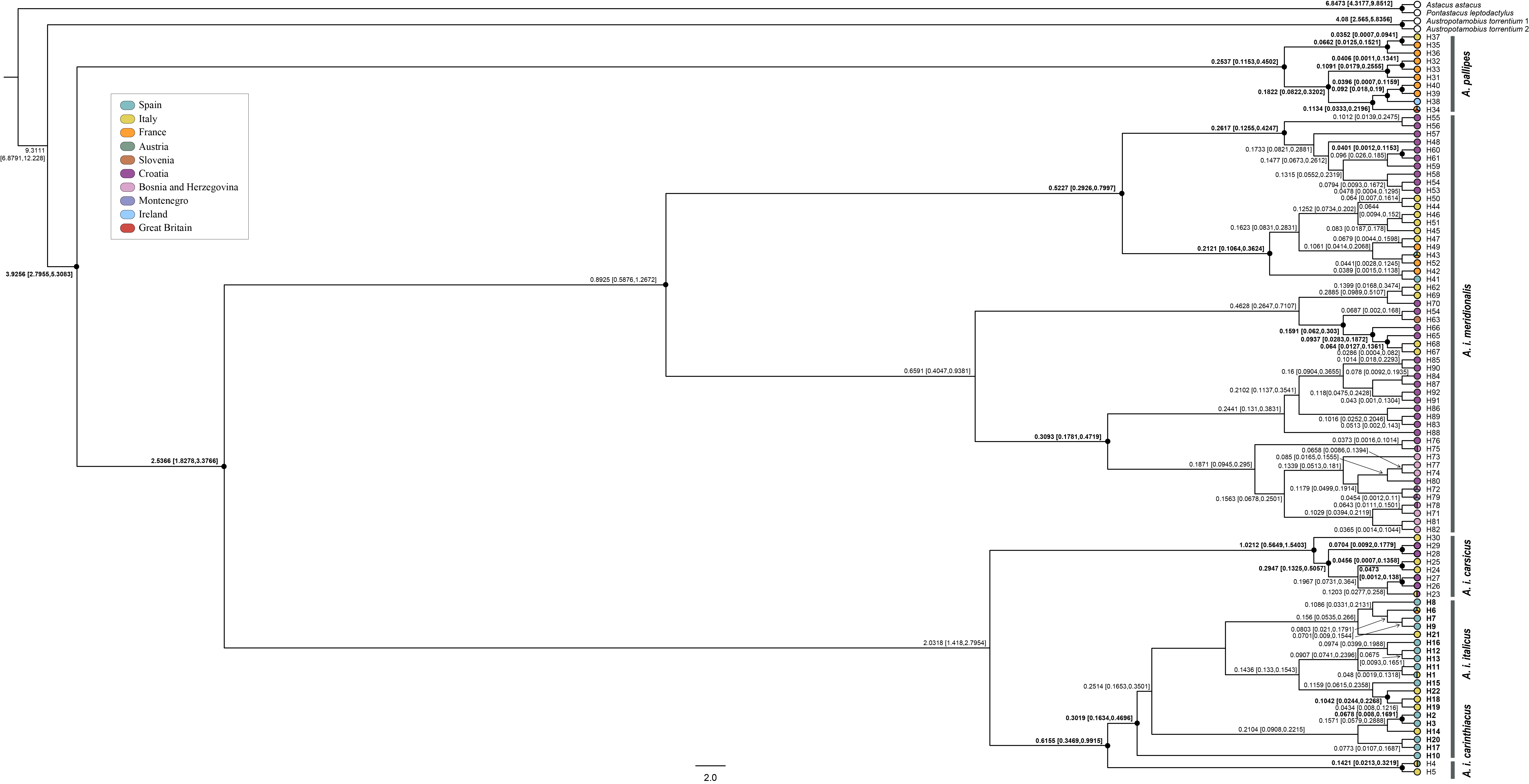

Supplement: S4 Fig — Complete version of the Bayesian inference phylogenetic tree with the divergence time estimates for the haplotypes of the white-clawed crayfish species based on the low rates scenario from dataset 1 (sequences from the whole geographical distribution range, covering all lineages and clades previously defined to the WCC species complex) based on 948 bp of the concatenated mitochondrial 16S rRNA and cytochrome oxidase subunit I regions. Node branches show the estimated mean and range of the divergence time in Mya. The nodes support with high posterior probability values (pp≥0.95) show a black circle, and the divergence times are in bold. (TIF) [file pone.0292679.s004.tif]

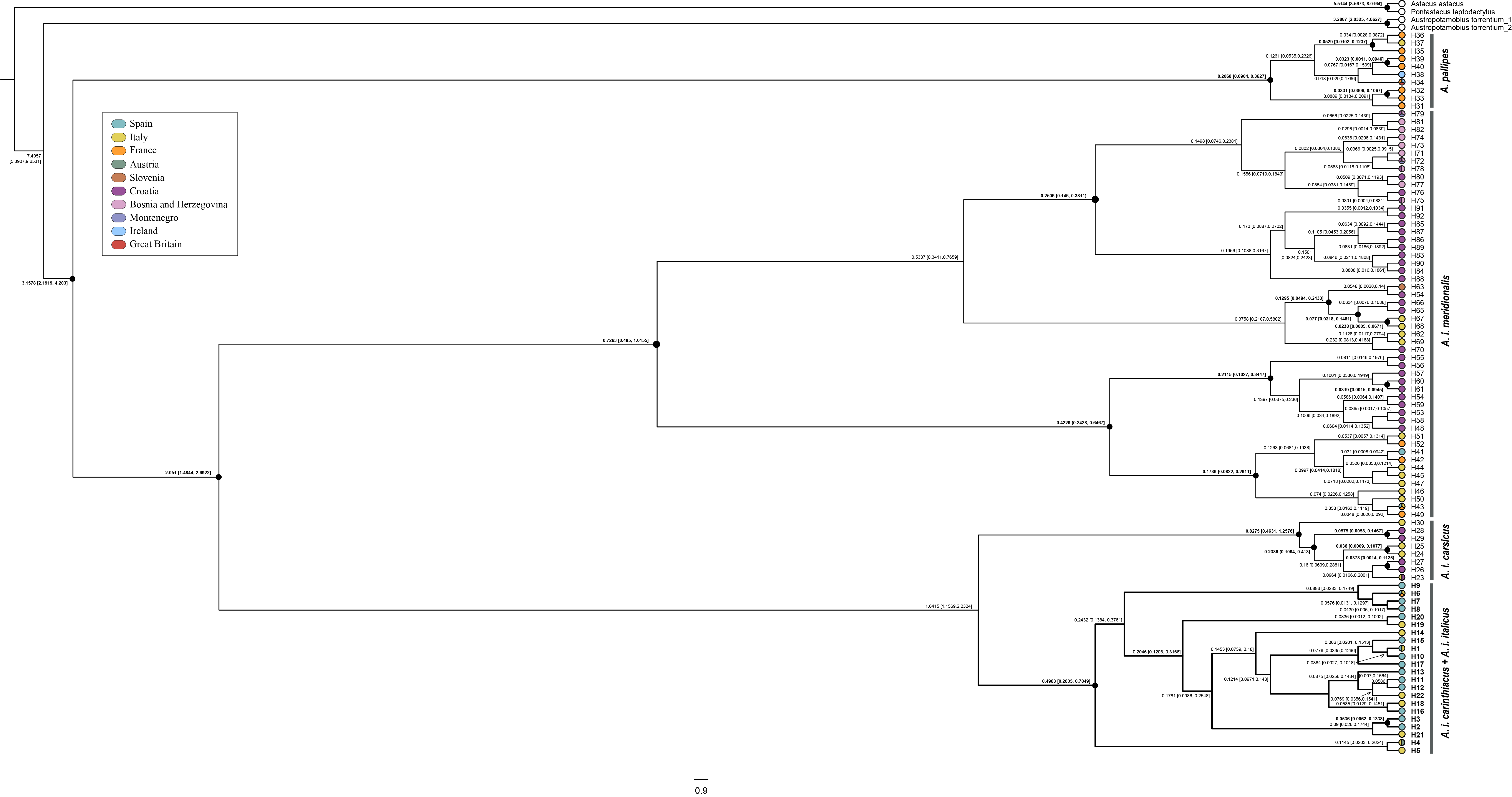

Supplement: S5 Fig — Complete version of the Bayesian inference phylogenetic tree with the divergence time estimates for the haplotypes of the white-clawed crayfish species based on the intermediate rates scenario from dataset 1 (sequences from the whole geographical distribution range, covering all lineages and clades previously defined to the WCC species complex) based on 948 bp of the concatenated mitochondrial 16S rRNA and cytochrome oxidase subunit I regions. Node branches show the estimated mean and range of the divergence time in Mya. The nodes support with high posterior probability values (pp≥0.95) show a black circle, and the divergence times are in bold. (TIF) [file pone.0292679.s005.tif]

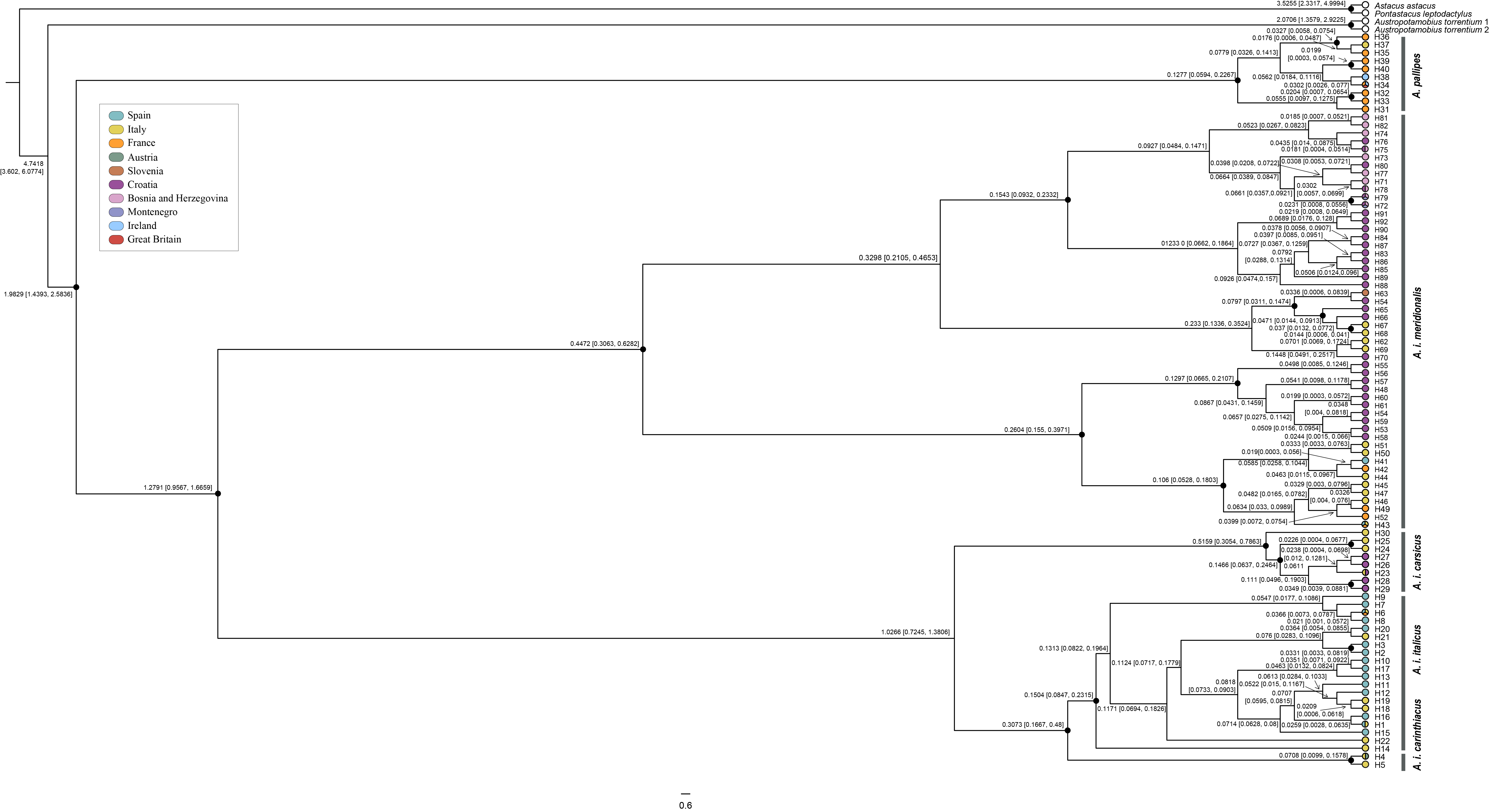

Supplement: S6 Fig — Complete version of the Bayesian inference phylogenetic tree with the divergence time estimates for the haplotypes of the white-clawed crayfish species based on the high rates scenario from dataset 1 (sequences from the whole geographical distribution range, covering all lineages and clades previously defined to the WCC species complex) based on 948 bp of the concatenated mitochondrial 16S rRNA and cytochrome oxidase subunit I regions. Node branches show the estimated mean and range of the divergence time in Mya. The nodes support with high posterior probability values (pp≥0.95) show a black circle, and the divergence times are in bold. (TIF) [file pone.0292679.s006.tif]
