# Supplementary material for: On the conservation of white-clawed crayfish in the Iberian Peninsula: Unraveling its genetic diversity and structure, and origin
Source: PLoS One. 2023 Oct 13;18(10):e0292679. doi: 10.1371/journal.pone.0292679 (PMC10575519; doi:10.1371/journal.pone.0292679)
Supplement: S1 Table — Populations and locations of the white-clawed crayfish analyzed in the present study (population and individual ID, dataset in which included, collection reference, GenBank accession number, and location). (DOCX) [file pone.0292679.s007.docx]

**S1 Table**. **Populations and localities.** Populations and locations of the white-clawed crayfish analyzed in the present study (population and individual ID, dataset in which included, collection reference, GenBank accession number, and location).

| **Population** |  |  |  | **Genbank access** | |  |
| --- | --- | --- | --- | --- | --- | --- |
|  | **ID** | **Data**  **set** | **Collection** | ***16S*** | ***COI*** | **Location** |
| **AL1** | AL1_1 | 1, 2 | Matallanas et al., 2016 | EF489427 | EF485041 | Spain: Altube (Álava) |
|  | AL1_2 | 1, 2 | Matallanas et al., 2016 | EF489427 | EF485041 | Spain: Altube (Álava) |
|  | AL1_3 | 1, 2 | Matallanas et al., 2016 | EF489427 | EF485041 | Spain: Altube (Álava) |
|  | AL1_4 | 1, 2 | Matallanas et al., 2016 | EF489427 | EF485041 | Spain: Altube (Álava) |
|  | AL1_5 | 1, 2 | Matallanas et al., 2016 | EF489427 | EF485041 | Spain: Altube (Álava) |
|  | AL1_6 | 1, 2 | Matallanas et al., 2016 | EF489427 | EF485041 | Spain: Altube (Álava) |
|  | AL1_7 | 1, 2 | Matallanas et al., 2016 | EF489427 | EF485041 | Spain: Altube (Álava) |
|  | AL1_8 | 1, 2 | Matallanas et al., 2016 | EF489427 | EF485041 | Spain: Altube (Álava) |
|  | AL1_9 | 1, 2 | Matallanas et al., 2016 | EF489427 | EF485041 | Spain: Altube (Álava) |
|  | AL1_10 | 1, 2 | Matallanas et al., 2016 | EF489427 | EF485041 | Spain: Altube (Álava) |
| **AL2** | ID566 | 1 | Pedraza-Lara et al., 2010 | HM622595 | HM622601 | Spain: Rio Zadoya (Álava) |
|  | ID567 | 1 | Pedraza-Lara et al., 2010 | HM622595 | HM622601 | Spain: Rio Zadoya (Álava) |
| **AL3** | ID646 | 1 | Pedraza-Lara et al., 2010 | HM622595 | HM622601 | Spain: Rio Nervion (Álava) |
|  | ID647 | 1 | Pedraza-Lara et al., 2010 | HM622595 | HM622601 | Spain: Rio Nervion (Álava) |
| **AS1** | AS1_1 | 1, 2 | Martín-Torrijos et al., 2021 | MW327384 | MW325589 | Spain: Cangas de Onis (Asturias) |
|  | AS1_3 | 1, 2 | Martín-Torrijos et al., 2021 | MW327412 | MW325590 | Spain: Cangas de Onis (Asturias) |
| **AS2** | AS2_1 | 1, 2 | Matallanas et al., 2016 | EF489427 | FJ897843 | Spain: Cangas de Onis (Asturias) |
|  | AS2_2 | 1, 2 | Matallanas et al., 2016 | EF489427 | FJ897843 | Spain: Cangas de Onis (Asturias) |
|  | AS2_3 | 1, 2 | Matallanas et al., 2016 | EF489427 | FJ897843 | Spain: Cangas de Onis (Asturias) |
|  | AS2_4 | 1, 2 | Matallanas et al., 2016 | EF489427 | FJ897843 | Spain: Cangas de Onis (Asturias) |
|  | AS2_5 | 1, 2 | Matallanas et al., 2016 | EF489427 | FJ897843 | Spain: Cangas de Onis (Asturias) |
|  | AS2_6 | 1, 2 | Matallanas et al., 2016 | EF489427 | FJ897843 | Spain: Cangas de Onis (Asturias) |
|  | AS2_7 | 1, 2 | Matallanas et al., 2016 | EF489427 | FJ897843 | Spain: Cangas de Onis (Asturias) |
|  | AS2_8 | 1, 2 | Matallanas et al., 2016 | EF489427 | FJ897843 | Spain: Cangas de Onis (Asturias) |
|  | AS2_9 | 1, 2 | Matallanas et al., 2016 | EF489427 | FJ897843 | Spain: Cangas de Onis (Asturias) |
|  | AS2_10 | 1, 2 | Matallanas et al., 2016 | EF489427 | FJ897843 | Spain: Cangas de Onis (Asturias) |
| **AS3** | AS3_1 | 1, 2 | Matallanas et al., 2016 | EF489427 | FJ897843 | Spain: Cangas de Onis (Asturias) |
|  | AS3_2 | 1, 2 | Matallanas et al., 2016 | EF489427 | FJ897843 | Spain: Cangas de Onis (Asturias) |
|  | AS3_3 | 1, 2 | Matallanas et al., 2016 | EF489427 | FJ897843 | Spain: Cangas de Onis (Asturias) |
|  | AS3_4 | 1, 2 | Matallanas et al., 2016 | EF489427 | FJ897843 | Spain: Cangas de Onis (Asturias) |
|  | AS3_5 | 1, 2 | Matallanas et al., 2016 | EF489427 | FJ897843 | Spain: Cangas de Onis (Asturias) |
|  | AS3_6 | 1, 2 | Matallanas et al., 2016 | EF489427 | FJ897843 | Spain: Cangas de Onis (Asturias) |
|  | AS3_7 | 1, 2 | Matallanas et al., 2016 | EF489427 | FJ897843 | Spain: Cangas de Onis (Asturias) |
|  | AS3_8 | 1, 2 | Matallanas et al., 2016 | EF489427 | FJ897843 | Spain: Cangas de Onis (Asturias) |
|  | AS3_9 | 1, 2 | Matallanas et al., 2016 | EF489427 | FJ897843 | Spain: Cangas de Onis (Asturias) |
|  | AS3_10 | 1, 2 | Matallanas et al., 2016 | EF489427 | FJ897843 | Spain: Cangas de Onis (Asturias) |
| **AS4** | ID648 | 1 | Pedraza-Lara et al., 2010 | HM622595 | HM622601 | Spain: Lake Ercina (Asturias) |
|  | ID649 | 1 | Pedraza-Lara et al., 2010 | HM622595 | HM622601 | Spain: Lake Ercina (Asturias) |
|  | ID650 | 1 | Pedraza-Lara et al., 2010 | HM622595 | HM622601 | Spain: Lake Ercina (Asturias) |
|  | ID651 | 1 | Pedraza-Lara et al., 2010 | HM622595 | HM622601 | Spain: Lake Ercina (Asturias) |
| **AUS1** | ID126 | 1 | Jelić et al., 2016 | KX370214 | KX369794 | Austria: Plansee Lake |
|  | ID377 | 1 | Jelić et al., 2016 | KX370465 | KX370045 | Austria: Plansee Lake |
|  | ID378 | 1 | Jelić et al., 2016 | KX370466 | KX370046 | Austria: Plansee Lake |
|  | ID379 | 1 | Jelić et al., 2016 | KX370467 | KX370047 | Austria: Plansee Lake |
|  | ID380 | 1 | Jelić et al., 2016 | KX370468 | KX370048 | Austria: Plansee Lake |
|  | ID381 | 1 | Jelić et al., 2016 | KX370469 | KX370049 | Austria: Plansee Lake |
|  | ID382 | 1 | Jelić et al., 2016 | KX370470 | KX370050 | Austria: Plansee Lake |
|  | ID383 | 1 | Jelić et al., 2016 | KX370471 | KX370051 | Austria: Plansee Lake |
|  | ID384 | 1 | Jelić et al., 2016 | KX370472 | KX370052 | Austria: Plansee Lake |
|  | ID385 | 1 | Jelić et al., 2016 | KX370473 | KX370053 | Austria: Plansee Lake |
|  | ID386 | 1 | Jelić et al., 2016 | KX370474 | KX370054 | Austria: Plansee Lake |
|  | ID387 | 1 | Jelić et al., 2016 | KX370475 | KX370055 | Austria: Plansee Lake |
|  | ID388 | 1 | Jelić et al., 2016 | KX370476 | KX370056 | Austria: Plansee Lake |
|  | ID389 | 1 | Jelić et al., 2016 | KX370477 | KX370057 | Austria: Plansee Lake |
|  | ID390 | 1 | Jelić et al., 2016 | KX370478 | KX370058 | Austria: Plansee Lake |
|  | ID391 | 1 | Jelić et al., 2016 | KX370479 | KX370059 | Austria: Plansee Lake |
|  | ID392 | 1 | Jelić et al., 2016 | KX370480 | KX370060 | Austria: Plansee Lake |
|  | ID393 | 1 | Jelić et al., 2016 | KX370481 | KX370061 | Austria: Plansee Lake |
|  | ID394 | 1 | Jelić et al., 2016 | KX370482 | KX370062 | Austria: Plansee Lake |
|  | ID395 | 1 | Jelić et al., 2016 | KX370483 | KX370063 | Austria: Plansee Lake |
|  | ID396 | 1 | Jelić et al., 2016 | KX370484 | KX370064 | Austria: Plansee Lake |
|  | ID397 | 1 | Jelić et al., 2016 | KX370485 | KX370065 | Austria: Plansee Lake |
| **AV1** | AV1_1 | 1, 2 | Martín-Torrijos et al., 2021 | MW327326 | MW325420 | Spain: Sanchorreja (Ávila) |
|  | AV1_2 | 1, 2 | Martín-Torrijos et al., 2021 | MW327327 | MW325421 | Spain: Sanchorreja (Ávila) |
|  | AV1_3 | 1, 2 | Martín-Torrijos et al., 2021 | MW327328 | MW325422 | Spain: Sanchorreja (Ávila) |
|  | AV1_4 | 1, 2 | Martín-Torrijos et al., 2021 | MW327329 | MW325434 | Spain: Sanchorreja (Ávila) |
|  | AV1_5 | 1, 2 | Martín-Torrijos et al., 2021 | MW327290 | MW325423 | Spain: Sanchorreja (Ávila) |
|  | AV1_7 | 1, 2 | Martín-Torrijos et al., 2021 | MW327286 | MW325436 | Spain: Sanchorreja (Ávila) |
|  | AV1_8 | 1, 2 | Martín-Torrijos et al., 2021 | MW327274 | MW325371 | Spain: Sanchorreja (Ávila) |
|  | AV1_9 | 1, 2 | Martín-Torrijos et al., 2021 | MW327275 | MW325372 | Spain: Sanchorreja (Ávila) |
|  | AV1_10 | 1, 2 | Martín-Torrijos et al., 2021 | MW327276 | MW325373 | Spain: Sanchorreja (Ávila) |
| **AV2** | AV2_1 | 1, 2 | Martín-Torrijos et al., 2021 | MW327197 | MW325433 | Spain: Santa María del Cubillo (Ávila) |
|  | AV2_2 | 1, 2 | Martín-Torrijos et al., 2021 | MW327292 | MW325417 | Spain: Santa María del Cubillo (Ávila) |
|  | AV2_3 | 1, 2 | Martín-Torrijos et al., 2021 | MW327293 | MW325416 | Spain: Santa María del Cubillo (Ávila) |
|  | AV2_4 | 1, 2 | Martín-Torrijos et al., 2021 | MW327330 | MW325418 | Spain: Santa María del Cubillo (Ávila) |
|  | AV2_5 | 1, 2 | Martín-Torrijos et al., 2021 | MW327331 | MW325419 | Spain: Santa María del Cubillo (Ávila) |
|  | AV2_8 | 1, 2 | Martín-Torrijos et al., 2021 | MW327332 | MW325368 | Spain: Santa María del Cubillo (Ávila) |
|  | AV2_9 | 1, 2 | Martín-Torrijos et al., 2021 | MW327332 | MW325370 | Spain: Santa María del Cubillo (Ávila) |
|  | AV2_10 | 1, 2 | Martín-Torrijos et al., 2021 | MW327334 | MW325369 | Spain: Santa María del Cubillo (Ávila) |
| **AV3** | AV3_1 | 1, 2 | Martín-Torrijos et al., 2021 | MW327350 | MW325424 | Spain: Sanchorreja (Ávila) |
|  | AV3_2 | 1, 2 | Martín-Torrijos et al., 2021 | MW327351 | MW325435 | Spain: Sanchorreja (Ávila) |
|  | AV3_3 | 1, 2 | Martín-Torrijos et al., 2021 | MW327352 | MW325425 | Spain: Sanchorreja (Ávila) |
|  | AV3_4 | 1, 2 | Martín-Torrijos et al., 2021 | MW327353 | MW325427 | Spain: Sanchorreja (Ávila) |
|  | AV3_5 | 1, 2 | Martín-Torrijos et al., 2021 | MW327354 | MW325426 | Spain: Sanchorreja (Ávila) |
|  | AV3_6 | 1, 2 | Martín-Torrijos et al., 2021 | MW327318 | MW325390 | Spain: Sanchorreja (Ávila) |
|  | AV3_8 | 1, 2 | Martín-Torrijos et al., 2021 | MW327355 | MW325393 | Spain: Sanchorreja (Ávila) |
|  | AV3_9 | 1, 2 | Martín-Torrijos et al., 2021 | MW327319 | MW325391 | Spain: Sanchorreja (Ávila) |
|  | AV3_10 | 1, 2 | Martín-Torrijos et al., 2021 | MW327272 | MW325392 | Spain: Sanchorreja (Ávila) |
| **BCN1** | ID693 | 1 | Pedraza-Lara et al., 2010 | HM622595 | HM622608 | Spain: Fondrats |
|  | ID694 | 1 | Pedraza-Lara et al., 2010 | HM622595 | HM622614 | Spain: Fondrats |
|  | ID695 | 1 | Pedraza-Lara et al., 2010 | HM622595 | HM622614 | Spain: Fondrats |
|  | ID696 | 1 | Pedraza-Lara et al., 2010 | HM622595 | HM622615 | Spain: Fondrats |
| **BCN2** | ID703 | 1 | Pedraza-Lara et al., 2010 | HM622595 | HM622601 | Spain: Pujol Avenco |
|  | ID704 | 1 | Pedraza-Lara et al., 2010 | HM622595 | HM622601 | Spain: Pujol Avenco |
| **BOS2** | ID67 | 1 | Jelić et al., 2016 | KX370155 | KX369735 | Bosnia and Herzegovina: Žukovica River |
|  | ID68 | 1 | Jelić et al., 2016 | KX370156 | KX369736 | Bosnia and Herzegovina: Žukovica River |
|  | ID69 | 1 | Jelić et al., 2016 | KX370157 | KX369737 | Bosnia and Herzegovina: Žukovica River |
| **BOS3** | ID70 | 1 | Jelić et al., 2016 | KX370158 | KX369738 | Bosnia and Herzegovina: Vriošica River |
|  | ID71 | 1 | Jelić et al., 2016 | KX370159 | KX369739 | Bosnia and Herzegovina: Vriošica River |
|  | ID72 | 1 | Jelić et al., 2016 | KX370160 | KX369740 | Bosnia and Herzegovina: Vriošica River |
| **BOS4** | ID73 | 1 | Jelić et al., 2016 | KX370161 | KX369741 | Bosnia and Herzegovina: Tihaljina River |
|  | ID74 | 1 | Jelić et al., 2016 | KX370162 | KX369742 | Bosnia and Herzegovina: Tihaljina River |
|  | ID75 | 1 | Jelić et al., 2016 | KX370163 | KX369743 | Bosnia and Herzegovina: Tihaljina River |
| **BOS5** | ID104 | 1 | Jelić et al., 2016 | KX370192 | KX369772 | Bosnia and Herzegovina: Tribistovo |
|  | ID105 | 1 | Jelić et al., 2016 | KX370193 | KX369773 | Bosnia and Herzegovina: Tribistovo |
|  | ID106 | 1 | Jelić et al., 2016 | KX370194 | KX369774 | Bosnia and Herzegovina: Tribistovo |
| **BOS6** | ID107 | 1 | Jelić et al., 2016 | KX370195 | KX369775 | Bosnia and Herzegovina: Bilila Mokrašnica |
|  | ID108 | 1 | Jelić et al., 2016 | KX370196 | KX369776 | Bosnia and Herzegovina: Bilila Mokrašnica |
| **BOS7** | ID109 | 1 | Jelić et al., 2016 | KX370197 | KX369777 | Bosnia and Herzegovina: Zorića Gaj |
|  | ID110 | 1 | Jelić et al., 2016 | KX370198 | KX369778 | Bosnia and Herzegovina: Zorića Gaj |
|  | ID111 | 1 | Jelić et al., 2016 | KX370199 | KX369779 | Bosnia and Herzegovina: Zorića Gaj |
|  | ID112 | 1 | Jelić et al., 2016 | KX370200 | KX369780 | Bosnia and Herzegovina: Zorića Gaj |
| **BOS8** | ID118 | 1 | Jelić et al., 2016 | KX370206 | KX369786 | Bosnia and Herzegovina: Jelica |
|  | ID119 | 1 | Jelić et al., 2016 | KX370207 | KX369787 | Bosnia and Herzegovina: Jelica |
| **BOS9** | ID120 | 1 | Jelić et al., 2016 | KX370208 | KX369788 | Bosnia and Herzegovina: Gaj |
|  | ID121 | 1 | Jelić et al., 2016 | KX370209 | KX369789 | Bosnia and Herzegovina: Gaj |
| **BOS10** | ID124 | 1 | Jelić et al., 2016 | KX370212 | KX369792 | Bosnia and Herzegovina: Mrtvica River |
|  | ID125 | 1 | Jelić et al., 2016 | KX370213 | KX369793 | Bosnia and Herzegovina: Mrtvica River |
| **BU4** | BU4_1 | 1, 2 | Martín-Torrijos et al., 2021 | MW327427 | MW325601 | Spain: Rebolledo de la Torre (Burgos) |
|  | BU4_10 | 1, 2 | Martín-Torrijos et al., 2021 | MW327452 | MW325596 | Spain: Rebolledo de la Torre (Burgos) |
|  | BU4_2 | 1, 2 | Martín-Torrijos et al., 2021 | MW327402 | MW325602 | Spain: Rebolledo de la Torre (Burgos) |
|  | BU4_3 | 1, 2 | Martín-Torrijos et al., 2021 | MW327455 | MW325603 | Spain: Rebolledo de la Torre (Burgos) |
|  | BU4_4 | 1, 2 | Martín-Torrijos et al., 2021 | MW327429 | MW325604 | Spain: Rebolledo de la Torre (Burgos) |
|  | BU4_5 | 1, 2 | Martín-Torrijos et al., 2021 | MW327403 | MW325608 | Spain: Rebolledo de la Torre (Burgos) |
|  | BU4_6 | 1, 2 | Martín-Torrijos et al., 2021 | MW327437 | MW325609 | Spain: Rebolledo de la Torre (Burgos) |
|  | BU4_7 | 1, 2 | Martín-Torrijos et al., 2021 | MW327456 | MW325594 | Spain: Rebolledo de la Torre (Burgos) |
|  | BU4_8 | 1, 2 | Martín-Torrijos et al., 2021 | MW327447 | MW325595 | Spain: Rebolledo de la Torre (Burgos) |
|  | BU4_9 | 1, 2 | Martín-Torrijos et al., 2021 | MW327438 | MW325607 | Spain: Rebolledo de la Torre (Burgos) |
| **BU7** | BU7_1 | 1, 2 | Martín-Torrijos et al., 2021 | MW327449 | MW325543 | Spain: Santa María del Campo (Burgos) |
|  | BU7_2 | 1, 2 | Martín-Torrijos et al., 2021 | MW327450 | MW325562 | Spain: Santa María del Campo (Burgos) |
|  | BU7_4 | 1, 2 | Martín-Torrijos et al., 2021 | MW327420 | MW325544 | Spain: Santa María del Campo (Burgos) |
| **BU22** | BU22_1 | 1, 2 | Martín-Torrijos et al., 2021 | MW327263 | MW32538 | Spain: Fuentenebro (Burgos) |
|  | BU22_10 | 1, 2 | Martín-Torrijos et al., 2021 | MW327460 | MW325388 | Spain: Fuentenebro (Burgos) |
|  | BU22_2 | 1, 2 | Martín-Torrijos et al., 2021 | MW327459 | MW325382 | Spain: Fuentenebro (Burgos) |
|  | BU22_3 | 1, 2 | Martín-Torrijos et al., 2021 | MW327325 | MW325383 | Spain: Fuentenebro (Burgos) |
|  | BU22_4 | 1, 2 | Martín-Torrijos et al., 2021 | MW327381 | MW325389 | Spain: Fuentenebro (Burgos) |
|  | BU22_5 | 1, 2 | Martín-Torrijos et al., 2021 | MW327375 | MW325384 | Spain: Fuentenebro (Burgos) |
|  | BU22_6 | 1, 2 | Martín-Torrijos et al., 2021 | MW327347 | MW325385 | Spain: Fuentenebro (Burgos) |
|  | BU22_7 | 1, 2 | Martín-Torrijos et al., 2021 | MW327376 | MW325386 | Spain: Fuentenebro (Burgos) |
|  | BU22_9 | 1, 2 | Martín-Torrijos et al., 2021 | MW327382 | MW325387 | Spain: Fuentenebro (Burgos) |
| **BU34** | BU34_1 | 1, 2 | Martín-Torrijos et al., 2021 | MW327198 | MW325510 | Spain: Hontoria de Valdearados (Burgos) |
|  | BU34_10 | 1, 2 | Martín-Torrijos et al., 2021 | MW327336 | MW325514 | Spain: Hontoria de Valdearados (Burgos) |
|  | BU34_2 | 1, 2 | Martín-Torrijos et al., 2021 | MW327199 | MW325511 | Spain: Hontoria de Valdearados (Burgos) |
|  | BU34_3 | 1, 2 | Martín-Torrijos et al., 2021 | MW327294 | MW325512 | Spain: Hontoria de Valdearados (Burgos) |
|  | BU34_4 | 1, 2 | Martín-Torrijos et al., 2021 | MW327200 | MW325513 | Spain: Hontoria de Valdearados (Burgos) |
|  | BU34_8 | 1, 2 | Martín-Torrijos et al., 2021 | MW327335 | MW325525 | Spain: Hontoria de Valdearados (Burgos) |
| **BU53** | BU53_1 | 1, 2 | Martín-Torrijos et al., 2021 | MW327426 | MW325552 | Spain: Santo Domindo de Silos (Burgos) |
|  | BU53_10 | 1, 2 | Martín-Torrijos et al., 2021 | MW327461 | MW325555 | Spain: Santo Domindo de Silos (Burgos) |
|  | BU53_2 | 1, 2 | Martín-Torrijos et al., 2021 | MW327457 | MW325553 | Spain: Santo Domindo de Silos (Burgos) |
|  | BU53_3 | 1, 2 | Martín-Torrijos et al., 2021 | MW327404 | MW325556 | Spain: Santo Domindo de Silos (Burgos) |
|  | BU53_4 | 1, 2 | Martín-Torrijos et al., 2021 | MW327453 | MW325557 | Spain: Santo Domindo de Silos (Burgos) |
|  | BU53_8 | 1, 2 | Martín-Torrijos et al., 2021 | MW327451 | MW325566 | Spain: Santo Domindo de Silos (Burgos) |
|  | BU53_9 | 1, 2 | Martín-Torrijos et al., 2021 | MW327454 | MW325554 | Spain: Santo Domindo de Silos (Burgos) |
| **BU58** | BU58_1 | 1, 2 | Martín-Torrijos et al., 2021 | MW327434 | MW325600 | Spain: Santo Domindo de Silos (Burgos) |
|  | BU58_10 | 1, 2 | Martín-Torrijos et al., 2021 | MW327448 | MW325593 | Spain: Santo Domindo de Silos (Burgos) |
|  | BU58_2 | 1, 2 | Martín-Torrijos et al., 2021 | MW327442 | MW325563 | Spain: Santo Domindo de Silos (Burgos) |
|  | BU58_3 | 1, 2 | Martín-Torrijos et al., 2021 | MW327428 | MW325605 | Spain: Santo Domindo de Silos (Burgos) |
|  | BU58_4 | 1, 2 | Martín-Torrijos et al., 2021 | MW327405 | MW325606 | Spain: Santo Domindo de Silos (Burgos) |
|  | BU58_5 | 1, 2 | Martín-Torrijos et al., 2021 | MW327436 | MW325591 | Spain: Santo Domindo de Silos (Burgos) |
|  | BU58_6 | 1, 2 | Martín-Torrijos et al., 2021 | MW327441 | MW325558 | Spain: Santo Domindo de Silos (Burgos) |
|  | BU58_7 | 1, 2 | Martín-Torrijos et al., 2021 | MW327440 | MW325567 | Spain: Santo Domindo de Silos (Burgos) |
|  | BU58_8 | 1, 2 | Martín-Torrijos et al., 2021 | MW327439 | MW325592 | Spain: Santo Domindo de Silos (Burgos) |
|  | BU58_9 | 1, 2 | Martín-Torrijos et al., 2021 | MW327423 | MW325564 | Spain: Santo Domindo de Silos (Burgos) |
| **BU64** | BU64_1 | 1, 2 | Martín-Torrijos et al., 2021 | MW327348 | MW325374 | Spain: Arauzo de Miel (Burgos) |
|  | BU64_10 | 1, 2 | Martín-Torrijos et al., 2021 | MW327203 | MW325380 | Spain: Arauzo de Miel (Burgos) |
|  | BU64_2 | 1, 2 | Martín-Torrijos et al., 2021 | MW327377 | MW325375 | Spain: Arauzo de Miel (Burgos) |
|  | BU64_3 | 1, 2 | Martín-Torrijos et al., 2021 | MW327349 | MW325376 | Spain: Arauzo de Miel (Burgos) |
|  | BU64_5 | 1, 2 | Martín-Torrijos et al., 2021 | MW327287 | MW325451 | Spain: Arauzo de Miel (Burgos) |
|  | BU64_6 | 1, 2 | Martín-Torrijos et al., 2021 | MW327201 | MW325377 | Spain: Arauzo de Miel (Burgos) |
|  | BU64_7 | 1, 2 | Martín-Torrijos et al., 2021 | MW327378 | MW325437 | Spain: Arauzo de Miel (Burgos) |
|  | BU64_8 | 1, 2 | Martín-Torrijos et al., 2021 | MW327202 | MW325379 | Spain: Arauzo de Miel (Burgos) |
|  | BU64_9 | 1, 2 | Martín-Torrijos et al., 2021 | MW327371 | MW325378 | Spain: Arauzo de Miel (Burgos) |
| **BU82** | BU82_1 | 1, 2 | Martín-Torrijos et al., 2021 | MW327406 | MW325587 | Spain: San Zadornil (Burgos) |
|  | BU82_5 | 1, 2 | Martín-Torrijos et al., 2021 | MW327284 | MW325588 | Spain: San Zadornil (Burgos) |
| **BU83** | BU83_1 | 1, 2 | Martín-Torrijos et al., 2021 | MW327385 | MW325583 | Spain: San Zadornil (Burgos) |
|  | BU83_2 | 1, 2 | Martín-Torrijos et al., 2021 | MW327386 | MW325584 | Spain: San Zadornil (Burgos) |
|  | BU83_4 | 1, 2 | Martín-Torrijos et al., 2021 | MW327416 | MW325585 | Spain: San Zadornil (Burgos) |
|  | BU83_5 | 1, 2 | Martín-Torrijos et al., 2021 | MW327413 | MW325586 | Spain: San Zadornil (Burgos) |
| **BU84** | BU84_1 | 1, 2 | Martín-Torrijos et al., 2021 | MW327425 | MW325578 | Spain: San Zadornil (Burgos) |
|  | BU84_2 | 1, 2 | Martín-Torrijos et al., 2021 | MW327432 | MW325579 | Spain: San Zadornil (Burgos) |
|  | BU84_3 | 1, 2 | Martín-Torrijos et al., 2021 | MW327408 | MW325580 | Spain: San Zadornil (Burgos) |
|  | BU84_4 | 1, 2 | Martín-Torrijos et al., 2021 | MW327443 | MW325581 | Spain: San Zadornil (Burgos) |
|  | BU84_5 | 1, 2 | Martín-Torrijos et al., 2021 | MW327409 | MW325582 | Spain: San Zadornil (Burgos) |
| **BU85** | BU85_1 | 1, 2 | Martín-Torrijos et al., 2021 | MW327394 | MW325575 | Spain: San Zadornil (Burgos) |
|  | BU85_3 | 1, 2 | Martín-Torrijos et al., 2021 | MW327414 | MW325576 | Spain: San Zadornil (Burgos) |
|  | BU85_4 | 1, 2 | Martín-Torrijos et al., 2021 | MW327395 | MW325577 | Spain: San Zadornil (Burgos) |
| **BU86** | BU86_1 | 1, 2 | Martín-Torrijos et al., 2021 | MW327435 | MW325571 | Spain: San Zadornil (Burgos) |
|  | BU86_2 | 1, 2 | Martín-Torrijos et al., 2021 | MW327410 | MW325572 | Spain: San Zadornil (Burgos) |
|  | BU86_3 | 1, 2 | Martín-Torrijos et al., 2021 | MW327383 | MW325573 | Spain: San Zadornil (Burgos) |
|  | BU86_4 | 1, 2 | Martín-Torrijos et al., 2021 | MW327411 | MW325574 | Spain: San Zadornil (Burgos) |
|  | BU86_5 | 1, 2 | Martín-Torrijos et al., 2021 | MW327458 | MW325599 | Spain: San Zadornil (Burgos) |
| **BU98** | BU98_1 | 1, 2 | Matallanas et al., 2016 | EF489427 | FJ897843 | Spain: Padrones de Burela (Burgos) |
|  | BU98_2 | 1, 2 | Matallanas et al., 2016 | EF489427 | FJ897843 | Spain: Padrones de Burela (Burgos) |
|  | BU98_3 | 1, 2 | Matallanas et al., 2016 | EF489427 | FJ897843 | Spain: Padrones de Burela (Burgos) |
|  | BU98_4 | 1, 2 | Matallanas et al., 2016 | EF489427 | FJ897843 | Spain: Padrones de Burela (Burgos) |
|  | BU98_5 | 1, 2 | Matallanas et al., 2016 | EF489427 | FJ897843 | Spain: Padrones de Burela (Burgos) |
|  | BU98_6 | 1, 2 | Matallanas et al., 2016 | EF489427 | FJ897843 | Spain: Padrones de Burela (Burgos) |
|  | BU98_7 | 1, 2 | Matallanas et al., 2016 | EF489427 | FJ897843 | Spain: Padrones de Burela (Burgos) |
|  | BU98_8 | 1, 2 | Matallanas et al., 2016 | EF489427 | FJ897843 | Spain: Padrones de Burela (Burgos) |
|  | BU98_9 | 1, 2 | Matallanas et al., 2016 | EF489427 | FJ897843 | Spain: Padrones de Burela (Burgos) |
|  | BU98_10 | 1, 2 | Matallanas et al., 2016 | EF489427 | JF430570 | Spain: Padrones de Burela (Burgos) |
| **BU99** | BU99_1 | 1, 2 | Matallanas et al., 2016 | EF489427 | FJ897843 | Spain. Rebolledo Traspeña (Burgos) |
|  | BU99_2 | 1, 2 | Matallanas et al., 2016 | EF489427 | FJ897843 | Spain. Rebolledo Traspeña (Burgos) |
|  | BU99_3 | 1, 2 | Matallanas et al., 2016 | EF489427 | FJ897843 | Spain. Rebolledo Traspeña (Burgos) |
|  | BU99_4 | 1, 2 | Matallanas et al., 2016 | EF489427 | FJ897843 | Spain. Rebolledo Traspeña (Burgos) |
|  | BU99_5 | 1, 2 | Matallanas et al., 2016 | EF489427 | FJ897843 | Spain. Rebolledo Traspeña (Burgos) |
|  | BU99_6 | 1, 2 | Matallanas et al., 2016 | EF489427 | FJ897843 | Spain. Rebolledo Traspeña (Burgos) |
|  | BU99_7 | 1, 2 | Matallanas et al., 2016 | EF489427 | FJ897843 | Spain. Rebolledo Traspeña (Burgos) |
|  | BU99_8 | 1, 2 | Matallanas et al., 2016 | EF489427 | FJ897843 | Spain. Rebolledo Traspeña (Burgos) |
|  | BU99_9 | 1, 2 | Matallanas et al., 2016 | EF489427 | FJ897843 | Spain. Rebolledo Traspeña (Burgos) |
|  | BU99_10 | 1, 2 | Matallanas et al., 2016 | JF430581 | FJ897841 | Spain. Rebolledo Traspeña (Burgos) |
| **CAS1** | CAS1_1 | 1, 2 | Matallanas et al., 2016 | JF430574 | FJ897840 | Spain: Lucena del Cid (Castellón) |
|  | CAS1_2 | 1, 2 | Matallanas et al., 2016 | JF430574 | FJ897840 | Spain: Lucena del Cid (Castellón) |
|  | CAS1_3 | 1, 2 | Matallanas et al., 2016 | JF430574 | FJ897840 | Spain: Lucena del Cid (Castellón) |
|  | CAS1_4 | 1, 2 | Matallanas et al., 2016 | JF430574 | FJ897840 | Spain: Lucena del Cid (Castellón) |
|  | CAS1_5 | 1, 2 | Matallanas et al., 2016 | JF430574 | FJ897840 | Spain: Lucena del Cid (Castellón) |
|  | CAS1_6 | 1, 2 | Matallanas et al., 2016 | JF430574 | FJ897840 | Spain: Lucena del Cid (Castellón) |
|  | CAS1_7 | 1, 2 | Matallanas et al., 2016 | JF430574 | FJ897840 | Spain: Lucena del Cid (Castellón) |
|  | CAS1_8 | 1, 2 | Matallanas et al., 2016 | JF430574 | FJ897840 | Spain: Lucena del Cid (Castellón) |
|  | CAS1_9 | 1, 2 | Matallanas et al., 2016 | JF430574 | FJ897840 | Spain: Lucena del Cid (Castellón) |
|  | CAS1_10 | 1, 2 | Matallanas et al., 2016 | JF430574 | FJ897840 | Spain: Lucena del Cid (Castellón) |
| **CAS2** | CAS2_1 | 1, 2 | Matallanas et al., 2016 | JF430574 | FJ897840 | Spain: La Pobla de Benifassa (Castellón) |
|  | CAS2_2 | 1, 2 | Matallanas et al., 2016 | JF430574 | JF430573 | Spain: La Pobla de Benifassa (Castellón) |
|  | CAS2_3 | 1, 2 | Matallanas et al., 2016 | JF430574 | JF430573 | Spain: La Pobla de Benifassa (Castellón) |
|  | CAS2_4 | 1, 2 | Matallanas et al., 2016 | JF430574 | JF430573 | Spain: La Pobla de Benifassa (Castellón) |
|  | CAS2_5 | 1, 2 | Matallanas et al., 2016 | JF430574 | JF430573 | Spain: La Pobla de Benifassa (Castellón) |
|  | CAS2_6 | 1, 2 | Matallanas et al., 2016 | JF430574 | FJ897841 | Spain: La Pobla de Benifassa (Castellón) |
|  | CAS2_7 | 1, 2 | Matallanas et al., 2016 | JF430574 | FJ897841 | Spain: La Pobla de Benifassa (Castellón) |
|  | CAS2_8 | 1, 2 | Matallanas et al., 2016 | JF430574 | FJ897841 | Spain: La Pobla de Benifassa (Castellón) |
|  | CAS2_9 | 1, 2 | Matallanas et al., 2016 | JF430574 | FJ897841 | Spain: La Pobla de Benifassa (Castellón) |
|  | CAS2_10 | 1, 2 | Matallanas et al., 2016 | JF430574 | FJ897841 | Spain: La Pobla de Benifassa (Castellón) |
| **CAS3** | ID631 | 1 | Pedraza-Lara et al., 2010 | HM622595 | HM622601 | Spain: Zucaina |
|  | ID632 | 1 | Pedraza-Lara et al., 2010 | HM622595 | HM622601 | Spain: Zucaina |
|  | ID633 | 1 | Pedraza-Lara et al., 2010 | HM622595 | HM622601 | Spain: Zucaina |
| **CAS4** | ID637 | 1 | Pedraza-Lara et al., 2010 | HM622595 | HM622601 | Spain: Higueras |
|  | ID638 | 1 | Pedraza-Lara et al., 2010 | HM622595 | HM622601 | Spain: Higueras |
|  | ID639 | 1 | Pedraza-Lara et al., 2010 | HM622595 | HM622601 | Spain: Higueras |
| **COR1** | ID610 | 1 | Pedraza-Lara et al., 2010 | HM622595 | HM622601 | Spain: Cabra |
|  | ID611 | 1 | Pedraza-Lara et al., 2010 | HM622595 | HM622601 | Spain: Cabra |
|  | ID612 | 1 | Pedraza-Lara et al., 2010 | HM622595 | HM622601 | Spain: Cabra |
| **CR2** | CR2_2 | 1, 2 | Martín-Torrijos et al., 2021 | MW327204 | MW325493 | Sapin: Pozuelo de Calatrava (Ciudad Real) |
|  | CR2_3 | 1, 2 | Martín-Torrijos et al., 2021 | MW327277 | MW325452 | Sapin: Pozuelo de Calatrava (Ciudad Real) |
|  | CR2_4 | 1, 2 | Martín-Torrijos et al., 2021 | MW327205 | MW325494 | Sapin: Pozuelo de Calatrava (Ciudad Real) |
|  | CR2_5 | 1, 2 | Martín-Torrijos et al., 2021 | MW327206 | MW325400 | Sapin: Pozuelo de Calatrava (Ciudad Real) |
| **CRO1** | ID5 | 1 | Jelić et al., 2016 | KX370093 | KX369673 | Croatia: Arđila Creek |
|  | ID6 | 1 | Jelić et al., 2016 | KX370094 | KX369674 | Croatia: Arđila Creek |
|  | ID7 | 1 | Jelić et al., 2016 | KX370095 | KX369675 | Croatia: Arđila Creek |
|  | ID8 | 1 | Jelić et al., 2016 | KX370096 | KX369676 | Croatia: Arđila Creek |
| **CRO2** | ID9 | 1 | Jelić et al., 2016 | KX370097 | KX369677 | Croatia: Bračana River |
|  | ID10 | 1 | Jelić et al., 2016 | KX370098 | KX369678 | Croatia: Bračana River |
|  | ID11 | 1 | Jelić et al., 2016 | KX370099 | KX369679 | Croatia: Bračana River |
|  | ID12 | 1 | Jelić et al., 2016 | KX370100 | KX369680 | Croatia: Bračana River |
|  | ID13 | 1 | Jelić et al., 2016 | KX370101 | KX369681 | Croatia: Bračana River |
|  | ID14 | 1 | Jelić et al., 2016 | KX370102 | KX369682 | Croatia: Bračana River |
|  | ID15 | 1 | Jelić et al., 2016 | KX370103 | KX369683 | Croatia: Bračana River |
| **CRO3** | ID16 | 1 | Jelić et al., 2016 | KX370104 | KX369684 | Croatia: Pivka Creek |
|  | ID17 | 1 | Jelić et al., 2016 | KX370105 | KX369685 | Croatia: Pivka Creek |
| **CRO4** | ID19 | 1 | Jelić et al., 2016 | KX370107 | KX369687 | Croatia: Mudro Vrilo |
|  | ID20 | 1 | Jelić et al., 2016 | KX370108 | KX369688 | Croatia: Mudro Vrilo |
|  | ID21 | 1 | Jelić et al., 2016 | KX370109 | KX369689 | Croatia: Mudro Vrilo |
|  | ID22 | 1 | Jelić et al., 2016 | KX370110 | KX369690 | Croatia: Mudro Vrilo |
| **CRO5** | ID23 | 1 | Jelić et al., 2016 | KX370111 | KX369691 | Croatia: Krušnica River |
|  | ID24 | 1 | Jelić et al., 2016 | KX370112 | KX369692 | Croatia: Krušnica River |
|  | ID25 | 1 | Jelić et al., 2016 | KX370113 | KX369693 | Croatia: Krušnica River |
| **CRO6** | ID26 | 1 | Jelić et al., 2016 | KX370114 | KX369694 | Croatia: Otuča River |
|  | ID27 | 1 | Jelić et al., 2016 | KX370115 | KX369695 | Croatia: Otuča River |
| **CRO7** | ID28 | 1 | Jelić et al., 2016 | KX370116 | KX369696 | Croatia: Ruda River |
|  | ID29 | 1 | Jelić et al., 2016 | KX370117 | KX369697 | Croatia: Ruda River |
|  | ID18 | 1 | Jelić et al., 2016 | KX370106 | KX369686 | Croatia: Ruda River |
| **CRO8** | ID30 | 1 | Jelić et al., 2016 | KX370118 | KX369698 | Croatia: Konavočica River 1 |
|  | ID31 | 1 | Jelić et al., 2016 | KX370119 | KX369699 | Croatia: Konavočica River 1 |
|  | ID32 | 1 | Jelić et al., 2016 | KX370120 | KX369700 | Croatia: Konavočica River 1 |
|  | ID33 | 1 | Jelić et al., 2016 | KX370121 | KX369701 | Croatia: Konavočica River 1 |
|  | ID34 | 1 | Jelić et al., 2016 | KX370122 | KX369702 | Croatia: Konavočica River 1 |
| **CRO9** | ID35 | 1 | Jelić et al., 2016 | KX370123 | KX369703 | Croatia: Rječina River |
|  | ID36 | 1 | Jelić et al., 2016 | KX370124 | KX369704 | Croatia: Rječina River |
|  | ID37 | 1 | Jelić et al., 2016 | KX370125 | KX369705 | Croatia: Rječina River |
|  | ID38 | 1 | Jelić et al., 2016 | KX370126 | KX369706 | Croatia: Rječina River |
|  | ID39 | 1 | Jelić et al., 2016 | KX370127 | KX369707 | Croatia: Rječina River |
|  | ID40 | 1 | Jelić et al., 2016 | KX370128 | KX369708 | Croatia: Rečica Creek |
|  | ID41 | 1 | Jelić et al., 2016 | KX370129 | KX369709 | Croatia: Rečica Creek |
| **CRO10** | ID42 | 1 | Jelić et al., 2016 | KX370130 | KX369710 | Croatia: Mirna River, Kotli |
|  | ID43 | 1 | Jelić et al., 2016 | KX370131 | KX369711 | Croatia: Mirna River, Kotli |
|  | ID44 | 1 | Jelić et al., 2016 | KX370132 | KX369712 | Croatia: Mirna River, Kotli |
|  | ID45 | 1 | Jelić et al., 2016 | KX370133 | KX369713 | Croatia: Mirna River, Kotli |
| **CRO11** | ID46 | 1 | Jelić et al., 2016 | KX370134 | KX369714 | Croatia: Krupa River |
|  | ID47 | 1 | Jelić et al., 2016 | KX370135 | KX369715 | Croatia: Krupa River |
|  | ID48 | 1 | Jelić et al., 2016 | KX370136 | KX369716 | Croatia: Krupa River |
|  | ID49 | 1 | Jelić et al., 2016 | KX370137 | KX369717 | Croatia: Krupa River |
|  | ID50 | 1 | Jelić et al., 2016 | KX370138 | KX369718 | Croatia: Krupa River |
|  | ID51 | 1 | Jelić et al., 2016 | KX370139 | KX369719 | Croatia: Krupa River |
|  | ID52 | 1 | Jelić et al., 2016 | KX370140 | KX369720 | Croatia: Krupa River |
|  | ID53 | 1 | Jelić et al., 2016 | KX370141 | KX369721 | Croatia: Krupa River |
| **CRO12** | ID76 | 1 | Jelić et al., 2016 | KX370164 | KX369744 | Croatia: Neretva River, Bijeli Vir |
|  | ID77 | 1 | Jelić et al., 2016 | KX370165 | KX369745 | Croatia: Neretva River, Bijeli Vir |
|  | ID78 | 1 | Jelić et al., 2016 | KX370166 | KX369746 | Croatia: Neretva River, Bijeli Vir |
| **CRO13** | ID79 | 1 | Jelić et al., 2016 | KX370167 | KX369747 | Croatia: Raša River |
|  | ID80 | 1 | Jelić et al., 2016 | KX370168 | KX369748 | Croatia: Raša River |
|  | ID81 | 1 | Jelić et al., 2016 | KX370169 | KX369749 | Croatia: Raša River |
|  | ID82 | 1 | Jelić et al., 2016 | KX370170 | KX369750 | Croatia: Raša River |
| **CRO14** | ID83 | 1 | Jelić et al., 2016 | KX370171 | KX369751 | Croatia: Vransko Lake, Biograd |
|  | ID84 | 1 | Jelić et al., 2016 | KX370172 | KX369752 | Croatia: Vransko Lake, Biograd |
|  | ID85 | 1 | Jelić et al., 2016 | KX370173 | KX369753 | Croatia: Vransko Lake, Biograd |
|  | ID86 | 1 | Jelić et al., 2016 | KX370174 | KX369754 | Croatia: Vransko Lake, Biograd |
| **CRO17** | ID89 | 1 | Jelić et al., 2016 | KX370177 | KX369757 | Croatia: Zrmanja River |
|  | ID90 | 1 | Jelić et al., 2016 | KX370178 | KX369758 | Croatia: Zrmanja River |
| **CRO18** | ID91 | 1 | Jelić et al., 2016 | KX370179 | KX369759 | Croatia: Rakovac Creek |
|  | ID92 | 1 | Jelić et al., 2016 | KX370180 | KX369760 | Croatia: Rakovac Creek |
| **CRO19** | ID93 | 1 | Jelić et al., 2016 | KX370181 | KX369761 | Croatia: Bribirske Mostine |
|  | ID94 | 1 | Jelić et al., 2016 | KX370182 | KX369762 | Croatia: Bribirske Mostine |
| **CRO20** | ID95 | 1 | Jelić et al., 2016 | KX370183 | KX369763 | Croatia: Sutina Creek |
|  | ID96 | 1 | Jelić et al., 2016 | KX370184 | KX369764 | Croatia: Sutina Creek |
|  | ID97 | 1 | Jelić et al., 2016 | KX370185 | KX369765 | Croatia: Sutina Creek |
| **CRO21** | ID98 | 1 | Jelić et al., 2016 | KX370186 | KX369766 | Croatia: Butoniga Lake |
|  | ID99 | 1 | Jelić et al., 2016 | KX370187 | KX369767 | Croatia: Butoniga Lake |
|  | ID100 | 1 | Jelić et al., 2016 | KX370188 | KX369768 | Croatia: Butoniga Lake |
|  | ID88 | 1 | Jelić et al., 2016 | KX370176 | KX369756 | Croatia: Butoniga Lake |
| **CRO22** | ID101 | 1 | Jelić et al., 2016 | KX370189 | KX369769 | Croatia: Motovun |
|  | ID102 | 1 | Jelić et al., 2016 | KX370190 | KX369770 | Croatia: Motovun |
|  | ID129 | 1 | Jelić et al., 2016 | KX370217 | KX369797 | Croatia: Motovun |
| **CRO24** | ID113 | 1 | Jelić et al., 2016 | KX370201 | KX369781 | Croatia: Badnjevica Canyon |
|  | ID114 | 1 | Jelić et al., 2016 | KX370202 | KX369782 | Croatia: Badnjevica Canyon |
|  | ID115 | 1 | Jelić et al., 2016 | KX370203 | KX369783 | Croatia: Badnjevica Canyon |
|  | ID116 | 1 | Jelić et al., 2016 | KX370204 | KX369784 | Croatia: Badnjevica Canyon |
|  | ID117 | 1 | Jelić et al., 2016 | KX370205 | KX369785 | Croatia: Badnjevica Canyon |
|  | ID103 | 1 | Jelić et al., 2016 | KX370191 | KX369771 | Croatia: Badnjevica Canyon |
|  | ID87 | 1 | Jelić et al., 2016 | KX370175 | KX369755 | Croatia: Badnjevica Canyon |
| **CRO25** | ID127 | 1 | Jelić et al., 2016 | KX370215 | KX369795 | Croatia: Vransko Lake, Cres Island |
|  | ID128 | 1 | Jelić et al., 2016 | KX370216 | KX369796 | Croatia: Vransko Lake, Cres Island |
| **CU1** | CU1_3 | 1, 2 | Martín-Torrijos et al., 2021 | MW327207 | MW325398 | Spain: Almagro (Cuenca) |
|  | CU1_4 | 1, 2 | Martín-Torrijos et al., 2021 | MW327295 | MW325474 | Spain: Almagro (Cuenca) |
| **CU2** | CU2_1 | 1, 2 | Martín-Torrijos et al., 2021 | MW327278 | MW325492 | Spain: Las Truchas (Cuenca) |
|  | CU2_2 | 1, 2 | Martín-Torrijos et al., 2021 | MW327209 | MW325491 | Spain: Las Truchas (Cuenca) |
|  | CU2_3 | 1, 2 | Martín-Torrijos et al., 2021 | MW327208 | MW325490 | Spain: Las Truchas (Cuenca) |
| **CU3** | CU3_1 | 1, 2 | Martín-Torrijos et al., 2021 | MW327210 | MW325504 | Spain: Pedregosos (Cuenca) |
|  | CU3_2 | 1, 2 | Martín-Torrijos et al., 2021 | MW327211 | MW325505 | Spain: Pedregosos (Cuenca) |
|  | CU3_3 | 1, 2 | Martín-Torrijos et al., 2021 | MW327212 | MW325531 | Spain: Pedregosos (Cuenca) |
| **CU4** | CU4_2 | 1, 2 | Martín-Torrijos et al., 2021 | MW327424 | MW325532 | Spain: Pozuelo (Cuenca) |
|  | CU4_3 | 1, 2 | Martín-Torrijos et al., 2021 | MW327213 | MW325355 | Spain: Pozuelo (Cuenca) |
|  | CU4_4 | 1, 2 | Martín-Torrijos et al., 2021 | MW327214 | MW325468 | Spain: Pozuelo (Cuenca) |
| **CU5** | CU5_2 | 1, 2 | Martín-Torrijos et al., 2021 | MW327232 | MW325486 | Spain: Valmelero (Cuenca) |
|  | CU5_3 | 1, 2 | Martín-Torrijos et al., 2021 | MW327233 | MW325487 | Spain: Valmelero (Cuenca) |
|  | CU5_4 | 1, 2 | Martín-Torrijos et al., 2021 | MW327234 | MW325488 | Spain: Valmelero (Cuenca) |
|  | CU5_5 | 1, 2 | Martín-Torrijos et al., 2021 | MW327235 | MW325489 | Spain: Valmelero (Cuenca) |
| **CU6** | CU6_1 | 1, 2 | Martín-Torrijos et al., 2021 | MW327250 | MW325483 | Spain: Vaquerizas (Cuenca) |
|  | CU6_2 | 1, 2 | Martín-Torrijos et al., 2021 | MW327251 | MW325484 | Spain: Vaquerizas (Cuenca) |
|  | CU6_4 | 1, 2 | Martín-Torrijos et al., 2021 | MW327289 | MW325485 | Spain: Vaquerizas (Cuenca) |
| **CU7** | CU7_1 | 1, 2 | Matallanas et al., 2016 | JF430574 | FJ897841 | Spain: Huerta de Obispalia (Cuenca) |
|  | CU7_2 | 1, 2 | Matallanas et al., 2016 | JF430574 | FJ897841 | Spain: Huerta de Obispalia (Cuenca) |
|  | CU7_3 | 1, 2 | Matallanas et al., 2016 | JF430574 | FJ897841 | Spain: Huerta de Obispalia (Cuenca) |
|  | CU7_4 | 1, 2 | Matallanas et al., 2016 | JF430574 | FJ897841 | Spain: Huerta de Obispalia (Cuenca) |
|  | CU7_5 | 1, 2 | Matallanas et al., 2016 | JF430574 | FJ897841 | Spain: Huerta de Obispalia (Cuenca) |
|  | CU7_6 | 1, 2 | Matallanas et al., 2016 | JF430574 | FJ897841 | Spain: Huerta de Obispalia (Cuenca) |
|  | CU7_7 | 1, 2 | Matallanas et al., 2016 | JF430574 | FJ897841 | Spain: Huerta de Obispalia (Cuenca) |
|  | CU7_8 | 1, 2 | Matallanas et al., 2016 | JF430574 | FJ897841 | Spain: Huerta de Obispalia (Cuenca) |
|  | CU7_9 | 1, 2 | Matallanas et al., 2016 | JF430574 | FJ897841 | Spain: Huerta de Obispalia (Cuenca) |
|  | CU7_10 | 1, 2 | Matallanas et al., 2016 | JF430574 | JF430568 | Spain: Huerta de Obispalia (Cuenca) |
| **CU8** | CU8_1 | 1, 2 | Matallanas et al., 2016 | JF430574 | FJ897841 | Spain: Pozuelo (Cuenca) |
|  | CU8_2 | 1, 2 | Matallanas et al., 2016 | JF430574 | FJ897841 | Spain: Pozuelo (Cuenca) |
|  | CU8_3 | 1, 2 | Matallanas et al., 2016 | JF430574 | FJ897841 | Spain: Pozuelo (Cuenca) |
|  | CU8_4 | 1, 2 | Matallanas et al., 2016 | JF430574 | FJ897841 | Spain: Pozuelo (Cuenca) |
|  | CU8_5 | 1, 2 | Matallanas et al., 2016 | JF430574 | FJ897841 | Spain: Pozuelo (Cuenca) |
|  | CU8_6 | 1, 2 | Matallanas et al., 2016 | JF430574 | FJ897841 | Spain: Pozuelo (Cuenca) |
|  | CU8_7 | 1, 2 | Matallanas et al., 2016 | JF430574 | FJ897841 | Spain: Pozuelo (Cuenca) |
|  | CU8_8 | 1, 2 | Matallanas et al., 2016 | JF430574 | FJ897841 | Spain: Pozuelo (Cuenca) |
|  | CU8_9 | 1, 2 | Matallanas et al., 2016 | JF430574 | FJ897841 | Spain: Pozuelo (Cuenca) |
|  | CU8_10 | 1, 2 | Matallanas et al., 2016 | JF430574 | FJ897841 | Spain: Pozuelo (Cuenca) |
| **CU9** | CU9_1 | 1, 2 | Matallanas et al., 2016 | JF430574 | FJ897840 | Spain: Valdemoro (Cuenca) |
|  | CU9_2 | 1, 2 | Matallanas et al., 2016 | JF430574 | FJ897840 | Spain: Valdemoro (Cuenca) |
|  | CU9_3 | 1, 2 | Matallanas et al., 2016 | JF430574 | FJ897840 | Spain: Valdemoro (Cuenca) |
|  | CU9_4 | 1, 2 | Matallanas et al., 2016 | JF430578 | FJ897840 | Spain: Valdemoro (Cuenca) |
|  | CU9_5 | 1, 2 | Matallanas et al., 2016 | JF430574 | FJ897841 | Spain: Valdemoro (Cuenca) |
|  | CU9_6 | 1, 2 | Matallanas et al., 2016 | JF430574 | FJ897841 | Spain: Valdemoro (Cuenca) |
|  | CU9_7 | 1, 2 | Matallanas et al., 2016 | JF430574 | FJ897841 | Spain: Valdemoro (Cuenca) |
|  | CU9_8 | 1, 2 | Matallanas et al., 2016 | JF430574 | FJ897841 | Spain: Valdemoro (Cuenca) |
|  | CU9_9 | 1, 2 | Matallanas et al., 2016 | JF430574 | FJ897841 | Spain: Valdemoro (Cuenca) |
|  | CU9_10 | 1, 2 | Matallanas et al., 2016 | JF430574 | FJ897841 | Spain: Valdemoro (Cuenca) |
| **FRA1** | ID130 | 1 | Jelić et al., 2016 | KX370218 | KX369798 | France: Roya River |
|  | ID131 | 1 | Jelić et al., 2016 | KX370219 | KX369799 | France: Roya River |
|  | ID132 | 1 | Jelić et al., 2016 | KX370220 | KX369800 | France: Roya River |
|  | ID133 | 1 | Jelić et al., 2016 | KX370221 | KX369801 | France: Roya River |
|  | ID134 | 1 | Jelić et al., 2016 | KX370222 | KX369802 | France: Roya River |
|  | ID135 | 1 | Jelić et al., 2016 | KX370223 | KX369803 | France: Roya River |
|  | ID136 | 1 | Jelić et al., 2016 | KX370224 | KX369804 | France: Roya River |
|  | ID137 | 1 | Jelić et al., 2016 | KX370225 | KX369805 | France: Roya River |
|  | ID138 | 1 | Jelić et al., 2016 | KX370226 | KX369806 | France: Roya River |
|  | ID139 | 1 | Jelić et al., 2016 | KX370227 | KX369807 | France: Roya River |
|  | ID140 | 1 | Jelić et al., 2016 | KX370228 | KX369808 | France: Roya River |
|  | ID141 | 1 | Jelić et al., 2016 | KX370229 | KX369809 | France: Roya River |
|  | ID142 | 1 | Jelić et al., 2016 | KX370230 | KX369810 | France: Roya River |
|  | ID143 | 1 | Jelić et al., 2016 | KX370231 | KX369811 | France: Roya River |
| **FRA2** | ID149 | 1 | Jelić et al., 2016 | KX370237 | KX369817 | France: Le Garrel |
|  | ID150 | 1 | Jelić et al., 2016 | KX370238 | KX369818 | France: Le Garrel |
|  | ID151 | 1 | Jelić et al., 2016 | KX370239 | KX369819 | France: Le Garrel |
|  | ID152 | 1 | Jelić et al., 2016 | KX370240 | KX369820 | France: Le Garrel |
| **FRA4** | ID156 | 1 | Jelić et al., 2016 | KX370244 | KX369824 | France: Val Renard |
|  | ID157 | 1 | Jelić et al., 2016 | KX370245 | KX369825 | France: Val Renard |
|  | ID158 | 1 | Jelić et al., 2016 | KX370246 | KX369826 | France: Val Renard |
|  | ID159 | 1 | Jelić et al., 2016 | KX370247 | KX369827 | France: Val Renard |
|  | ID160 | 1 | Jelić et al., 2016 | KX370248 | KX369828 | France: Val Renard |
|  | ID161 | 1 | Jelić et al., 2016 | KX370249 | KX369829 | France: Val Renard |
|  | ID162 | 1 | Jelić et al., 2016 | KX370250 | KX369830 | France: Val Renard |
| **FRA5** | ID206 | 1 | Jelić et al., 2016 | KX370294 | KX369874 | France: Alagnon |
|  | ID207 | 1 | Jelić et al., 2016 | KX370295 | KX369875 | France: Alagnon |
|  | ID208 | 1 | Jelić et al., 2016 | KX370296 | KX369876 | France: Alagnon |
|  | ID209 | 1 | Jelić et al., 2016 | KX370297 | KX369877 | France: Alagnon |
|  | ID210 | 1 | Jelić et al., 2016 | KX370298 | KX369878 | France: Alagnon |
|  | ID211 | 1 | Jelić et al., 2016 | KX370299 | KX369879 | France: Alagnon |
|  | ID212 | 1 | Jelić et al., 2016 | KX370300 | KX369880 | France: Alagnon |
|  | ID213 | 1 | Jelić et al., 2016 | KX370301 | KX369881 | France: Alagnon |
| **VIZ1** | ID218 | 1 | Jelić et al., 2016 | KX370306 | KX369886 | Spain: Bilbao |
|  | ID219 | 1 | Jelić et al., 2016 | KX370307 | KX369887 | Spain: Bilbao |
|  | ID220 | 1 | Jelić et al., 2016 | KX370308 | KX369888 | Spain: Bilbao |
|  | ID221 | 1 | Jelić et al., 2016 | KX370309 | KX369889 | Spain: Bilbao |
|  | ID222 | 1 | Jelić et al., 2016 | KX370310 | KX369890 | Spain: Bilbao |
|  | ID223 | 1 | Jelić et al., 2016 | KX370311 | KX369891 | Spain: Bilbao |
|  | ID224 | 1 | Jelić et al., 2016 | KX370312 | KX369892 | Spain: Bilbao |
|  | ID225 | 1 | Jelić et al., 2016 | KX370313 | KX369893 | Spain: Bilbao |
|  | ID226 | 1 | Jelić et al., 2016 | KX370314 | KX369894 | Spain: Bilbao |
|  | ID227 | 1 | Jelić et al., 2016 | KX370315 | KX369895 | Spain: Bilbao |
|  | ID228 | 1 | Jelić et al., 2016 | KX370316 | KX369896 | Spain: Bilbao |
| **FRA6** | ID246 | 1 | Jelić et al., 2016 | KX370334 | KX369914 | France: Boussuivre |
|  | ID247 | 1 | Jelić et al., 2016 | KX370335 | KX369915 | France: Boussuivre |
|  | ID248 | 1 | Jelić et al., 2016 | KX370336 | KX369916 | France: Boussuivre |
|  | ID249 | 1 | Jelić et al., 2016 | KX370337 | KX369917 | France: Boussuivre |
|  | ID250 | 1 | Jelić et al., 2016 | KX370338 | KX369918 | France: Boussuivre |
|  | ID251 | 1 | Jelić et al., 2016 | KX370339 | KX369919 | France: Boussuivre |
|  | ID252 | 1 | Jelić et al., 2016 | KX370340 | KX369920 | France: Boussuivre |
|  | ID253 | 1 | Jelić et al., 2016 | KX370341 | KX369921 | France: Boussuivre |
|  | ID254 | 1 | Jelić et al., 2016 | KX370342 | KX369922 | France: Boussuivre |
| **FRA7** | ID255 | 1 | Jelić et al., 2016 | KX370343 | KX369923 | France: Casabianca, Corsica Island |
|  | ID256 | 1 | Jelić et al., 2016 | KX370344 | KX369924 | France: Casabianca, Corsica Island |
|  | ID257 | 1 | Jelić et al., 2016 | KX370345 | KX369925 | France: Casabianca, Corsica Island |
|  | ID258 | 1 | Jelić et al., 2016 | KX370346 | KX369926 | France: Casabianca, Corsica Island |
|  | ID259 | 1 | Jelić et al., 2016 | KX370347 | KX369927 | France: Casabianca, Corsica Island |
|  | ID260 | 1 | Jelić et al., 2016 | KX370348 | KX369928 | France: Casabianca, Corsica Island |
|  | ID261 | 1 | Jelić et al., 2016 | KX370349 | KX369929 | France: Casabianca, Corsica Island |
|  | ID262 | 1 | Jelić et al., 2016 | KX370350 | KX369930 | France: Casabianca, Corsica Island |
| **FRA8** | ID263 | 1 | Jelić et al., 2016 | KX370351 | KX369931 | France: Fiume, Corsica Island |
|  | ID264 | 1 | Jelić et al., 2016 | KX370352 | KX369932 | France: Fiume, Corsica Island |
|  | ID265 | 1 | Jelić et al., 2016 | KX370353 | KX369933 | France: Fiume, Corsica Island |
|  | ID266 | 1 | Jelić et al., 2016 | KX370354 | KX369934 | France: Fiume, Corsica Island |
|  | ID267 | 1 | Jelić et al., 2016 | KX370355 | KX369935 | France: Fiume, Corsica Island |
|  | ID268 | 1 | Jelić et al., 2016 | KX370356 | KX369936 | France: Fiume, Corsica Island |
|  | ID269 | 1 | Jelić et al., 2016 | KX370357 | KX369937 | France: Fiume, Corsica Island |
|  | ID270 | 1 | Jelić et al., 2016 | KX370358 | KX369938 | France: Fiume, Corsica Island |
|  | ID271 | 1 | Jelić et al., 2016 | KX370359 | KX369939 | France: Fiume, Corsica Island |
|  | ID272 | 1 | Jelić et al., 2016 | KX370360 | KX369940 | France: Fiume, Corsica Island |
|  | ID273 | 1 | Jelić et al., 2016 | KX370361 | KX369941 | France: Fiume, Corsica Island |
|  | ID274 | 1 | Jelić et al., 2016 | KX370362 | KX369942 | France: Fiume, Corsica Island |
|  | ID275 | 1 | Jelić et al., 2016 | KX370363 | KX369943 | France: Fiume, Corsica Island |
|  | ID276 | 1 | Jelić et al., 2016 | KX370364 | KX369944 | France: Fiume, Corsica Island |
| **FRA9** | ID277 | 1 | Jelić et al., 2016 | KX370365 | KX369945 | France: Crochatiere |
|  | ID278 | 1 | Jelić et al., 2016 | KX370366 | KX369946 | France: Crochatiere |
|  | ID279 | 1 | Jelić et al., 2016 | KX370367 | KX369947 | France: Crochatiere |
|  | ID280 | 1 | Jelić et al., 2016 | KX370368 | KX369948 | France: Crochatiere |
|  | ID281 | 1 | Jelić et al., 2016 | KX370369 | KX369949 | France: Crochatiere |
|  | ID282 | 1 | Jelić et al., 2016 | KX370370 | KX369950 | France: Crochatiere |
|  | ID283 | 1 | Jelić et al., 2016 | KX370371 | KX369951 | France: Crochatiere |
|  | ID284 | 1 | Jelić et al., 2016 | KX370372 | KX369952 | France: Crochatiere |
|  | ID285 | 1 | Jelić et al., 2016 | KX370373 | KX369953 | France: Crochatiere |
|  | ID286 | 1 | Jelić et al., 2016 | KX370374 | KX369954 | France: Crochatiere |
|  | ID287 | 1 | Jelić et al., 2016 | KX370375 | KX369955 | France: Crochatiere |
| **FRA10** | ID310 | 1 | Jelić et al., 2016 | KX370398 | KX369978 | France: Gand River |
|  | ID311 | 1 | Jelić et al., 2016 | KX370399 | KX369979 | France: Gand River |
|  | ID312 | 1 | Jelić et al., 2016 | KX370400 | KX369980 | France: Gand River |
|  | ID313 | 1 | Jelić et al., 2016 | KX370401 | KX369981 | France: Gand River |
|  | ID314 | 1 | Jelić et al., 2016 | KX370402 | KX369982 | France: Gand River |
|  | ID315 | 1 | Jelić et al., 2016 | KX370403 | KX369983 | France: Gand River |
|  | ID316 | 1 | Jelić et al., 2016 | KX370404 | KX369984 | France: Gand River |
|  | ID317 | 1 | Jelić et al., 2016 | KX370405 | KX369985 | France: Gand River |
|  | ID318 | 1 | Jelić et al., 2016 | KX370406 | KX369986 | France: Gand River |
|  | ID319 | 1 | Jelić et al., 2016 | KX370407 | KX369987 | France: Gand River |
|  | ID320 | 1 | Jelić et al., 2016 | KX370408 | KX369988 | France: Gand River |
| **FRA11** | ID321 | 1 | Jelić et al., 2016 | KX370409 | KX369989 | France: Gatineau |
|  | ID322 | 1 | Jelić et al., 2016 | KX370410 | KX369990 | France: Gatineau |
|  | ID323 | 1 | Jelić et al., 2016 | KX370411 | KX369991 | France: Gatineau |
|  | ID324 | 1 | Jelić et al., 2016 | KX370412 | KX369992 | France: Gatineau |
|  | ID325 | 1 | Jelić et al., 2016 | KX370413 | KX369993 | France: Gatineau |
|  | ID326 | 1 | Jelić et al., 2016 | KX370414 | KX369994 | France: Gatineau |
|  | ID327 | 1 | Jelić et al., 2016 | KX370415 | KX369995 | France: Gatineau |
|  | ID328 | 1 | Jelić et al., 2016 | KX370416 | KX369996 | France: Gatineau |
| **FRA13** | ID342 | 1 | Jelić et al., 2016 | KX370430 | KX370010 | France: Haute Saone region |
|  | ID343 | 1 | Jelić et al., 2016 | KX370431 | KX370011 | France: Haute Saone region |
|  | ID344 | 1 | Jelić et al., 2016 | KX370432 | KX370012 | France: Haute Saone region |
|  | ID345 | 1 | Jelić et al., 2016 | KX370433 | KX370013 | France: Haute Saone region |
|  | ID346 | 1 | Jelić et al., 2016 | KX370434 | KX370014 | France: Haute Saone region |
|  | ID347 | 1 | Jelić et al., 2016 | KX370435 | KX370015 | France: Haute Saone region |
|  | ID348 | 1 | Jelić et al., 2016 | KX370436 | KX370016 | France: Haute Saone region |
|  | ID349 | 1 | Jelić et al., 2016 | KX370437 | KX370017 | France: Haute Saone region |
|  | ID350 | 1 | Jelić et al., 2016 | KX370438 | KX370018 | France: Haute Saone region |
|  | ID351 | 1 | Jelić et al., 2016 | KX370439 | KX370019 | France: Haute Saone region |
|  | ID352 | 1 | Jelić et al., 2016 | KX370440 | KX370020 | France: Haute Saone region |
|  | ID353 | 1 | Jelić et al., 2016 | KX370441 | KX370021 | France: Haute Saone region |
|  | ID354 | 1 | Jelić et al., 2016 | KX370442 | KX370022 | France: Haute Saone region |
| **FRA14** | ID355 | 1 | Jelić et al., 2016 | KX370443 | KX370023 | France: Isere |
|  | ID356 | 1 | Jelić et al., 2016 | KX370444 | KX370024 | France: Isere |
|  | ID357 | 1 | Jelić et al., 2016 | KX370445 | KX370025 | France: Isere |
|  | ID358 | 1 | Jelić et al., 2016 | KX370446 | KX370026 | France: Isere |
|  | ID359 | 1 | Jelić et al., 2016 | KX370447 | KX370027 | France: Isere |
|  | ID360 | 1 | Jelić et al., 2016 | KX370448 | KX370028 | France: Isere |
|  | ID361 | 1 | Jelić et al., 2016 | KX370449 | KX370029 | France: Isere |
|  | ID362 | 1 | Jelić et al., 2016 | KX370450 | KX370030 | France: Isere |
|  | ID363 | 1 | Jelić et al., 2016 | KX370451 | KX370031 | France: Isere |
|  | ID364 | 1 | Jelić et al., 2016 | KX370452 | KX370032 | France: Isere |
|  | ID365 | 1 | Jelić et al., 2016 | KX370453 | KX370033 | France: Isere |
|  | ID366 | 1 | Jelić et al., 2016 | KX370454 | KX370034 | France: Isere |
|  | ID367 | 1 | Jelić et al., 2016 | KX370455 | KX370035 | France: Isere |
|  | ID368 | 1 | Jelić et al., 2016 | KX370456 | KX370036 | France: Isere |
| **FRA15** | ID369 | 1 | Jelić et al., 2016 | KX370457 | KX370037 | France: Las Illas |
|  | ID370 | 1 | Jelić et al., 2016 | KX370458 | KX370038 | France: Las Illas |
|  | ID371 | 1 | Jelić et al., 2016 | KX370459 | KX370039 | France: Las Illas |
|  | ID372 | 1 | Jelić et al., 2016 | KX370460 | KX370040 | France: Las Illas |
|  | ID373 | 1 | Jelić et al., 2016 | KX370461 | KX370041 | France: Las Illas |
|  | ID374 | 1 | Jelić et al., 2016 | KX370462 | KX370042 | France: Las Illas |
|  | ID375 | 1 | Jelić et al., 2016 | KX370463 | KX370043 | France: Las Illas |
|  | ID376 | 1 | Jelić et al., 2016 | KX370464 | KX370044 | France: Las Illas |
| **FRA16** | ID398 | 1 | Jelić et al., 2016 | KX370486 | KX370066 | France: Puit d Enfer |
|  | ID399 | 1 | Jelić et al., 2016 | KX370487 | KX370067 | France: Puit d Enfer |
|  | ID400 | 1 | Jelić et al., 2016 | KX370488 | KX370068 | France: Puit d Enfer |
|  | ID401 | 1 | Jelić et al., 2016 | KX370489 | KX370069 | France: Puit d Enfer |
|  | ID402 | 1 | Jelić et al., 2016 | KX370490 | KX370070 | France: Puit d Enfer |
|  | ID403 | 1 | Jelić et al., 2016 | KX370491 | KX370071 | France: Puit d Enfer |
|  | ID404 | 1 | Jelić et al., 2016 | KX370492 | KX370072 | France: Puit d Enfer |
|  | ID405 | 1 | Jelić et al., 2016 | KX370493 | KX370073 | France: Puit d Enfer |
|  | ID406 | 1 | Jelić et al., 2016 | KX370494 | KX370074 | France: Puit d Enfer |
| **FRA17** | ID407 | 1 | Jelić et al., 2016 | KX370495 | KX370075 | France: Tallard |
|  | ID408 | 1 | Jelić et al., 2016 | KX370496 | KX370076 | France: Tallard |
|  | ID409 | 1 | Jelić et al., 2016 | KX370497 | KX370077 | France: Tallard |
| **FRA18** | ID410 | 1 | Jelić et al., 2016 | KX370498 | KX370078 | France: Vonne |
|  | ID411 | 1 | Jelić et al., 2016 | KX370499 | KX370079 | France: Vonne |
|  | ID412 | 1 | Jelić et al., 2016 | KX370500 | KX370080 | France: Vonne |
|  | ID413 | 1 | Jelić et al., 2016 | KX370501 | KX370081 | France: Vonne |
|  | ID414 | 1 | Jelić et al., 2016 | KX370502 | KX370082 | France: Vonne |
|  | ID415 | 1 | Jelić et al., 2016 | KX370503 | KX370083 | France: Vonne |
|  | ID416 | 1 | Jelić et al., 2016 | KX370504 | KX370084 | France: Vonne |
| **GB1** | ID144 | 1 | Jelić et al., 2016 | KX370232 | KX369812 | Great Britain: Avon |
|  | ID145 | 1 | Jelić et al., 2016 | KX370233 | KX369813 | Great Britain: Avon |
|  | ID146 | 1 | Jelić et al., 2016 | KX370234 | KX369814 | Great Britain: Avon |
| **GIR1** | GIR1_1 | 1, 2 | Martín-Torrijos et al., 2021 | MW327380 | MW325353 | Spain: Escaramat (Girona) |
|  | GIR1_2 | 1, 2 | Martín-Torrijos et al., 2021 | MW327379 | MW325354 | Spain: Escaramat (Girona) |
|  | GIR1_3 | 1, 2 | Martín-Torrijos et al., 2021 | MW327312 | MW325495 | Spain: Escaramat (Girona) |
| **GIR2** | GIR2_1 | 1, 2 | Martín-Torrijos et al., 2021 | MW327236 | MW325466 | Spain: Falgars (Girona) |
|  | GIR2_11 | 1, 2 | Martín-Torrijos et al., 2021 | MW327304 | MW325464 | Spain: Falgars (Girona) |
|  | GIR2_12 | 1, 2 | Martín-Torrijos et al., 2021 | MW327339 | MW325460 | Spain: Falgars (Girona) |
|  | GIR2_2 | 1, 2 | Martín-Torrijos et al., 2021 | MW327291 | MW325458 | Spain: Falgars (Girona) |
|  | GIR2_3 | 1, 2 | Martín-Torrijos et al., 2021 | MW327337 | MW325459 | Spain: Falgars (Girona) |
|  | GIR2_4 | 1, 2 | Martín-Torrijos et al., 2021 | MW327237 | MW325461 | Spain: Falgars (Girona) |
|  | GIR2_5 | 1, 2 | Martín-Torrijos et al., 2021 | MW327238 | MW325410 | Spain: Falgars (Girona) |
|  | GIR2_6 | 1, 2 | Martín-Torrijos et al., 2021 | MW327240 | MW325462 | Spain: Falgars (Girona) |
|  | GIR2_7 | 1, 2 | Martín-Torrijos et al., 2021 | MW327296 | MW325465 | Spain: Falgars (Girona) |
|  | GIR2_8 | 1, 2 | Martín-Torrijos et al., 2021 | MW327239 | MW325411 | Spain: Falgars (Girona) |
|  | GIR2_9 | 1, 2 | Martín-Torrijos et al., 2021 | MW327338 | MW325463 | Spain: Falgars (Girona) |
| **GIR3** | GIR3_1 | 1, 2 | Martín-Torrijos et al., 2021 | MW327281 | MW325470 | Spain: La Fabrega (Girona) |
|  | GIR3_10 | 1, 2 | Martín-Torrijos et al., 2021 | MW327307 | MW325438 | Spain: La Fabrega (Girona) |
|  | GIR3_11 | 1, 2 | Martín-Torrijos et al., 2021 | MW327359 | MW325356 | Spain: La Fabrega (Girona) |
|  | GIR3_12 | 1, 2 | Martín-Torrijos et al., 2021 | MW327360 | MW325357 | Spain: La Fabrega (Girona) |
|  | GIR3_13 | 1, 2 | Martín-Torrijos et al., 2021 | MW327361 | MW325358 | Spain: La Fabrega (Girona) |
|  | GIR3_2 | 1, 2 | Martín-Torrijos et al., 2021 | MW327241 | MW325394 | Spain: La Fabrega (Girona) |
|  | GIR3_4 | 1, 2 | Martín-Torrijos et al., 2021 | MW327242 | MW325395 | Spain: La Fabrega (Girona) |
|  | GIR3_5 | 1, 2 | Martín-Torrijos et al., 2021 | MW327282 | MW325471 | Spain: La Fabrega (Girona) |
|  | GIR3_6 | 1, 2 | Martín-Torrijos et al., 2021 | MW327283 | MW325455 | Spain: La Fabrega (Girona) |
|  | GIR3_7 | 1, 2 | Martín-Torrijos et al., 2021 | MW327305 | MW325529 | Spain: La Fabrega (Girona) |
|  | GIR3_8 | 1, 2 | Martín-Torrijos et al., 2021 | MW327243 | MW325396 | Spain: La Fabrega (Girona) |
|  | GIR3_9 | 1, 2 | Martín-Torrijos et al., 2021 | MW327244 | MW325472 | Spain: La Fabrega (Girona) |
| **GIR4** | GIR4_1 | 1, 2 | Martín-Torrijos et al., 2021 | MW327313 | MW325450 | Spain: La Plana (Girona) |
|  | GIR4_10 | 1, 2 | Martín-Torrijos et al., 2021 | MW327254 | MW325443 | Spain: La Plana (Girona) |
|  | GIR4_11 | 1, 2 | Martín-Torrijos et al., 2021 | MW327288 | MW325444 | Spain: La Plana (Girona) |
|  | GIR4_12 | 1, 2 | Martín-Torrijos et al., 2021 | MW327306 | MW325446 | Spain: La Plana (Girona) |
|  | GIR4_2 | 1, 2 | Martín-Torrijos et al., 2021 | MW327279 | MW325441 | Spain: La Plana (Girona) |
|  | GIR4_3 | 1, 2 | Martín-Torrijos et al., 2021 | MW327252 | MW325442 | Spain: La Plana (Girona) |
|  | GIR4_4 | 1, 2 | Martín-Torrijos et al., 2021 | MW327368 | MW325528 | Spain: La Plana (Girona) |
|  | GIR4_5 | 1, 2 | Martín-Torrijos et al., 2021 | MW327317 | MW325447 | Spain: La Plana (Girona) |
|  | GIR4_6 | 1, 2 | Martín-Torrijos et al., 2021 | MW327280 | MW325412 | Spain: La Plana (Girona) |
|  | GIR4_7 | 1, 2 | Martín-Torrijos et al., 2021 | MW327253 | MW325445 | Spain: La Plana (Girona) |
|  | GIR4_9 | 1, 2 | Martín-Torrijos et al., 2021 | MW327298 | MW325467 | Spain: La Plana (Girona) |
| **GIR5** | GIR5_1 | - | In this study | - | - | Spain: La Muga (Girona) |
|  | GIR5_2 | - | In this study | - | - | Spain: La Muga (Girona) |
|  | GIR5_3 | - | In this study | - | - | Spain: La Muga (Girona) |
|  | GIR5_4 | - | In this study | - | - | Spain: La Muga (Girona) |
|  | GIR5_5 | - | In this study | - | - | Spain: La Muga (Girona) |
| **GIR6** | GIR6_1 | 1, 2 | Martín-Torrijos et al., 2021 | MW327301 | MW325530 | Spain: Santa Llucía (Girona) |
|  | GIR6_10 | 1, 2 | Martín-Torrijos et al., 2021 | MW327269 | MW325501 | Spain: Santa Llucía (Girona) |
|  | GIR6_11 | 1, 2 | Martín-Torrijos et al., 2021 | MW327316 | MW325502 | Spain: Santa Llucía (Girona) |
|  | GIR6_12 | 1, 2 | Martín-Torrijos et al., 2021 | MW327271 | MW325503 | Spain: Santa Llucía (Girona) |
|  | GIR6_2 | 1, 2 | Martín-Torrijos et al., 2021 | MW327266 | MW325456 | Spain: Santa Llucía (Girona) |
|  | GIR6_3 | 1, 2 | Martín-Torrijos et al., 2021 | MW327303 | MW325526 | Spain: Santa Llucía (Girona) |
|  | GIR6_5 | 1, 2 | Martín-Torrijos et al., 2021 | MW327267 | MW325527 | Spain: Santa Llucía (Girona) |
|  | GIR6_6 | 1, 2 | Martín-Torrijos et al., 2021 | MW327285 | MW325523 | Spain: Santa Llucía (Girona) |
|  | GIR6_7 | 1, 2 | Martín-Torrijos et al., 2021 | MW327268 | MW325401 | Spain: Santa Llucía (Girona) |
|  | GIR6_8 | 1, 2 | Martín-Torrijos et al., 2021 | MW327302 | MW325402 | Spain: Santa Llucía (Girona) |
|  | GIR6_9 | 1, 2 | Martín-Torrijos et al., 2021 | MW327270 | MW325457 | Spain: Santa Llucía (Girona) |
| **GIR7** | GIR7_1 | 1, 2 | In this study | OR195158 | OR197367 | Spain: Olot (Girona) |
|  | GIR7_2 | 1, 2 | In this study | OR195159 | OR197368 | Spain: Olot (Girona) |
|  | GIR7_3 | 1, 2 | In this study | OR195160 | OR197369 | Spain: Olot (Girona) |
|  | GIR7_4 | 1, 2 | In this study | OR195161 | OR197370 | Spain: Olot (Girona) |
|  | GIR7_5 | 1, 2 | In this study | OR195162 | OR197371 | Spain: Olot (Girona) |
|  | GIR7_6 | 1, 2 | In this study | OR195163 | OR197372 | Spain: Olot (Girona) |
|  | GIR7_7 | 1, 2 | In this study | OR195164 | OR197373 | Spain: Olot (Girona) |
|  | GIR7_8 | 1, 2 | In this study | OR195165 | OR197374 | Spain: Olot (Girona) |
|  | GIR7_9 | 1, 2 | In this study | OR195166 | OR197375 | Spain: Olot (Girona) |
|  | GIR7_10 | 1, 2 | In this study | OR195166 | OR197375 | Spain: Olot (Girona) |
| **GIR8** | ID652 | 1 | Pedraza-Lara et al., 2010 | HM622595 | HM622601 | Spain: Garrotxa (Girona) |
|  | ID653 | 1 | Pedraza-Lara et al., 2010 | HM622595 | HM622601 | Spain: Garrotxa (Girona) |
|  | ID654 | 1 | Pedraza-Lara et al., 2010 | HM622595 | HM622601 | Spain: Garrotxa (Girona) |
|  | ID655 | 1 | Pedraza-Lara et al., 2010 | HM622595 | HM622601 | Spain: Garrotxa (Girona) |
|  | ID656 | 1 | Pedraza-Lara et al., 2010 | HM622595 | HM622601 | Spain: Garrotxa (Girona) |
|  | ID657 | 1 | Pedraza-Lara et al., 2010 | HM622595 | HM622601 | Spain: Garrotxa (Girona) |
|  | ID658 | 1 | Pedraza-Lara et al., 2010 | HM622595 | HM622601 | Spain: Garrotxa (Girona) |
|  | ID659 | 1 | Pedraza-Lara et al., 2010 | HM622595 | HM622601 | Spain: Garrotxa (Girona) |
|  | ID660 | 1 | Pedraza-Lara et al., 2010 | HM622595 | HM622601 | Spain: Garrotxa (Girona) |
|  | ID661 | 1 | Pedraza-Lara et al., 2010 | HM622595 | HM622609 | Spain: Garrotxa (Girona) |
| **GIR9** | ID662 | 1 | Pedraza-Lara et al., 2010 | HM622595 | HM622601 | Spain: Albera (Girona) |
|  | ID663 | 1 | Pedraza-Lara et al., 2010 | HM622595 | HM622601 | Spain: Albera (Girona) |
|  | ID664 | 1 | Pedraza-Lara et al., 2010 | HM622595 | HM622601 | Spain: Albera (Girona) |
|  | ID665 | 1 | Pedraza-Lara et al., 2010 | HM622595 | HM622601 | Spain: Albera (Girona) |
|  | ID666 | 1 | Pedraza-Lara et al., 2010 | HM622595 | HM622601 | Spain: Albera (Girona) |
|  | ID667 | 1 | Pedraza-Lara et al., 2010 | HM622595 | HM622601 | Spain: Albera (Girona) |
|  | ID668 | 1 | Pedraza-Lara et al., 2010 | HM622595 | HM622601 | Spain: Albera (Girona) |
|  | ID669 | 1 | Pedraza-Lara et al., 2010 | HM622595 | HM622601 | Spain: Albera (Girona) |
|  | ID670 | 1 | Pedraza-Lara et al., 2010 | HM622595 | HM622610 | Spain: Albera (Girona) |
|  | ID671 | 1 | Pedraza-Lara et al., 2010 | HM622595 | HM622611 | Spain: Albera (Girona) |
| **GIR10** | ID672 | 1 | Pedraza-Lara et al., 2010 | HM622596 | HM622612 | Spain: Salines (Girona) |
|  | ID673 | 1 | Pedraza-Lara et al., 2010 | HM622596 | HM622612 | Spain: Salines (Girona) |
|  | ID674 | 1 | Pedraza-Lara et al., 2010 | HM622596 | HM622612 | Spain: Salines (Girona) |
|  | ID675 | 1 | Pedraza-Lara et al., 2010 | HM622596 | HM622612 | Spain: Salines (Girona) |
|  | ID676 | 1 | Pedraza-Lara et al., 2010 | HM622596 | HM622612 | Spain: Salines (Girona) |
|  | ID677 | 1 | Pedraza-Lara et al., 2010 | HM622596 | HM622612 | Spain: Salines (Girona) |
|  | ID678 | 1 | Pedraza-Lara et al., 2010 | HM622596 | HM622612 | Spain: Salines (Girona) |
|  | ID679 | 1 | Pedraza-Lara et al., 2010 | HM622596 | HM622612 | Spain: Salines (Girona) |
|  | ID680 | 1 | Pedraza-Lara et al., 2010 | HM622596 | HM622612 | Spain: Salines (Girona) |
|  | ID681 | 1 | Pedraza-Lara et al., 2010 | HM622596 | HM622612 | Spain: Salines (Girona) |
|  | ID682 | 1 | Pedraza-Lara et al., 2010 | HM622596 | HM622612 | Spain: Salines (Girona) |
|  | ID683 | 1 | Pedraza-Lara et al., 2010 | HM622596 | HM622612 | Spain: Salines (Girona) |
|  | ID684 | 1 | Pedraza-Lara et al., 2010 | HM622596 | HM622612 | Spain: Salines (Girona) |
|  | ID685 | 1 | Pedraza-Lara et al., 2010 | HM622596 | HM622612 | Spain: Salines (Girona) |
|  | ID686 | 1 | Pedraza-Lara et al., 2010 | HM622596 | HM622612 | Spain: Salines (Girona) |
|  | ID687 | 1 | Pedraza-Lara et al., 2010 | HM622596 | HM622612 | Spain: Salines (Girona) |
|  | ID688 | 1 | Pedraza-Lara et al., 2010 | HM622596 | HM622613 | Spain: Salines (Girona) |
|  | ID689 | 1 | Pedraza-Lara et al., 2010 | HM622596 | HM622613 | Spain: Salines (Girona) |
|  | ID690 | 1 | Pedraza-Lara et al., 2010 | HM622596 | HM622613 | Spain: Salines (Girona) |
|  | ID691 | 1 | Pedraza-Lara et al., 2010 | HM622596 | HM622613 | Spain: Salines (Girona) |
|  | ID692 | 1 | Pedraza-Lara et al., 2010 | HM622596 | HM622613 | Spain: Salines (Girona) |
| **GIR11** | ID697 | 1 | Pedraza-Lara et al., 2010 | HM622595 | HM622601 | Spain: Riudaria (Girona) |
|  | ID698 | 1 | Pedraza-Lara et al., 2010 | HM622595 | HM622601 | Spain: Riudaria (Girona) |
|  | ID699 | 1 | Pedraza-Lara et al., 2010 | HM622595 | HM622601 | Spain: Riudaria (Girona) |
| **GIR12** | ID700 | 1 | Pedraza-Lara et al., 2010 | HM622595 | HM622601 | Spain: Les Preses (Girona) |
|  | ID701 | 1 | Pedraza-Lara et al., 2010 | HM622595 | HM622601 | Spain: Les Preses (Girona) |
|  | ID702 | 1 | Pedraza-Lara et al., 2010 | HM622595 | HM622601 | Spain: Les Preses (Girona) |
| **GRA1** | GRA1_1 | 1, 2 | Matallanas et al., 2016 | JF430574 | FJ897840 | Spain: Albuñuelas (Granada) |
|  | GRA1_2 | 1, 2 | Matallanas et al., 2016 | JF430574 | FJ897840 | Spain: Albuñuelas (Granada) |
|  | GRA1_3 | 1, 2 | Matallanas et al., 2016 | JF430574 | FJ897840 | Spain: Albuñuelas (Granada) |
|  | GRA1_4 | 1, 2 | Matallanas et al., 2016 | JF430574 | FJ897840 | Spain: Albuñuelas (Granada) |
|  | GRA1_5 | 1, 2 | Matallanas et al., 2016 | JF430574 | FJ897840 | Spain: Albuñuelas (Granada) |
|  | GRA1_6 | 1, 2 | Matallanas et al., 2016 | JF430574 | FJ897840 | Spain: Albuñuelas (Granada) |
|  | GRA1_7 | 1, 2 | Matallanas et al., 2016 | JF430574 | FJ897840 | Spain: Albuñuelas (Granada) |
|  | GRA1_8 | 1, 2 | Matallanas et al., 2016 | JF430574 | FJ897840 | Spain: Albuñuelas (Granada) |
|  | GRA1_9 | 1, 2 | Matallanas et al., 2016 | JF430574 | FJ897840 | Spain: Albuñuelas (Granada) |
|  | GRA1_10 | 1, 2 | Matallanas et al., 2016 | JF430574 | FJ897840 | Spain: Albuñuelas (Granada) |
| **GRA2** | GRA2_1 | 1, 2 | In this study | OR195167 | OR197376 | Spain: Río Blanco (Granada) |
|  | GRA2_2 | 1, 2 | In this study | OR195168 | OR197377 | Spain: Río Blanco (Granada) |
|  | GRA2_3 | 1, 2 | In this study | OR195169 | OR197378 | Spain: Río Blanco (Granada) |
|  | GRA2_4 | 1, 2 | In this study | OR195170 | OR197379 | Spain: Río Blanco (Granada) |
|  | GRA2_5 | 1, 2 | In this study | OR195171 | OR197380 | Spain: Río Blanco (Granada) |
|  | ID539 | 1 | Pedraza-Lara et al., 2010 | HM622595 | HM622606 | Spain: Río Blanco (Granada) |
| **GRA3** | GRA3_1 | 1, 2 | In this study | OR195172 | OR197381 | Spain: Bermejo (Granada) |
|  | GRA3_2 | 1, 2 | In this study | OR195173 | OR197382 | Spain: Bermejo (Granada) |
|  | GRA3_3 | 1, 2 | In this study | OR195174 | OR197383 | Spain: Bermejo (Granada) |
|  | GRA3_4 | 1, 2 | In this study | OR195175 | OR197384 | Spain: Bermejo (Granada) |
|  | GRA3_5 | 1, 2 | In this study | OR195176 | OR197385 | Spain: Bermejo (Granada) |
|  | GRA3_6 | 1, 2 | In this study | OR195177 | OR197386 | Spain: Bermejo (Granada) |
| **GRA4** | GRA4_1 | 1, 2 | In this study | OR195178 | OR197387 | Spain: Balsa de anfibios (Granada) |
|  | GRA4_2 | 1, 2 | In this study | OR195179 | OR197388 | Spain: Balsa de anfibios (Granada) |
|  | GRA4_3 | 1, 2 | In this study | OR195180 | OR197389 | Spain: Balsa de anfibios (Granada) |
|  | GRA4_4 | 1, 2 | In this study | OR195181 | OR197390 | Spain: Balsa de anfibios (Granada) |
|  | GRA4_5 | 1, 2 | In this study | OR195182 | OR197391 | Spain: Balsa de anfibios (Granada) |
|  | GRA4_6 | 1, 2 | In this study | OR195183 | OR197392 | Spain: Balsa de anfibios (Granada) |
|  | GRA4_7 | 1, 2 | In this study | OR195184 | OR197393 | Spain: Balsa de anfibios (Granada) |
| **GRA5** | GRA5_1 | 1, 2 | In this study | OR195185 | OR197394 | Spain: Almijara (Granada) (Granada) |
|  | GRA5_2 | 1, 2 | In this study | OR195186 | OR197395 | Spain: Almijara (Granada) (Granada) |
|  | GRA5_3 | 1, 2 | In this study | OR195187 | OR197396 | Spain: Almijara (Granada) (Granada) |
|  | GRA5_4 | 1, 2 | In this study | OR195188 | OR197397 | Spain: Almijara (Granada) (Granada) |
|  | GRA5_5 | 1, 2 | In this study | OR195189 | OR197398 | Spain: Almijara (Granada) (Granada) |
| **GRA6** | GRA6_1 | 1, 2 | In this study | OR195190 | OR197399 | Spain: Mazajate (Granada) |
|  | GRA6_2 | 1, 2 | In this study | OR195191 | OR197400 | Spain: Mazajate (Granada) |
|  | GRA6_3 | 1, 2 | In this study | OR195192 | OR197401 | Spain: Mazajate (Granada) |
|  | GRA6_4 | 1, 2 | In this study | OR195193 | OR197402 | Spain: Mazajate (Granada) |
|  | GRA6_5 | 1, 2 | In this study | OR195194 | OR197403 | Spain: Mazajate (Granada) |
| **GRA7** | GRA7_1 | 1, 2 | In this study | OR195195 | OR197404 | Spain: Cazín (Granada) |
|  | GRA7_2 | 1, 2 | In this study | OR195196 | OR197405 | Spain: Cazín (Granada) |
|  | GRA7_3 | 1, 2 | In this study | OR195197 | OR197406 | Spain: Cazín (Granada) |
|  | GRA7_4 | 1, 2 | In this study | OR195198 | OR197407 | Spain: Cazín (Granada) |
|  | GRA7_5 | 1, 2 | In this study | OR195199 | OR197408 | Spain: Cazín (Granada) |
|  | GRA7_6 | 1, 2 | In this study | OR195200 | OR197409 | Spain: Cazín (Granada) |
| **GRA8** | GRA8_1 | 1, 2 | In this study | OR195222 | OR197431 | Spain: Arroyo Toba (Granada) |
|  | GRA8_2 | 1, 2 | In this study | OR195223 | OR197432 | Spain: Arroyo Toba (Granada) |
|  | GRA8_3 | 1, 2 | In this study | OR195224 | OR197433 | Spain: Arroyo Toba (Granada) |
|  | GRA8_4 | 1, 2 | In this study | OR195225 | OR197434 | Spain: Arroyo Toba (Granada) |
|  | GRA8_5 | 1, 2 | In this study | OR195226 | OR197435 | Spain: Arroyo Toba (Granada) |
| **GRA9** | ID524 | 1 | Pedraza-Lara et al., 2010 | HM622595 | HM622601 | Spain: Cabaneros (Granada) |
|  | ID525 | 1 | Pedraza-Lara et al., 2010 | HM622595 | HM622604 | Spain: Cabaneros (Granada) |
|  | ID526 | 1 | Pedraza-Lara et al., 2010 | HM622595 | HM622605 | Spain: Cabaneros (Granada) |
| **GRA10** | ID527 | 1 | Pedraza-Lara et al., 2010 | HM622595 | HM622601 | Spain: Almijara (Granada) |
|  | ID528 | 1 | Pedraza-Lara et al., 2010 | HM622595 | HM622601 | Spain: Almijara (Granada) |
|  | ID529 | 1 | Pedraza-Lara et al., 2010 | HM622595 | HM622601 | Spain: Almijara (Granada) |
| **GRA11** | ID530 | 1 | Pedraza-Lara et al., 2010 | HM622595 | HM622601 | Spain: Arenas del Rey (Granada) |
|  | ID531 | 1 | Pedraza-Lara et al., 2010 | HM622595 | HM622601 | Spain: Arenas del Rey (Granada) |
|  | ID532 | 1 | Pedraza-Lara et al., 2010 | HM622595 | HM622601 | Spain: Arenas del Rey (Granada) |
| **GRA12** | ID533 | 1 | Pedraza-Lara et al., 2010 | HM622595 | HM622601 | Spain: Sillar Baja (Granada) |
|  | ID534 | 1 | Pedraza-Lara et al., 2010 | HM622595 | HM622601 | Spain: Sillar Baja (Granada) |
|  | ID535 | 1 | Pedraza-Lara et al., 2010 | HM622595 | HM622601 | Spain: Sillar Baja (Granada) |
| **GRA13** | ID536 | 1 | Pedraza-Lara et al., 2010 | HM622595 | HM622601 | Spain: Fraile (Granada) |
|  | ID537 | 1 | Pedraza-Lara et al., 2010 | HM622595 | HM622601 | Spain: Fraile (Granada) |
|  | ID538 | 1 | Pedraza-Lara et al., 2010 | HM622595 | HM622601 | Spain: Fraile (Granada) |
| **GRA14** | ID540 | 1 | Pedraza-Lara et al., 2010 | HM622595 | HM622601 | Spain: Loja (Granada) |
|  | ID541 | 1 | Pedraza-Lara et al., 2010 | HM622595 | HM622601 | Spain: Loja (Granada) |
| **GRA15** | ID542 | 1 | Pedraza-Lara et al., 2010 | HM622595 | HM622601 | Spain: Quentar (Granada) |
|  | ID543 | 1 | Pedraza-Lara et al., 2010 | HM622595 | HM622601 | Spain: Quentar (Granada) |
|  | ID544 | 1 | Pedraza-Lara et al., 2010 | HM622595 | HM622601 | Spain: Quentar (Granada) |
| **GRA16** | ID545 | 1 | Pedraza-Lara et al., 2010 | HM622595 | HM622601 | Spain: Gojar (Granada) |
|  | ID546 | 1 | Pedraza-Lara et al., 2010 | HM622595 | HM622601 | Spain: Gojar (Granada) |
|  | ID547 | 1 | Pedraza-Lara et al., 2010 | HM622595 | HM622601 | Spain: Gojar (Granada) |
| **GRA17** | ID607 | 1 | Pedraza-Lara et al., 2010 | HM622595 | HM622601 | Spain: Guajar (Granada) |
|  | ID608 | 1 | Pedraza-Lara et al., 2010 | HM622595 | HM622601 | Spain: Guajar (Granada) |
|  | ID609 | 1 | Pedraza-Lara et al., 2010 | HM622595 | HM622601 | Spain: Guajar (Granada) |
| **GU1** | GU1_1 | 1, 2 | Martín-Torrijos et al., 2021 | MW327215 | MW325399 | Spain: Chaparrillo (Guadalajara) |
|  | GU1_2 | 1, 2 | Martín-Torrijos et al., 2021 | MW327216 | MW325480 | Spain: Chaparrillo (Guadalajara) |
|  | GU1_3 | 1, 2 | Martín-Torrijos et al., 2021 | MW327340 | MW325481 | Spain: Chaparrillo (Guadalajara) |
|  | GU1_4 | 1, 2 | Martín-Torrijos et al., 2021 | MW327217 | MW325453 | Spain: Chaparrillo (Guadalajara) |
|  | GU1_5 | 1, 2 | Martín-Torrijos et al., 2021 | MW327218 | MW325482 | Spain: Chaparrillo (Guadalajara) |
| **GU2** | GU2_1 | 1, 2 | Matallanas et al., 2016 | JF430574 | FJ897840 | Spain: Río Gallo (Guadalajara) |
|  | GU2_2 | 1, 2 | Matallanas et al., 2016 | JF430574 | FJ897840 | Spain: Río Gallo (Guadalajara) |
|  | GU2_3 | 1, 2 | Matallanas et al., 2016 | JF430574 | FJ897840 | Spain: Río Gallo (Guadalajara) |
|  | GU2_4 | 1, 2 | Matallanas et al., 2016 | JF430574 | FJ897840 | Spain: Río Gallo (Guadalajara) |
|  | GU2_5 | 1, 2 | Matallanas et al., 2016 | JF430574 | FJ897840 | Spain: Río Gallo (Guadalajara) |
|  | GU2_6 | 1, 2 | Matallanas et al., 2016 | JF430574 | FJ897841 | Spain: Río Gallo (Guadalajara) |
|  | GU2_7 | 1, 2 | Matallanas et al., 2016 | JF430574 | FJ897841 | Spain: Río Gallo (Guadalajara) |
|  | GU2_8 | 1, 2 | Matallanas et al., 2016 | JF430574 | FJ897841 | Spain: Río Gallo (Guadalajara) |
|  | GU2_9 | 1, 2 | Matallanas et al., 2016 | JF430574 | FJ897841 | Spain: Río Gallo (Guadalajara) |
|  | GU2_10 | 1, 2 | Matallanas et al., 2016 | JF430574 | FJ897841 | Spain: Río Gallo (Guadalajara) |
| **HU1** | HU1_1 | 1, 2 | Martín-Torrijos et al., 2021 | MW327219 | MW325496 | Spain: Barranco Villano (Huesca) |
|  | HU1_2 | 1, 2 | Martín-Torrijos et al., 2021 | MW327220 | MW325498 | Spain: Barranco Villano (Huesca) |
|  | HU1_3 | 1, 2 | Martín-Torrijos et al., 2021 | MW327221 | MW325499 | Spain: Barranco Villano (Huesca) |
|  | HU1_4 | 1, 2 | Martín-Torrijos et al., 2021 | MW327222 | MW325500 | Spain: Barranco Villano (Huesca) |
|  | HU1_5 | 1, 2 | Martín-Torrijos et al., 2021 | MW327223 | MW325497 | Spain: Barranco Villano (Huesca) |
| **HU2** | HU2_1 | 1, 2 | Martín-Torrijos et al., 2021 | MW327224 | MW325351 | Spain: Formiga (Huesca) |
|  | HU2_3 | 1, 2 | Martín-Torrijos et al., 2021 | MW327225 | MW325352 | Spain: Formiga (Huesca) |
| **HU3** | HU3_1 | 1, 2 | Matallanas et al., 2016 | JF430574 | FJ897840 | Spain: Casbas (Huesca) |
|  | HU3_2 | 1, 2 | Matallanas et al., 2016 | JF430574 | FJ897840 | Spain: Casbas (Huesca) |
|  | HU3_3 | 1, 2 | Matallanas et al., 2016 | JF430574 | FJ897840 | Spain: Casbas (Huesca) |
|  | HU3_4 | 1, 2 | Matallanas et al., 2016 | JF430574 | FJ897840 | Spain: Casbas (Huesca) |
|  | HU3_5 | 1, 2 | Matallanas et al., 2016 | JF430574 | FJ897840 | Spain: Casbas (Huesca) |
|  | HU3_6 | 1, 2 | Matallanas et al., 2016 | JF430576 | FJ897840 | Spain: Casbas (Huesca) |
|  | HU3_7 | 1, 2 | Matallanas et al., 2016 | JF430574 | JF430567 | Spain: Casbas (Huesca) |
|  | HU3_8 | 1, 2 | Matallanas et al., 2016 | EF489427 | EF485041 | Spain: Casbas (Huesca) |
|  | HU3_9 | 1, 2 | Matallanas et al., 2016 | EF489427 | EF485041 | Spain: Casbas (Huesca) |
|  | HU3_10 | 1, 2 | Matallanas et al., 2016 | EF489427 | EF485041 | Spain: Casbas (Huesca) |
| **HU4** | HU4_1 | 1, 2 | In this study | OR195201 | OR197410 | Spain: Ansó (Huesca) |
|  | HU4_2 | 1, 2 | In this study | OR195202 | OR197411 | Spain: Ansó (Huesca) |
|  | HU4_3 | 1, 2 | In this study | OR195203 | OR197412 | Spain: Ansó (Huesca) |
|  | HU4_4 | 1, 2 | In this study | OR195204 | OR197413 | Spain: Ansó (Huesca) |
|  | HU4_5 | 1, 2 | In this study | OR195205 | OR197414 | Spain: Ansó (Huesca) |
| **HU5** | HU5_2 | 1, 2 | In this study | OR195206 | OR197415 | Spain: Agüero (Huesca) |
|  | HU5_4 | 1, 2 | In this study | OR195207 | OR197416 | Spain: Agüero (Huesca) |
|  | HU5_5 | 1, 2 | In this study | OR195208 | OR197417 | Spain: Agüero (Huesca) |
| **HU6** | HU6_2 | 1, 2 | In this study | OR195209 | OR197418 | Spain: Loporzano (Huesca) |
|  | HU6_4 | 1, 2 | In this study | OR195210 | OR197419 | Spain: Loporzano (Huesca) |
| **HU7** | ID548 | 1 | Pedraza-Lara et al., 2010 | HM622595 | HM622601 | Spain: Bco de Fago (Huesca) |
|  | ID549 | 1 | Pedraza-Lara et al., 2010 | HM622595 | HM622601 | Spain: Bco de Fago (Huesca) |
|  | ID550 | 1 | Pedraza-Lara et al., 2010 | HM622595 | HM622603 | Spain: Bco de Fago (Huesca) |
| **HU8** | ID584 | 1 | Pedraza-Lara et al., 2010 | HM622595 | HM622603 | Spain: Hornero (Huesca) |
|  | ID585 | 1 | Pedraza-Lara et al., 2010 | HM622595 | HM622603 | Spain: Hornero (Huesca) |
|  | ID586 | 1 | Pedraza-Lara et al., 2010 | HM622595 | HM622603 | Spain: Hornero (Huesca) |
| **IT1** | IT1_1 | 1, 2 | Matallanas et al., 2016 | JF430574 | FJ897840 | Italy: Florence and Prato |
|  | IT1_2 | 1, 2 | Matallanas et al., 2016 | JF430574 | FJ897840 | Italy: Florence and Prato |
|  | IT1_3 | 1, 2 | Matallanas et al., 2016 | JF430574 | FJ897840 | Italy: Florence and Prato |
|  | IT1_4 | 1, 2 | Matallanas et al., 2016 | JF430574 | FJ897840 | Italy: Florence and Prato |
|  | IT1_5 | 1, 2 | Matallanas et al., 2016 | JF430582 | FJ897840 | Italy: Florence and Prato |
|  | IT1_6 | 1, 2 | Matallanas et al., 2016 | JF430582 | FJ897840 | Italy: Florence and Prato |
|  | IT1_7 | 1, 2 | Matallanas et al., 2016 | JF430582 | FJ897840 | Italy: Florence and Prato |
|  | IT1_8 | 1, 2 | Matallanas et al., 2016 | JF430574 | JF430571 | Italy: Florence and Prato |
|  | IT1_9 | 1, 2 | Matallanas et al., 2016 | JF430574 | JF430571 | Italy: Florence and Prato |
|  | IT1_10 | 1, 2 | Matallanas et al., 2016 | JF430574 | JF430571 | Italy: Florence and Prato |
| **IT2** | IT2_1 | 1, 2 | In this study | OR195211 | OR197420 | Italy: Torrente Gambellato |
|  | IT2_2 | 1, 2 | In this study | OR195212 | OR197421 | Italy: Torrente Gambellato |
|  | IT2_4 | 1, 2 | In this study | OR195213 | OR197422 | Italy: Torrente Gambellato |
|  | IT2_5 | 1, 2 | In this study | OR195214 | OR197423 | Italy: Torrente Gambellato |
| **IT3** | IT3_3 | 1, 2 | In this study | OR195215 | OR197424 | Italy: Voglio River |
|  | IT3_7 | 1, 2 | In this study | OR195216 | OR197425 | Italy: Voglio River |
| **IT4** | IT4_1 | 1, 2 | In this study | OR195217 | OR197426 | Italy: Fornace River |
|  | IT4_2 | 1, 2 | In this study | OR195218 | OR197427 | Italy: Fornace River |
|  | IT4_3 | 1, 2 | In this study | OR195219 | OR197428 | Italy: Fornace River |
|  | IT4_4 | 1, 2 | In this study | OR195220 | OR197429 | Italy: Fornace River |
|  | IT4_5 | 1, 2 | In this study | OR195221 | OR197430 | Italy: Fornace River |
| **IT5** | ID172 | 1 | Jelić et al., 2016 | KX370260 | KX369840 | Italy: Rosandra |
|  | ID173 | 1 | Jelić et al., 2016 | KX370261 | KX369841 | Italy: Rosandra |
|  | ID174 | 1 | Jelić et al., 2016 | KX370262 | KX369842 | Italy: Rosandra |
| **IT6** | ID175 | 1 | Jelić et al., 2016 | KX370263 | KX369843 | Italy: Sorgive del Sile |
|  | ID176 | 1 | Jelić et al., 2016 | KX370264 | KX369844 | Italy: Sorgive del Sile |
| **IT7** | ID177 | 1 | Jelić et al., 2016 | KX370265 | KX369845 | Italy: Inglagna |
|  | ID178 | 1 | Jelić et al., 2016 | KX370266 | KX369846 | Italy: Inglagna |
|  | ID179 | 1 | Jelić et al., 2016 | KX370267 | KX369847 | Italy: Inglagna |
|  | ID180 | 1 | Jelić et al., 2016 | KX370268 | KX369848 | Italy: Inglagna |
|  | ID181 | 1 | Jelić et al., 2016 | KX370269 | KX369849 | Italy: Inglagna |
|  | ID182 | 1 | Jelić et al., 2016 | KX370270 | KX369850 | Italy: Inglagna |
| **IT8** | ID183 | 1 | Jelić et al., 2016 | KX370271 | KX369851 | Italy: Sorgive Zamlis |
|  | ID184 | 1 | Jelić et al., 2016 | KX370272 | KX369852 | Italy: Sorgive Zamlis |
|  | ID185 | 1 | Jelić et al., 2016 | KX370273 | KX369853 | Italy: Sorgive Zamlis |
|  | ID186 | 1 | Jelić et al., 2016 | KX370274 | KX369854 | Italy: Sorgive Zamlis |
| **IT9** | ID187 | 1 | Jelić et al., 2016 | KX370275 | KX369855 | Italy: Rio Gamberi |
|  | ID188 | 1 | Jelić et al., 2016 | KX370276 | KX369856 | Italy: Rio Gamberi |
|  | ID189 | 1 | Jelić et al., 2016 | KX370277 | KX369857 | Italy: Rio Gamberi |
| **IT10** | ID190 | 1 | Jelić et al., 2016 | KX370278 | KX369858 | Italy: Rio Luscovazu |
|  | ID191 | 1 | Jelić et al., 2016 | KX370279 | KX369859 | Italy: Rio Luscovazu |
| **IT11** | ID200 | 1 | Jelić et al., 2016 | KX370288 | KX369868 | Italy: Sorgenti del Natisone |
|  | ID201 | 1 | Jelić et al., 2016 | KX370289 | KX369869 | Italy: Sorgenti del Natisone |
|  | ID202 | 1 | Jelić et al., 2016 | KX370290 | KX369870 | Italy: Sorgenti del Natisone |
| **IT12** | ID203 | 1 | Jelić et al., 2016 | KX370291 | KX369871 | Italy: Rio Valcada |
|  | ID204 | 1 | Jelić et al., 2016 | KX370292 | KX369872 | Italy: Rio Valcada |
|  | ID205 | 1 | Jelić et al., 2016 | KX370293 | KX369873 | Italy: Rio Valcada |
| **IT13** | ID234 | 1 | Jelić et al., 2016 | KX370322 | KX369902 | Italy: Bologna |
|  | ID235 | 1 | Jelić et al., 2016 | KX370323 | KX369903 | Italy: Bologna |
|  | ID236 | 1 | Jelić et al., 2016 | KX370324 | KX369904 | Italy: Bologna |
|  | ID237 | 1 | Jelić et al., 2016 | KX370325 | KX369905 | Italy: Bologna |
|  | ID238 | 1 | Jelić et al., 2016 | KX370326 | KX369906 | Italy: Bologna |
|  | ID239 | 1 | Jelić et al., 2016 | KX370327 | KX369907 | Italy: Bologna |
|  | ID240 | 1 | Jelić et al., 2016 | KX370328 | KX369908 | Italy: Bologna |
|  | ID241 | 1 | Jelić et al., 2016 | KX370329 | KX369909 | Italy: Bologna |
|  | ID242 | 1 | Jelić et al., 2016 | KX370330 | KX369910 | Italy: Bologna |
|  | ID243 | 1 | Jelić et al., 2016 | KX370331 | KX369911 | Italy: Bologna |
|  | ID244 | 1 | Jelić et al., 2016 | KX370332 | KX369912 | Italy: Bologna |
|  | ID245 | 1 | Jelić et al., 2016 | KX370333 | KX369913 | Italy: Bologna |
| **IT14** | ID297 | 1 | Jelić et al., 2016 | KX370385 | KX369965 | Italy: Firenze |
|  | ID298 | 1 | Jelić et al., 2016 | KX370386 | KX369966 | Italy: Firenze |
|  | ID299 | 1 | Jelić et al., 2016 | KX370387 | KX369967 | Italy: Firenze |
|  | ID300 | 1 | Jelić et al., 2016 | KX370388 | KX369968 | Italy: Firenze |
|  | ID301 | 1 | Jelić et al., 2016 | KX370389 | KX369969 | Italy: Firenze |
|  | ID302 | 1 | Jelić et al., 2016 | KX370390 | KX369970 | Italy: Firenze |
|  | ID303 | 1 | Jelić et al., 2016 | KX370391 | KX369971 | Italy: Firenze |
|  | ID304 | 1 | Jelić et al., 2016 | KX370392 | KX369972 | Italy: Firenze |
|  | ID305 | 1 | Jelić et al., 2016 | KX370393 | KX369973 | Italy: Firenze |
|  | ID306 | 1 | Jelić et al., 2016 | KX370394 | KX369974 | Italy: Firenze |
|  | ID307 | 1 | Jelić et al., 2016 | KX370395 | KX369975 | Italy: Firenze |
|  | ID308 | 1 | Jelić et al., 2016 | KX370396 | KX369976 | Italy: Firenze |
|  | ID309 | 1 | Jelić et al., 2016 | KX370397 | KX369977 | Italy: Firenze |
| **IT15** | ID329 | 1 | Jelić et al., 2016 | KX370417 | KX369997 | Italy: Genoa |
|  | ID330 | 1 | Jelić et al., 2016 | KX370418 | KX369998 | Italy: Genoa |
|  | ID331 | 1 | Jelić et al., 2016 | KX370419 | KX369999 | Italy: Genoa |
|  | ID332 | 1 | Jelić et al., 2016 | KX370420 | KX370000 | Italy: Genoa |
|  | ID333 | 1 | Jelić et al., 2016 | KX370421 | KX370001 | Italy: Genoa |
|  | ID334 | 1 | Jelić et al., 2016 | KX370422 | KX370002 | Italy: Genoa |
|  | ID335 | 1 | Jelić et al., 2016 | KX370423 | KX370003 | Italy: Genoa |
|  | ID336 | 1 | Jelić et al., 2016 | KX370424 | KX370004 | Italy: Genoa |
|  | ID337 | 1 | Jelić et al., 2016 | KX370425 | KX370005 | Italy: Genoa |
|  | ID338 | 1 | Jelić et al., 2016 | KX370426 | KX370006 | Italy: Genoa |
| **IT16** | ID418 | 1 | Jelić et al., 2016 | KX370506 | KX370086 | Italy: Sorgenti del Natisone |
|  | ID419 | 1 | Jelić et al., 2016 | KX370507 | KX370087 | Italy: Sorgenti del Natisone |
| **IT17** | ID420 | 1 | Jelić et al., 2016 | KX370508 | KX370088 | Italy: Rio Valcada |
|  | ID421 | 1 | Jelić et al., 2016 | KX370509 | KX370089 | Italy: Rio Valcada |
| **IT18** | ID424 | 1 | Cataudella et al., 2010 | KX370512 | EU077566 | Italy: Pozzatoio |
|  | ID425 | 1 | Cataudella et al., 2010 | KX370513 | EU077567 | Italy: Pozzatoio |
|  | ID426 | 1 | Cataudella et al., 2010 | KX370514 | EU077567 | Italy: Pozzatoio |
|  | ID427 | 1 | Cataudella et al., 2010 | KX370515 | EU077567 | Italy: Pozzatoio |
|  | ID428 | 1 | Cataudella et al., 2010 | KX370516 | EU077567 | Italy: Pozzatoio |
|  | ID429 | 1 | Cataudella et al., 2010 | KX370517 | EU077567 | Italy: Pozzatoio |
|  | ID430 | 1 | Cataudella et al., 2010 | KX370518 | EU077567 | Italy: Pozzatoio |
|  | ID431 | 1 | Cataudella et al., 2010 | KX370519 | EU077573 | Italy: Pozzatoio |
|  | ID432 | 1 | Cataudella et al., 2010 | KX370520 | EU077573 | Italy: Pozzatoio |
|  | ID433 | 1 | Cataudella et al., 2010 | KX370521 | EU077573 | Italy: Pozzatoio |
|  | ID434 | 1 | Cataudella et al., 2010 | KX370522 | EU077573 | Italy: Pozzatoio |
|  | ID435 | 1 | Cataudella et al., 2010 | KX370523 | EU077573 | Italy: Pozzatoio |
|  | ID436 | 1 | Cataudella et al., 2010 | KX370524 | EU077573 | Italy: Pozzatoio |
|  | ID437 | 1 | Cataudella et al., 2010 | KX370525 | EU077573 | Italy: Pozzatoio |
| **IT19** | ID438 | 1 | Cataudella et al., 2010 | KX370526 | EU077568 | Italy: Amandole |
|  | ID439 | 1 | Cataudella et al., 2010 | KX370527 | EU077568 | Italy: Amandole |
|  | ID440 | 1 | Cataudella et al., 2010 | KX370528 | EU077569 | Italy: Amandole |
|  | ID441 | 1 | Cataudella et al., 2010 | KX370529 | EU077573 | Italy: Amandole |
|  | ID442 | 1 | Cataudella et al., 2010 | KX370530 | EU077570 | Italy: Amandole |
|  | ID443 | 1 | Cataudella et al., 2010 | KX370531 | EU077570 | Italy: Amandole |
|  | ID444 | 1 | Cataudella et al., 2010 | KX370532 | EU077570 | Italy: Amandole |
|  | ID445 | 1 | Cataudella et al., 2010 | KX370533 | EU077570 | Italy: Amandole |
|  | ID446 | 1 | Cataudella et al., 2010 | KX370534 | EU077570 | Italy: Amandole |
|  | ID447 | 1 | Cataudella et al., 2010 | KX370535 | EU077570 | Italy: Amandole |
|  | ID448 | 1 | Cataudella et al., 2010 | KX370536 | EU077570 | Italy: Amandole |
|  | ID449 | 1 | Cataudella et al., 2010 | KX370537 | EU077570 | Italy: Amandole |
| **IT20** | ID450 | 1 | Cataudella et al., 2010 | KX370538 | EU077570 | Italy: Tributary of Meta |
|  | ID451 | 1 | Cataudella et al., 2010 | KX370539 | EU077570 | Italy: Tributary of Meta |
|  | ID452 | 1 | Cataudella et al., 2010 | KX370540 | EU077570 | Italy: Tributary of Meta |
|  | ID453 | 1 | Cataudella et al., 2010 | KX370541 | EU077570 | Italy: Tributary of Meta |
|  | ID454 | 1 | Cataudella et al., 2010 | KX370542 | EU077570 | Italy: Tributary of Meta |
| **IT21** | ID460 | 1 | Cataudella et al., 2010 | KX370548 | EU077570 | Italy: Cesano |
|  | ID461 | 1 | Cataudella et al., 2010 | KX370549 | EU077570 | Italy: Cesano |
|  | ID462 | 1 | Cataudella et al., 2010 | KX370550 | EU077570 | Italy: Cesano |
|  | ID463 | 1 | Cataudella et al., 2010 | KX370551 | EU077570 | Italy: Cesano |
|  | ID464 | 1 | Cataudella et al., 2010 | KX370552 | EU077570 | Italy: Cesano |
| **IT22** | ID477 | 1 | Cataudella et al., 2010 | KX370565 | EU077570 | Italy: Rio Freddo |
|  | ID478 | 1 | Cataudella et al., 2010 | KX370566 | EU077570 | Italy: Rio Freddo |
|  | ID479 | 1 | Cataudella et al., 2010 | KX370567 | EU077570 | Italy: Rio Freddo |
|  | ID480 | 1 | Cataudella et al., 2010 | KX370568 | EU077570 | Italy: Rio Freddo |
|  | ID481 | 1 | Cataudella et al., 2010 | KX370569 | EU077570 | Italy: Rio Freddo |
| **IT23** | ID482 | 1 | Cataudella et al., 2010 | KX370570 | EU077571 | Italy: Canavi |
|  | ID483 | 1 | Cataudella et al., 2010 | KX370571 | EU077571 | Italy: Canavi |
|  | ID484 | 1 | Cataudella et al., 2010 | KX370572 | EU077571 | Italy: Canavi |
|  | ID485 | 1 | Cataudella et al., 2010 | KX370573 | EU077571 | Italy: Canavi |
|  | ID486 | 1 | Cataudella et al., 2010 | KX370574 | EU077571 | Italy: Canavi |
| **IT24** | ID487 | 1 | Cataudella et al., 2010 | KX370575 | EU077571 | Italy: Fosso del Lago |
|  | ID488 | 1 | Cataudella et al., 2010 | KX370576 | EU077571 | Italy: Fosso del Lago |
|  | ID489 | 1 | Cataudella et al., 2010 | KX370577 | EU077571 | Italy: Fosso del Lago |
|  | ID490 | 1 | Cataudella et al., 2010 | KX370578 | EU077571 | Italy: Fosso del Lago |
|  | ID491 | 1 | Cataudella et al., 2010 | KX370579 | EU077571 | Italy: Fosso del Lago |
| **IT25** | ID492 | 1 | Cataudella et al., 2010 | KX370580 | EU077571 | Italy: Lera |
|  | ID493 | 1 | Cataudella et al., 2010 | KX370581 | EU077571 | Italy: Lera |
|  | ID494 | 1 | Cataudella et al., 2010 | KX370582 | EU077571 | Italy: Lera |
|  | ID495 | 1 | Cataudella et al., 2010 | KX370583 | EU077571 | Italy: Lera |
|  | ID496 | 1 | Cataudella et al., 2010 | KX370584 | EU077571 | Italy: Lera |
| **IT26** | ID497 | 1 | Cataudella et al., 2010 | KX370585 | EU077572 | Italy: Battendiero |
|  | ID498 | 1 | Cataudella et al., 2010 | KX370586 | EU077572 | Italy: Battendiero |
| **IT27** | ID499 | 1 | Cataudella et al., 2010 | KX370587 | EU077573 | Italy: Coscile |
|  | ID500 | 1 | Cataudella et al., 2010 | KX370588 | EU077573 | Italy: Coscile |
|  | ID501 | 1 | Cataudella et al., 2010 | KX370589 | EU077573 | Italy: Coscile |
| **IT28** | ID705 | 1 | Pedraza-Lara et al., 2010 | HM622598 | HM622616 | Italy: Sovara Torrent |
|  | ID706 | 1 | Pedraza-Lara et al., 2010 | HM622598 | HM622616 | Italy: Sovara Torrent |
|  | ID707 | 1 | Pedraza-Lara et al., 2010 | HM622598 | HM622616 | Italy: Sovara Torrent |
|  | ID708 | 1 | Pedraza-Lara et al., 2010 | HM622598 | HM622616 | Italy: Sovara Torrent |
|  | ID709 | 1 | Pedraza-Lara et al., 2010 | HM622598 | HM622616 | Italy: Sovara Torrent |
|  | ID710 | 1 | Pedraza-Lara et al., 2010 | HM622598 | HM622616 | Italy: Sovara Torrent |
|  | ID711 | 1 | Pedraza-Lara et al., 2010 | HM622598 | HM622616 | Italy: Sovara Torrent |
|  | ID712 | 1 | Pedraza-Lara et al., 2010 | HM622598 | HM622616 | Italy: Sovara Torrent |
|  | ID713 | 1 | Pedraza-Lara et al., 2010 | HM622598 | HM622616 | Italy: Sovara Torrent |
|  | ID714 | 1 | Pedraza-Lara et al., 2010 | HM622598 | HM622616 | Italy: Sovara Torrent |
| **IT29** | ID715 | 1 | Pedraza-Lara et al., 2010 | HM622594 | HM622599 | Italy: Resco Simontano |
|  | ID716 | 1 | Pedraza-Lara et al., 2010 | HM622594 | HM622599 | Italy: Resco Simontano |
|  | ID717 | 1 | Pedraza-Lara et al., 2010 | HM622594 | HM622600 | Italy: Resco Simontano |
|  | ID718 | 1 | Pedraza-Lara et al., 2010 | HM622594 | HM622600 | Italy: Resco Simontano |
| **IT30** | ID719 | 1 | Pedraza-Lara et al., 2010 | HM622597 | HM622617 | Italy: Sondrio |
|  | ID720 | 1 | Pedraza-Lara et al., 2010 | HM622597 | HM622617 | Italy: Sondrio |
|  | ID721 | 1 | Pedraza-Lara et al., 2010 | HM622597 | HM622617 | Italy: Sondrio |
|  | ID722 | 1 | Pedraza-Lara et al., 2010 | HM622597 | HM622617 | Italy: Sondrio |
|  | ID723 | 1 | Pedraza-Lara et al., 2010 | HM622597 | HM622617 | Italy: Sondrio |
| **IRE1** | ID147 | 1 | Jelić et al., 2016 | KX370235 | KX369815 | Ireland: Brittas |
|  | ID148 | 1 | Jelić et al., 2016 | KX370236 | KX369816 | Ireland: Brittas |
| **IRE2** | ID214 | 1 | Jelić et al., 2016 | KX370302 | KX369882 | Ireland: Ballybouden |
|  | ID215 | 1 | Jelić et al., 2016 | KX370303 | KX369883 | Ireland: Ballybouden |
|  | ID216 | 1 | Jelić et al., 2016 | KX370304 | KX369884 | Ireland: Ballybouden |
|  | ID217 | 1 | Jelić et al., 2016 | KX370305 | KX369885 | Ireland: Ballybouden |
| **IRE3** | ID229 | 1 | Jelić et al., 2016 | KX370317 | KX369897 | Ireland: Blessington |
|  | ID230 | 1 | Jelić et al., 2016 | KX370318 | KX369898 | Ireland: Blessington |
|  | ID231 | 1 | Jelić et al., 2016 | KX370319 | KX369899 | Ireland: Blessington |
|  | ID232 | 1 | Jelić et al., 2016 | KX370320 | KX369900 | Ireland: Blessington |
|  | ID233 | 1 | Jelić et al., 2016 | KX370321 | KX369901 | Ireland: Blessington |
| **JA1** | JA1_3 | 1, 2 | Martín-Torrijos et al., 2021 | MW327308 | MW325349 | Spain: Cazorla (Jaén) |
|  | JA1_4 | 1, 2 | Martín-Torrijos et al., 2021 | MW327245 | MW325350 | Spain: Cazorla (Jaén) |
| **JA3** | JA3_1 | 1, 2 | In this study | OR195227 | OR197436 | Spain: Aguascebas chico (Jaén) |
|  | JA3_2 | 1, 2 | In this study | OR195228 | OR197437 | Spain: Aguascebas chico (Jaén) |
|  | JA3_3 | 1, 2 | In this study | OR195229 | OR197438 | Spain: Aguascebas chico (Jaén) |
|  | JA3_4 | 1, 2 | In this study | OR195230 | OR197439 | Spain: Aguascebas chico (Jaén) |
|  | JA3_5 | 1, 2 | In this study | OR195231 | OR197440 | Spain: Aguascebas chico (Jaén) |
| **JA4** | JA4_1 | 1, 2 | In this study | OR195232 | OR197441 | Spain: Aguasmulas (Jaén) |
|  | JA4_2 | 1, 2 | In this study | OR195233 | OR197442 | Spain: Aguasmulas (Jaén) |
|  | JA4_3 | 1, 2 | In this study | OR195234 | OR197443 | Spain: Aguasmulas (Jaén) |
|  | JA4_4 | 1, 2 | In this study | OR195235 | OR197444 | Spain: Aguasmulas (Jaén) |
|  | JA4_5 | 1, 2 | In this study | OR195236 | OR197445 | Spain: Aguasmulas (Jaén) |
| **JA5** | JA5_1 | 1, 2 | In this study | OR195237 | OR197446 | Spain: Borosa (Jaén) |
|  | JA5_2 | 1, 2 | In this study | OR195238 | OR197447 | Spain: Borosa (Jaén) |
|  | JA5_3 | 1, 2 | In this study | OR195239 | OR197448 | Spain: Borosa (Jaén) |
|  | JA5_4 | 1, 2 | In this study | OR195240 | OR197449 | Spain: Borosa (Jaén) |
|  | JA5_5 | 1, 2 | In this study | OR195241 | OR197450 | Spain: Borosa (Jaén) |
| **JA6** | JA6_1 | 1, 2 | In this study | OR195242 | OR197451 | Spain: Guadalquivir (Jaén) |
|  | JA6_2 | 1, 2 | In this study | OR195243 | OR197452 | Spain: Guadalquivir (Jaén) |
|  | JA6_3 | 1, 2 | In this study | OR195244 | OR197453 | Spain: Guadalquivir (Jaén) |
|  | JA6_4 | 1, 2 | In this study | OR195245 | OR197454 | Spain: Guadalquivir (Jaén) |
|  | JA6_5 | 1, 2 | In this study | OR195246 | OR197455 | Spain: Guadalquivir (Jaén) |
| **JA7** | ID502 | 1 | Pedraza-Lara et al., 2010 | HM622595 | HM622601 | Spain: Fresnedilla (Jaén) |
|  | ID503 | 1 | Pedraza-Lara et al., 2010 | HM622595 | HM622601 | Spain: Fresnedilla (Jaén) |
|  | ID504 | 1 | Pedraza-Lara et al., 2010 | HM622595 | HM622602 | Spain: Fresnedilla (Jaén) |
| **JA8** | ID505 | 1 | Pedraza-Lara et al., 2010 | HM622595 | HM622601 | Spain: Vadillo (Jaén) |
|  | ID506 | 1 | Pedraza-Lara et al., 2010 | HM622595 | HM622601 | Spain: Vadillo (Jaén) |
|  | ID507 | 1 | Pedraza-Lara et al., 2010 | HM622595 | HM622601 | Spain: Vadillo (Jaén) |
| **JA9** | ID508 | 1 | Pedraza-Lara et al., 2010 | HM622595 | HM622601 | Spain: Linarejos (Jaén) |
|  | ID509 | 1 | Pedraza-Lara et al., 2010 | HM622595 | HM622601 | Spain: Linarejos (Jaén) |
|  | ID510 | 1 | Pedraza-Lara et al., 2010 | HM622595 | HM622601 | Spain: Linarejos (Jaén) |
| **JA10** | ID511 | 1 | Pedraza-Lara et al., 2010 | HM622595 | HM622601 | Spain: Borosa (Jaén) |
|  | ID512 | 1 | Pedraza-Lara et al., 2010 | HM622595 | HM622601 | Spain: Borosa (Jaén) |
|  | ID513 | 1 | Pedraza-Lara et al., 2010 | HM622595 | HM622601 | Spain: Borosa (Jaén) |
| **JA11** | ID514 | 1 | Pedraza-Lara et al., 2010 | HM622595 | HM622601 | Spain: Aguamula (Jaén) |
|  | ID515 | 1 | Pedraza-Lara et al., 2010 | HM622595 | HM622601 | Spain: Aguamula (Jaén) |
|  | ID516 | 1 | Pedraza-Lara et al., 2010 | HM622595 | HM622603 | Spain: Aguamula (Jaén) |
| **JA12** | ID517 | 1 | Pedraza-Lara et al., 2010 | HM622595 | HM622601 | Spain: Coto Rios (Jaén) |
|  | ID518 | 1 | Pedraza-Lara et al., 2010 | HM622595 | HM622601 | Spain: Coto Rios (Jaén) |
|  | ID519 | 1 | Pedraza-Lara et al., 2010 | HM622595 | HM622601 | Spain: Coto Rios (Jaén) |
|  | ID520 | 1 | Pedraza-Lara et al., 2010 | HM622595 | HM622603 | Spain: Coto Rios (Jaén) |
| **JA13** | ID521 | 1 | Pedraza-Lara et al., 2010 | HM622595 | HM622601 | Spain: Cazorla (Jaén) |
|  | ID522 | 1 | Pedraza-Lara et al., 2010 | HM622595 | HM622601 | Spain: Cazorla (Jaén) |
|  | ID523 | 1 | Pedraza-Lara et al., 2010 | HM622595 | HM622601 | Spain: Cazorla (Jaén) |
| **JA14** | ID613 | 1 | Pedraza-Lara et al., 2010 | HM622595 | HM622601 | Spain: Valdepenas (Jaén) |
|  | ID614 | 1 | Pedraza-Lara et al., 2010 | HM622595 | HM622601 | Spain: Valdepenas (Jaén) |
|  | ID615 | 1 | Pedraza-Lara et al., 2010 | HM622595 | HM622601 | Spain: Valdepenas (Jaén) |
| **JA15** | ID628 | 1 | Pedraza-Lara et al., 2010 | HM622595 | HM622601 | Spain: Vites (Jaén) |
|  | ID629 | 1 | Pedraza-Lara et al., 2010 | HM622595 | HM622601 | Spain: Vites (Jaén) |
|  | ID630 | 1 | Pedraza-Lara et al., 2010 | HM622595 | HM622601 | Spain: Vites (Jaén) |
| **LE1** | LE1_1 | 1, 2 | Matallanas et al., 2016 | JF430574 | FJ897840 | Spain: Lugán (León) |
|  | LE1_2 | 1, 2 | Matallanas et al., 2016 | EF489427 | EF485041 | Spain: Lugán (León) |
|  | LE1_3 | 1, 2 | Matallanas et al., 2016 | EF489427 | EF485041 | Spain: Lugán (León) |
|  | LE1_4 | 1, 2 | Matallanas et al., 2016 | EF489427 | EF485041 | Spain: Lugán (León) |
|  | LE1_5 | 1, 2 | Matallanas et al., 2016 | EF489427 | EF485041 | Spain: Lugán (León) |
|  | LE1_6 | 1, 2 | Matallanas et al., 2016 | EF489427 | EF485041 | Spain: Lugán (León) |
|  | LE1_7 | 1, 2 | Matallanas et al., 2016 | EF489427 | EF485041 | Spain: Lugán (León) |
|  | LE1_8 | 1, 2 | Matallanas et al., 2016 | EF489427 | FJ897843 | Spain: Lugán (León) |
|  | LE1_9 | 1, 2 | Matallanas et al., 2016 | EF489427 | FJ897843 | Spain: Lugán (León) |
|  | LE1_10 | 1, 2 | Matallanas et al., 2016 | EF489427 | FJ897843 | Spain: Lugán (León) |
| **LE3** | LE3_1 | 1, 2 | Martín-Torrijos et al., 2021 | MW327264 | MW325428 | Spain: Garrafe de Torios (León) |
|  | LE3_10 | 1, 2 | Martín-Torrijos et al., 2021 | MW327357 | MW325367 | Spain: Garrafe de Torios (León) |
|  | LE3_11 | 1, 2 | Martín-Torrijos et al., 2021 | MW327358 | MW325432 | Spain: Garrafe de Torios (León) |
|  | LE3_2 | 1, 2 | Martín-Torrijos et al., 2021 | MW327265 | MW325414 | Spain: Garrafe de Torios (León) |
|  | LE3_3 | 1, 2 | Martín-Torrijos et al., 2021 | MW327372 | MW325429 | Spain: Garrafe de Torios (León) |
|  | LE3_4 | 1, 2 | Martín-Torrijos et al., 2021 | MW327373 | MW325431 | Spain: Garrafe de Torios (León) |
|  | LE3_5 | 1, 2 | Martín-Torrijos et al., 2021 | MW327374 | MW325415 | Spain: Garrafe de Torios (León) |
|  | LE3_6 | 1, 2 | Martín-Torrijos et al., 2021 | MW327356 | MW325364 | Spain: Garrafe de Torios (León) |
|  | LE3_7 | 1, 2 | Martín-Torrijos et al., 2021 | MW327362 | MW325365 | Spain: Garrafe de Torios (León) |
|  | LE3_8 | 1, 2 | Martín-Torrijos et al., 2021 | MW327363 | MW325449 | Spain: Garrafe de Torios (León) |
|  | LE3_9 | 1, 2 | Martín-Torrijos et al., 2021 | MW327364 | MW325366 | Spain: Garrafe de Torios (León) |
| **LER1** | LER1_1 | 1, 2 | Matallanas et al., 2016 | JF430574 | FJ897841 | Spain: Pont de Suert (Lleida) |
|  | LER1_2 | 1, 2 | Matallanas et al., 2016 | JF430574 | FJ897841 | Spain: Pont de Suert (Lleida) |
|  | LER1_3 | 1, 2 | Matallanas et al., 2016 | JF430574 | FJ897844 | Spain: Pont de Suert (Lleida) |
|  | LER1_4 | 1, 2 | Matallanas et al., 2016 | JF430574 | FJ897844 | Spain: Pont de Suert (Lleida) |
|  | LER1_5 | 1, 2 | Matallanas et al., 2016 | JF430574 | FJ897844 | Spain: Pont de Suert (Lleida) |
|  | LER1_6 | 1, 2 | Matallanas et al., 2016 | JF430574 | FJ897844 | Spain: Pont de Suert (Lleida) |
|  | LER1_7 | 1, 2 | Matallanas et al., 2016 | JF430574 | FJ897844 | Spain: Pont de Suert (Lleida) |
|  | LER1_8 | 1, 2 | Matallanas et al., 2016 | JF430574 | FJ897844 | Spain: Pont de Suert (Lleida) |
|  | LER1_9 | 1, 2 | Matallanas et al., 2016 | JF430575 | FJ897844 | Spain: Pont de Suert (Lleida) |
|  | LER1_10 | 1, 2 | Matallanas et al., 2016 | FJ897844 | FJ897844 | Spain: Pont de Suert (Lleida) |
|  | LER1_11 | 1, 2 | Matallanas et al., 2016 | FJ897844 | FJ897844 | Spain: Pont de Suert (Lleida) |
| **LER2** | ID568 | 1 | Pedraza-Lara et al., 2010 | HM622595 | HM622603 | Spain: Pont de Suert (Lleida) |
|  | ID569 | 1 | Pedraza-Lara et al., 2010 | HM622595 | HM622603 | Spain: Pont de Suert (Lleida) |
|  | ID570 | 1 | Pedraza-Lara et al., 2010 | HM622595 | HM622603 | Spain: Pont de Suert (Lleida) |
|  | ID571 | 1 | Pedraza-Lara et al., 2010 | HM622595 | HM622603 | Spain: Pont de Suert (Lleida) |
|  | ID572 | 1 | Pedraza-Lara et al., 2010 | HM622595 | HM622603 | Spain: Pont de Suert (Lleida) |
|  | ID573 | 1 | Pedraza-Lara et al., 2010 | HM622595 | HM622603 | Spain: Pont de Suert (Lleida) |
|  | ID574 | 1 | Pedraza-Lara et al., 2010 | HM622595 | HM622603 | Spain: Pont de Suert (Lleida) |
|  | ID575 | 1 | Pedraza-Lara et al., 2010 | HM622595 | HM622603 | Spain: Pont de Suert (Lleida) |
|  | ID576 | 1 | Pedraza-Lara et al., 2010 | HM622595 | HM622603 | Spain: Pont de Suert (Lleida) |
|  | ID577 | 1 | Pedraza-Lara et al., 2010 | HM622595 | HM622607 | Spain: Pont de Suert (Lleida) |
| **LU1** | LU1_1 | 1, 2 | Matallanas et al., 2016 | JF430574 | FJ897840 | Spain: Pol (Lugo) |
|  | LU1_2 | 1, 2 | Matallanas et al., 2016 | JF430574 | FJ897840 | Spain: Pol (Lugo) |
|  | LU1_3 | 1, 2 | Matallanas et al., 2016 | JF430574 | FJ897840 | Spain: Pol (Lugo) |
|  | LU1_4 | 1, 2 | Matallanas et al., 2016 | JF430574 | FJ897840 | Spain: Pol (Lugo) |
|  | LU1_5 | 1, 2 | Matallanas et al., 2016 | JF430574 | FJ897840 | Spain: Pol (Lugo) |
|  | LU1_6 | 1, 2 | Matallanas et al., 2016 | JF430574 | FJ897840 | Spain: Pol (Lugo) |
|  | LU1_7 | 1, 2 | Matallanas et al., 2016 | JF430574 | FJ897840 | Spain: Pol (Lugo) |
|  | LU1_8 | 1, 2 | Matallanas et al., 2016 | JF430574 | FJ897840 | Spain: Pol (Lugo) |
|  | LU1_9 | 1, 2 | Matallanas et al., 2016 | JF430574 | FJ897840 | Spain: Pol (Lugo) |
|  | LU1_10 | 1, 2 | Matallanas et al., 2016 | JF430577 | FJ897840 | Spain: Pol (Lugo) |
| **LU2** | LU2_1 | 1, 2 | Matallanas et al., 2016 | JF430574 | FJ897840 | Spain: Castro de Rei (Lugo) |
|  | LU2_2 | 1, 2 | Matallanas et al., 2016 | JF430574 | FJ897840 | Spain: Castro de Rei (Lugo) |
|  | LU2_3 | 1, 2 | Matallanas et al., 2016 | JF430574 | FJ897840 | Spain: Castro de Rei (Lugo) |
|  | LU2_4 | 1, 2 | Matallanas et al., 2016 | JF430574 | FJ897840 | Spain: Castro de Rei (Lugo) |
|  | LU2_5 | 1, 2 | Matallanas et al., 2016 | JF430574 | FJ897840 | Spain: Castro de Rei (Lugo) |
|  | LU2_6 | 1, 2 | Matallanas et al., 2016 | JF430574 | FJ897840 | Spain: Castro de Rei (Lugo) |
|  | LU2_7 | 1, 2 | Matallanas et al., 2016 | JF430574 | FJ897840 | Spain: Castro de Rei (Lugo) |
|  | LU2_8 | 1, 2 | Matallanas et al., 2016 | JF430574 | FJ897840 | Spain: Castro de Rei (Lugo) |
|  | LU2_9 | 1, 2 | Matallanas et al., 2016 | JF430574 | FJ897840 | Spain: Castro de Rei (Lugo) |
|  | LU2_10 | 1, 2 | Matallanas et al., 2016 | JF430574 | FJ897840 | Spain: Castro de Rei (Lugo) |
| **MA1** | MA1_1 | 1, 2 | In this study | OR195247 | OR197456 | Spain: Guadalevín (Málaga) |
|  | MA1_2 | 1, 2 | In this study | OR195248 | OR197457 | Spain: Guadalevín (Málaga) |
|  | MA1_3 | 1, 2 | In this study | OR195249 | OR197458 | Spain: Guadalevín (Málaga) |
|  | MA1_4 | 1, 2 | In this study | OR195250 | OR197459 | Spain: Guadalevín (Málaga) |
|  | MA1_5 | 1, 2 | In this study | OR195251 | OR197460 | Spain: Guadalevín (Málaga) |
| **MA2** | MA2_1 | 1, 2 | In this study | OR195252 | OR197461 | Spain: Turón (Málaga) |
|  | MA2_2 | 1, 2 | In this study | OR195253 | OR197462 | Spain: Turón (Málaga) |
|  | MA2_3 | 1, 2 | In this study | OR195254 | OR197463 | Spain: Turón (Málaga) |
|  | MA2_4 | 1, 2 | In this study | OR195255 | OR197464 | Spain: Turón (Málaga) |
|  | MA2_5 | 1, 2 | In this study | OR195256 | OR197465 | Spain: Turón (Málaga) |
| **MA3** | MA3_2 | 1, 2 | In this study | OR195257 | OR197466 | Spain: Guadares (Málaga) |
|  | MA3_3 | 1, 2 | In this study | OR195258 | OR197467 | Spain: Guadares (Málaga) |
|  | MA3_4 | 1, 2 | In this study | OR195259 | OR197468 | Spain: Guadares (Málaga) |
|  | MA3_5 | 1, 2 | In this study | OR195260 | OR197469 | Spain: Guadares (Málaga) |
|  | MA3_6 | 1, 2 | In this study | OR195261 | OR197470 | Spain: Guadares (Málaga) |
|  | MA3_7 | 1, 2 | In this study | OR195262 | OR197471 | Spain: Guadares (Málaga) |
| **MA4** | ID616 | 1 | Pedraza-Lara et al., 2010 | HM622595 | HM622601 | Spain: Montejaque (Málaga) |
|  | ID617 | 1 | Pedraza-Lara et al., 2010 | HM622595 | HM622601 | Spain: Montejaque (Málaga) |
|  | ID618 | 1 | Pedraza-Lara et al., 2010 | HM622595 | HM622605 | Spain: Montejaque (Málaga) |
| **MA5** | ID619 | 1 | Pedraza-Lara et al., 2010 | HM622595 | HM622601 | Spain: Ronda (Málaga) |
|  | ID620 | 1 | Pedraza-Lara et al., 2010 | HM622595 | HM622601 | Spain: Ronda (Málaga) |
|  | ID621 | 1 | Pedraza-Lara et al., 2010 | HM622595 | HM622601 | Spain: Ronda (Málaga) |
| **MA6** | ID622 | 1 | Pedraza-Lara et al., 2010 | HM622595 | HM622601 | Spain: Yunquera (Málaga) |
|  | ID623 | 1 | Pedraza-Lara et al., 2010 | HM622595 | HM622601 | Spain: Yunquera (Málaga) |
|  | ID624 | 1 | Pedraza-Lara et al., 2010 | HM622595 | HM622601 | Spain: Yunquera (Málaga) |
| **MA7** | ID625 | 1 | Pedraza-Lara et al., 2010 | HM622595 | HM622601 | Spain: Alcaucin (Málaga) |
|  | ID626 | 1 | Pedraza-Lara et al., 2010 | HM622595 | HM622601 | Spain: Alcaucin (Málaga) |
|  | ID627 | 1 | Pedraza-Lara et al., 2010 | HM622595 | HM622601 | Spain: Alcaucin (Málaga) |
| **MAD1** | MAD_1 | 1, 2 | In this study | OR195263 | OR197472 | Spain: Río Lozoya (Madrid) |
|  | MAD_2 | 1, 2 | In this study | OR195264 | OR197473 | Spain: Río Lozoya (Madrid) |
|  | MAD_3 | 1, 2 | In this study | OR195265 | OR197474 | Spain: Río Lozoya (Madrid) |
| **MON1** | ID54 | 1 | Jelić et al., 2016 | KX370142 | KX369722 | Montenegro: Zeta River |
|  | ID55 | 1 | Jelić et al., 2016 | KX370143 | KX369723 | Montenegro: Zeta River |
|  | ID56 | 1 | Jelić et al., 2016 | KX370144 | KX369724 | Montenegro: Zeta River |
|  | ID57 | 1 | Jelić et al., 2016 | KX370145 | KX369725 | Montenegro: Zeta River |
|  | ID58 | 1 | Jelić et al., 2016 | KX370146 | KX369726 | Montenegro: Zeta River |
|  | ID59 | 1 | Jelić et al., 2016 | KX370147 | KX369727 | Montenegro: Zeta River |
|  | ID60 | 1 | Jelić et al., 2016 | KX370148 | KX369728 | Montenegro: Zeta River |
|  | ID61 | 1 | Jelić et al., 2016 | KX370149 | KX369729 | Montenegro: Zeta River |
|  | ID62 | 1 | Jelić et al., 2016 | KX370150 | KX369730 | Montenegro: Zeta River |
|  | ID63 | 1 | Jelić et al., 2016 | KX370151 | KX369731 | Montenegro: Zeta River |
| **NA2** | NA2_1 | 1, 2 | Martín-Torrijos et al., 2021 | MW327415 | MW325565 | Spain: Doneztebe (Navarra) |
|  | NA2_2 | 1, 2 | Martín-Torrijos et al., 2021 | MW327444 | MW325550 | Spain: Doneztebe (Navarra) |
|  | NA2_3 | 1, 2 | Martín-Torrijos et al., 2021 | MW327445 | MW325569 | Spain: Doneztebe (Navarra) |
| **NA3** | NA3_1 | 1, 2 | Martín-Torrijos et al., 2021 | MW327417 | MW325548 | Spain: Artanga (Navarra) |
|  | NA3_2 | 1, 2 | Martín-Torrijos et al., 2021 | MW327418 | MW325549 | Spain: Artanga (Navarra) |
|  | NA3_3 | 1, 2 | Martín-Torrijos et al., 2021 | MW327255 | MW325479 | Spain: Artanga (Navarra) |
| **NA4** | NA4_1 | 1, 2 | Martín-Torrijos et al., 2021 | MW327393 | MW325539 | Spain: Ultzama-Araquil (Navarra) |
|  | NA4_2 | 1, 2 | Martín-Torrijos et al., 2021 | MW327387 | MW325545 | Spain: Ultzama-Araquil (Navarra) |
|  | NA4_3 | 1, 2 | Martín-Torrijos et al., 2021 | MW327388 | MW325546 | Spain: Ultzama-Araquil (Navarra) |
|  | NA4_5 | 1, 2 | Martín-Torrijos et al., 2021 | MW327446 | MW325547 | Spain: Ultzama-Araquil (Navarra) |
| **NA5** | NA5_2 | 1, 2 | Martín-Torrijos et al., 2021 | MW327389 | MW325540 | Spain: Leurtza (Navarra) |
|  | NA5_3 | 1, 2 | Martín-Torrijos et al., 2021 | MW327390 | MW325541 | Spain: Leurtza (Navarra) |
|  | NA5_4 | 1, 2 | Martín-Torrijos et al., 2021 | MW327391 | MW325542 | Spain: Leurtza (Navarra) |
|  | NA5_5 | 1, 2 | Martín-Torrijos et al., 2021 | MW327392 | MW325568 | Spain: Leurtza (Navarra) |
| **NA7** | NA7_1 | 1, 2 | Martín-Torrijos et al., 2021 | MW327430 | MW327430 | Spain: Sunbilla (Navarra) |
|  | NA7_2 | 1, 2 | Martín-Torrijos et al., 2021 | MW327431 | MW325570 | Spain: Sunbilla (Navarra) |
| **NA8** | NA8_1 | 1, 2 | Matallanas et al., 2016 | EF489427 | EF485041 | Spain: Estella (Navarra) |
|  | NA8_2 | 1, 2 | Matallanas et al., 2016 | EF489427 | EF485041 | Spain: Estella (Navarra) |
|  | NA8_3 | 1, 2 | Matallanas et al., 2016 | EF489427 | EF485041 | Spain: Estella (Navarra) |
|  | NA8_4 | 1, 2 | Matallanas et al., 2016 | EF489427 | EF485041 | Spain: Estella (Navarra) |
|  | NA8_5 | 1, 2 | Matallanas et al., 2016 | EF489427 | EF485041 | Spain: Estella (Navarra) |
|  | NA8_6 | 1, 2 | Matallanas et al., 2016 | EF489427 | EF485041 | Spain: Estella (Navarra) |
|  | NA8_7 | 1, 2 | Matallanas et al., 2016 | EF489427 | EF485041 | Spain: Estella (Navarra) |
|  | NA8_8 | 1, 2 | Matallanas et al., 2016 | EF489427 | EF485041 | Spain: Estella (Navarra) |
|  | NA8_9 | 1, 2 | Matallanas et al., 2016 | EF489427 | EF485041 | Spain: Estella (Navarra) |
|  | NA8_10 | 1, 2 | Matallanas et al., 2016 | JF430574 | FJ897845 | Spain: Estella (Navarra) |
| **PA1** | PA1_1 | 1, 2 | Martín-Torrijos et al., 2021 | MW327262 | MW325515 | Spain: Herrera de Pisuerga (Palencia) |
|  | PA1_2 | 1, 2 | Martín-Torrijos et al., 2021 | MW327407 | MW325597 | Spain: Herrera de Pisuerga (Palencia) |
|  | PA1_3 | 1, 2 | Martín-Torrijos et al., 2021 | MW327300 | MW325516 | Spain: Herrera de Pisuerga (Palencia) |
|  | PA1_4 | 1, 2 | Martín-Torrijos et al., 2021 | MW327419 | MW325598 | Spain: Herrera de Pisuerga (Palencia) |
| **RI1** | ID551 | 1 | Pedraza-Lara et al., 2010 | HM622595 | HM622601 | Spain: Rivabellosa (La Rioja) |
|  | ID552 | 1 | Pedraza-Lara et al., 2010 | HM622595 | HM622601 | Spain: Rivabellosa (La Rioja) |
|  | ID553 | 1 | Pedraza-Lara et al., 2010 | HM622595 | HM622601 | Spain: Rivabellosa (La Rioja) |
| **RI2** | ID554 | 1 | Pedraza-Lara et al., 2010 | HM622595 | HM622601 | Spain: El Rasillo (La Rioja) |
|  | ID555 | 1 | Pedraza-Lara et al., 2010 | HM622595 | HM622601 | Spain: El Rasillo (La Rioja) |
|  | ID556 | 1 | Pedraza-Lara et al., 2010 | HM622595 | HM622601 | Spain: El Rasillo (La Rioja) |
| **RI3** | ID557 | 1 | Pedraza-Lara et al., 2010 | HM622595 | HM622601 | Spain: Leza (La Rioja) |
|  | ID558 | 1 | Pedraza-Lara et al., 2010 | HM622595 | HM622601 | Spain: Leza (La Rioja) |
|  | ID559 | 1 | Pedraza-Lara et al., 2010 | HM622595 | HM622601 | Spain: Leza (La Rioja) |
| **RI4** | ID563 | 1 | Pedraza-Lara et al., 2010 | HM622595 | HM622601 | Spain: Faldetores (La Rioja) |
|  | ID564 | 1 | Pedraza-Lara et al., 2010 | HM622595 | HM622601 | Spain: Faldetores (La Rioja) |
|  | ID565 | 1 | Pedraza-Lara et al., 2010 | HM622595 | HM622601 | Spain: Faldetores (La Rioja) |
| **SLO1** | ID163 | 1 | Jelić et al., 2016 | KX370251 | KX369831 | Slovenia: Rižana |
|  | ID164 | 1 | Jelić et al., 2016 | KX370252 | KX369832 | Slovenia: Rižana |
|  | ID165 | 1 | Jelić et al., 2016 | KX370253 | KX369833 | Slovenia: Rižana |
|  | ID166 | 1 | Jelić et al., 2016 | KX370254 | KX369834 | Slovenia: Rižana |
|  | ID167 | 1 | Jelić et al., 2016 | KX370255 | KX369835 | Slovenia: Rižana |
| **SO1** | SO1_1 | 1, 2 | Martín-Torrijos et al., 2021 | MW327341 | MW325506 | Spain: Navaceno (Soria) |
|  | SO1_11 | 1, 2 | Martín-Torrijos et al., 2021 | MW327248 | MW325454 | Spain: Navaceno (Soria) |
|  | SO1_2 | 1, 2 | Martín-Torrijos et al., 2021 | MW327342 | MW325518 | Spain: Navaceno (Soria) |
|  | SO1_3 | 1, 2 | Martín-Torrijos et al., 2021 | MW327365 | MW325520 | Spain: Navaceno (Soria) |
|  | SO1_4 | 1, 2 | Martín-Torrijos et al., 2021 | MW327322 | MW325519 | Spain: Navaceno (Soria) |
|  | SO1_5 | 1, 2 | Martín-Torrijos et al., 2021 | MW327321 | MW325524 | Spain: Navaceno (Soria) |
|  | SO1_6 | 1, 2 | Martín-Torrijos et al., 2021 | MW327246 | MW325507 | Spain: Navaceno (Soria) |
|  | SO1_7 | 1, 2 | Martín-Torrijos et al., 2021 | MW327247 | MW325508 | Spain: Navaceno (Soria) |
|  | SO1_8 | 1, 2 | Martín-Torrijos et al., 2021 | MW327297 | MW325509 | Spain: Navaceno (Soria) |
|  | SO1_9 | 1, 2 | Martín-Torrijos et al., 2021 | MW327249 | MW325403 | Spain: Navaceno (Soria) |
| **SO2** | SO2_1 | 1, 2 | Martín-Torrijos et al., 2021 | MW327367 | MW325521 | Spain: Navaceno (Soria) |
|  | SO2_2 | 1, 2 | Martín-Torrijos et al., 2021 | MW327366 | MW325522 | Spain: Navaceno (Soria) |
|  | SO2_3 | 1, 2 | Martín-Torrijos et al., 2021 | MW327320 | MW325439 | Spain: Navaceno (Soria) |
|  | SO2_4 | 1, 2 | Martín-Torrijos et al., 2021 | MW327323 | MW325440 | Spain: Navaceno (Soria) |
|  | SO2_5 | 1, 2 | Martín-Torrijos et al., 2021 | MW327324 | MW325430 | Spain: Navaceno (Soria) |
|  | SO2_6 | 1, 2 | Martín-Torrijos et al., 2021 | MW327309 | MW325476 | Spain: Navaceno (Soria) |
|  | SO2_7 | 1, 2 | Martín-Torrijos et al., 2021 | MW327311 | MW325477 | Spain: Navaceno (Soria) |
|  | SO2_8 | 1, 2 | Martín-Torrijos et al., 2021 | MW327315 | MW325478 | Spain: Navaceno (Soria) |
|  | SO2_9 | 1, 2 | Martín-Torrijos et al., 2021 | MW327310 | MW325475 | Spain: Navaceno (Soria) |
| **SO3** | SO3_4 | 1, 2 | Martín-Torrijos et al., 2021 | MW327226 | MW325473 | Spain: Mont Vicarias (Soria) |
|  | SO3_5 | 1, 2 | Martín-Torrijos et al., 2021 | MW327227 | MW325397 | Spain: Mont Vicarias (Soria) |
| **SO8** | SO8_1 | 1, 2 | Martín-Torrijos et al., 2021 | MW327273 | MW325413 | Spain: Almarza (Soria) |
|  | SO8_10 | 1, 2 | Martín-Torrijos et al., 2021 | MW327259 | MW325469 | Spain: Almarza (Soria) |
|  | SO8_2 | 1, 2 | Martín-Torrijos et al., 2021 | MW327299 | MW325359 | Spain: Almarza (Soria) |
|  | SO8_3 | 1, 2 | Martín-Torrijos et al., 2021 | MW327256 | MW325360 | Spain: Almarza (Soria) |
|  | SO8_4 | 1, 2 | Martín-Torrijos et al., 2021 | MW327260 | MW325517 | Spain: Almarza (Soria) |
|  | SO8_5 | 1, 2 | Martín-Torrijos et al., 2021 | MW327370 | MW325448 | Spain: Almarza (Soria) |
|  | SO8_6 | 1, 2 | Martín-Torrijos et al., 2021 | MW327257 | MW325361 | Spain: Almarza (Soria) |
|  | SO8_7 | 1, 2 | Martín-Torrijos et al., 2021 | MW327314 | MW325363 | Spain: Almarza (Soria) |
|  | SO8_9 | 1, 2 | Martín-Torrijos et al., 2021 | MW327258 | MW325362 | Spain: Almarza (Soria) |
| **SO15** | SO15_1 | 1, 2 | Martín-Torrijos et al., 2021 | MW327396 | MW325533 | Spain: Devanos (Soria) |
|  | SO15_10 | 1, 2 | Martín-Torrijos et al., 2021 | MW327400 | MW325538 | Spain: Devanos (Soria) |
|  | SO15_2 | 1, 2 | Martín-Torrijos et al., 2021 | MW327421 | MW325534 | Spain: Devanos (Soria) |
|  | SO15_3 | 1, 2 | Martín-Torrijos et al., 2021 | MW327422 | MW325559 | Spain: Devanos (Soria) |
|  | SO15_4 | 1, 2 | Martín-Torrijos et al., 2021 | MW327401 | MW325560 | Spain: Devanos (Soria) |
|  | SO15_5 | 1, 2 | Martín-Torrijos et al., 2021 | MW327433 | MW325561 | Spain: Devanos (Soria) |
|  | SO15_6 | 1, 2 | Martín-Torrijos et al., 2021 | MW327397 | MW325535 | Spain: Devanos (Soria) |
|  | SO15_8 | 1, 2 | Martín-Torrijos et al., 2021 | MW327398 | MW325536 | Spain: Devanos (Soria) |
|  | SO15_9 | 1, 2 | Martín-Torrijos et al., 2021 | MW327399 | MW325537 | Spain: Devanos (Soria) |
| **TAR1** | ID590 | 1 | Pedraza-Lara et al., 2010 | HM622595 | HM622603 | Spain: Montsant (Tarragona) |
|  | ID591 | 1 | Pedraza-Lara et al., 2010 | HM622595 | HM622603 | Spain: Montsant (Tarragona) |
|  | ID592 | 1 | Pedraza-Lara et al., 2010 | HM622595 | HM622603 | Spain: Montsant (Tarragona) |
|  | ID593 | 1 | Pedraza-Lara et al., 2010 | HM622595 | HM622603 | Spain: Montsant (Tarragona) |
| **TAR2** | ID594 | 1 | Pedraza-Lara et al., 2010 | HM622595 | HM622603 | Spain: Moli de l Esquirola (Tarragona) |
|  | ID595 | 1 | Pedraza-Lara et al., 2010 | HM622595 | HM622603 | Spain: Moli de l Esquirola (Tarragona) |
|  | ID596 | 1 | Pedraza-Lara et al., 2010 | HM622595 | HM622603 | Spain: Moli de l Esquirola (Tarragona) |
| **TAR3** | ID597 | 1 | Pedraza-Lara et al., 2010 | HM622595 | HM622601 | Spain: Ports de Beseit (Tarragona) |
|  | ID598 | 1 | Pedraza-Lara et al., 2010 | HM622595 | HM622601 | Spain: Ports de Beseit (Tarragona) |
|  | ID599 | 1 | Pedraza-Lara et al., 2010 | HM622595 | HM622601 | Spain: Ports de Beseit (Tarragona) |
|  | ID600 | 1 | Pedraza-Lara et al., 2010 | HM622595 | HM622603 | Spain: Ports de Beseit (Tarragona) |
|  | ID601 | 1 | Pedraza-Lara et al., 2010 | HM622595 | HM622603 | Spain: Ports de Beseit (Tarragona) |
|  | ID602 | 1 | Pedraza-Lara et al., 2010 | HM622595 | HM622603 | Spain: Ports de Beseit (Tarragona) |
|  | ID603 | 1 | Pedraza-Lara et al., 2010 | HM622595 | HM622603 | Spain: Ports de Beseit (Tarragona) |
|  | ID604 | 1 | Pedraza-Lara et al., 2010 | HM622595 | HM622603 | Spain: Ports de Beseit (Tarragona) |
|  | ID605 | 1 | Pedraza-Lara et al., 2010 | HM622595 | HM622603 | Spain: Ports de Beseit (Tarragona) |
|  | ID606 | 1 | Pedraza-Lara et al., 2010 | HM622595 | HM622603 | Spain: Ports de Beseit (Tarragona) |
| **TE1** | TE1_7 | 1, 2 | Martín-Torrijos et al., 2021 | MW327369 | MW325347 | Spain: Valderrobles (Teruel) |
|  | TE1_8 | 1, 2 | Martín-Torrijos et al., 2021 | MW327261 | MW325348 | Spain: Valderrobles (Teruel) |
| **TE2** | TE2_1 | 1, 2 | Matallanas et al., 2016 | JF430574 | FJ897840 | Spain: Beceite (Teruel) |
|  | TE2_2 | 1, 2 | Matallanas et al., 2016 | JF430574 | FJ897840 | Spain: Beceite (Teruel) |
|  | TE2_3 | 1, 2 | Matallanas et al., 2016 | JF430574 | FJ897840 | Spain: Beceite (Teruel) |
|  | TE2_4 | 1, 2 | Matallanas et al., 2016 | JF430574 | FJ897840 | Spain: Beceite (Teruel) |
|  | TE2_5 | 1, 2 | Matallanas et al., 2016 | JF430574 | FJ897840 | Spain: Beceite (Teruel) |
|  | TE2_6 | 1, 2 | Matallanas et al., 2016 | JF430574 | FJ897840 | Spain: Beceite (Teruel) |
|  | TE2_7 | 1, 2 | Matallanas et al., 2016 | JF430574 | FJ897840 | Spain: Beceite (Teruel) |
|  | TE2_8 | 1, 2 | Matallanas et al., 2016 | JF430574 | FJ897840 | Spain: Beceite (Teruel) |
|  | TE2_9 | 1, 2 | Matallanas et al., 2016 | JF430574 | FJ897840 | Spain: Beceite (Teruel) |
|  | TE2_10 | 1, 2 | Matallanas et al., 2016 | JF430574 | FJ897840 | Spain: Beceite (Teruel) |
| **TE3** | TE3_1 | 1, 2 | Matallanas et al., 2016 | JF430574 | FJ897840 | Spain: Castellote (Teruel) |
|  | TE3_2 | 1, 2 | Matallanas et al., 2016 | JF430574 | FJ897840 | Spain: Castellote (Teruel) |
|  | TE3_3 | 1, 2 | Matallanas et al., 2016 | JF430574 | FJ897840 | Spain: Castellote (Teruel) |
|  | TE3_4 | 1, 2 | Matallanas et al., 2016 | JF430574 | FJ897840 | Spain: Castellote (Teruel) |
|  | TE3_5 | 1, 2 | Matallanas et al., 2016 | JF430574 | FJ897840 | Spain: Castellote (Teruel) |
|  | TE3_6 | 1, 2 | Matallanas et al., 2016 | JF430574 | FJ897840 | Spain: Castellote (Teruel) |
|  | TE3_7 | 1, 2 | Matallanas et al., 2016 | JF430574 | FJ897840 | Spain: Castellote (Teruel) |
|  | TE3_8 | 1, 2 | Matallanas et al., 2016 | JF430574 | FJ897841 | Spain: Castellote (Teruel) |
|  | TE3_9 | 1, 2 | Matallanas et al., 2016 | JF430574 | FJ897841 | Spain: Castellote (Teruel) |
|  | TE3_10 | 1, 2 | Matallanas et al., 2016 | JF430574 | FJ897841 | Spain: Castellote (Teruel) |
| **TE5** | TE5_1 | 1, 2 | Matallanas et al., 2016 | JF430574 | FJ897840 | Spain: Cucalón (Teruel) |
|  | TE5_2 | 1, 2 | Matallanas et al., 2016 | JF430574 | FJ897840 | Spain: Cucalón (Teruel) |
|  | TE5_3 | 1, 2 | Matallanas et al., 2016 | JF430574 | FJ897840 | Spain: Cucalón (Teruel) |
|  | TE5_4 | 1, 2 | Matallanas et al., 2016 | JF430574 | FJ897840 | Spain: Cucalón (Teruel) |
|  | TE5_5 | 1, 2 | Matallanas et al., 2016 | JF430577 | FJ897840 | Spain: Cucalón (Teruel) |
|  | TE5_6 | 1, 2 | Matallanas et al., 2016 | JF430574 | FJ897841 | Spain: Cucalón (Teruel) |
|  | TE5_7 | 1, 2 | Matallanas et al., 2016 | JF430574 | FJ897841 | Spain: Cucalón (Teruel) |
|  | TE5_8 | 1, 2 | Matallanas et al., 2016 | JF430574 | FJ897841 | Spain: Cucalón (Teruel) |
|  | TE5_9 | 1, 2 | Matallanas et al., 2016 | JF430579 | FJ897841 | Spain: Cucalón (Teruel) |
|  | TE5_10 | 1, 2 | Matallanas et al., 2016 | JF430580 | FJ897843 | Spain: Cucalón (Teruel) |
| **TE6** | TE6_1 | 1, 2 | In this study | OR195266 | OR197475 | Spain: Loscos (Teruel) |
|  | TE6_2 | 1, 2 | In this study | OR195267 | OR197476 | Spain: Loscos (Teruel) |
|  | TE6_4 | 1, 2 | In this study | OR195268 | OR197477 | Spain: Loscos (Teruel) |
|  | TE6_5 | 1, 2 | In this study | OR195269 | OR197478 | Spain: Loscos (Teruel) |
| **TE7** | TE7_1 | 1, 2 | In this study | OR195270 | OR197479 | Spain: Villaroya de los Pinares (Teruel) |
|  | TE7_4 | 1, 2 | In this study | OR195271 | OR197480 | Spain: Villaroya de los Pinares (Teruel) |
|  | TE7_5 | 1, 2 | In this study | OR195272 | OR197481 | Spain: Villaroya de los Pinares (Teruel) |
| **TE8** | TE8_1 | 1, 2 | In this study | OR195273 | OR197482 | Spain: Fortanete (Teruel) |
|  | TE8_5 | 1, 2 | In this study | OR195274 | OR197483 | Spain: Fortanete (Teruel) |
| **TE9** | TE9_1 | 1, 2 | In this study | OR195275 | OR197484 | Spain: Bordón (Teruel) |
|  | TE9_2 | 1, 2 | In this study | OR195276 | OR197485 | Spain: Bordón (Teruel) |
|  | TE9_3 | 1, 2 | In this study | OR195277 | OR197486 | Spain: Bordón (Teruel) |
|  | TE9_5 | 1, 2 | In this study | OR195278 | OR197487 | Spain: Bordón (Teruel) |
| **TE10** | TE10_2 | 1, 2 | In this study | OR195279 | OR197488 | Spain: Castillote (Teruel) |
|  | TE10_3 | 1, 2 | In this study | OR195280 | OR197489 | Spain: Castillote (Teruel) |
|  | TE10_4 | 1, 2 | In this study | OR195281 | OR197490 | Spain: Castillote (Teruel) |
|  | TE10_5 | 1, 2 | In this study | OR195282 | OR197491 | Spain: Castillote (Teruel) |
| **TE11** | TE11_1 | 1, 2 | In this study | OR195283 | OR197492 | Spain: Villarluengo (Teruel) |
|  | TE11_2 | 1, 2 | In this study | OR195284 | OR197493 | Spain: Villarluengo (Teruel) |
|  | TE11_3 | 1, 2 | In this study | OR195285 | OR197494 | Spain: Villarluengo (Teruel) |
|  | TE11_4 | 1, 2 | In this study | OR195286 | OR197495 | Spain: Villarluengo (Teruel) |
|  | TE11_5 | 1, 2 | In this study | OR195287 | OR197496 | Spain: Villarluengo (Teruel) |
| **TE12** | TE12_1 | 1, 2 | In this study | OR195288 | OR197497 | Spain: Rillo - Son del Puerto (Teruel) |
|  | TE12_2 | 1, 2 | In this study | OR195289 | OR197498 | Spain: Rillo - Son del Puerto (Teruel) |
|  | TE12_3 | 1, 2 | In this study | OR195290 | OR197499 | Spain: Rillo - Son del Puerto (Teruel) |
|  | TE12_4 | 1, 2 | In this study | OR195291 | OR197500 | Spain: Rillo - Son del Puerto (Teruel) |
|  | TE12_5 | 1, 2 | In this study | OR195292 | OR197501 | Spain: Rillo - Son del Puerto (Teruel) |
| **TE13** | TE13_1 | 1, 2 | In this study | OR195293 | OR197502 | Spain: Beceite (Teruel) |
|  | TE13_2 | 1, 2 | In this study | OR195294 | OR197503 | Spain: Beceite (Teruel) |
|  | TE13_3 | 1, 2 | In this study | OR195295 | OR197504 | Spain: Beceite (Teruel) |
|  | TE13_4 | 1, 2 | In this study | OR195296 | OR197505 | Spain: Beceite (Teruel) |
|  | TE13_5 | 1, 2 | In this study | OR195297 | OR197506 | Spain: Beceite (Teruel) |
| **TE14** | TE14_1 | 1, 2 | In this study | OR195298 | OR197507 | Spain: Riodeva (Teruel) |
|  | TE14_2 | 1, 2 | In this study | OR195299 | OR197508 | Spain: Riodeva (Teruel) |
|  | TE14_3 | 1, 2 | In this study | OR195300 | OR197509 | Spain: Riodeva (Teruel) |
|  | TE14_4 | 1, 2 | In this study | OR195301 | OR197510 | Spain: Riodeva (Teruel) |
|  | TE14_5 | 1, 2 | In this study | OR195302 | OR197511 | Spain: Riodeva (Teruel) |
| **TE15** | TE15_2 | 1, 2 | In this study | OR195303 | OR197512 | Spain: Mora de Rubielos (Teruel) |
|  | TE15_4 | 1, 2 | In this study | OR195304 | OR197513 | Spain: Mora de Rubielos (Teruel) |
|  | TE15_5 | 1, 2 | In this study | OR195305 | OR197514 | Spain: Mora de Rubielos (Teruel) |
|  | TE15_6 | 1, 2 | In this study | OR195306 | OR197515 | Spain: Mora de Rubielos (Teruel) |
|  | ID578 | 1 | Pedraza-Lara et al., 2010 | HM622595 | HM622601 | Spain: Mora de Rubielos (Teruel) |
|  | ID579 | 1 | Pedraza-Lara et al., 2010 | HM622595 | HM622601 | Spain: Mora de Rubielos (Teruel) |
|  | ID580 | 1 | Pedraza-Lara et al., 2010 | HM622595 | HM622601 | Spain: Mora de Rubielos (Teruel) |
| **TE16** | ID581 | 1 | Pedraza-Lara et al., 2010 | HM622595 | HM622601 | Spain: Valhondo (Teruel) |
|  | ID582 | 1 | Pedraza-Lara et al., 2010 | HM622595 | HM622601 | Spain: Valhondo (Teruel) |
|  | ID583 | 1 | Pedraza-Lara et al., 2010 | HM622595 | HM622601 | Spain: Valhondo (Teruel) |
| **TE17** | ID587 | 1 | Pedraza-Lara et al., 2010 | HM622595 | HM622603 | Spain: Amanaderos (Teruel) |
|  | ID588 | 1 | Pedraza-Lara et al., 2010 | HM622595 | HM622603 | Spain: Amanaderos (Teruel) |
|  | ID589 | 1 | Pedraza-Lara et al., 2010 | HM622595 | HM622603 | Spain: Amanaderos (Teruel) |
| **TE18** | ID634 | 1 | Pedraza-Lara et al., 2010 | HM622595 | HM622601 | Spain: Arcos de las Salinas (Teruel) |
|  | ID635 | 1 | Pedraza-Lara et al., 2010 | HM622595 | HM622601 | Spain: Arcos de las Salinas (Teruel) |
|  | ID636 | 1 | Pedraza-Lara et al., 2010 | HM622595 | HM622601 | Spain: Arcos de las Salinas (Teruel) |
| **VA1** | VA1_1 | 1, 2 | Matallanas et al., 2016 | JF430574 | FJ897841 | Spain: Utiel (Valencia) |
|  | VA1_2 | 1, 2 | Matallanas et al., 2016 | JF430574 | FJ897841 | Spain: Utiel (Valencia) |
|  | VA1_3 | 1, 2 | Matallanas et al., 2016 | JF430574 | FJ897841 | Spain: Utiel (Valencia) |
|  | VA1_4 | 1, 2 | Matallanas et al., 2016 | JF430574 | FJ897841 | Spain: Utiel (Valencia) |
|  | VA1_5 | 1, 2 | Matallanas et al., 2016 | JF430574 | FJ897841 | Spain: Utiel (Valencia) |
|  | VA1_6 | 1, 2 | Matallanas et al., 2016 | JF430574 | FJ897841 | Spain: Utiel (Valencia) |
|  | VA1_7 | 1, 2 | Matallanas et al., 2016 | JF430574 | FJ897841 | Spain: Utiel (Valencia) |
|  | VA1_8 | 1, 2 | Matallanas et al., 2016 | JF430574 | FJ897841 | Spain: Utiel (Valencia) |
|  | VA1_9 | 1, 2 | Matallanas et al., 2016 | EF489427 | JF430572 | Spain: Utiel (Valencia) |
|  | VA1_10 | 1, 2 | Matallanas et al., 2016 | EF489427 | JF430572 | Spain: Utiel (Valencia) |
| **VA2** | ID640 | 1 | Pedraza-Lara et al., 2010 | HM622595 | HM622601 | Spain: Titaguas (Valencia) |
|  | ID641 | 1 | Pedraza-Lara et al., 2010 | HM622595 | HM622601 | Spain: Titaguas (Valencia) |
|  | ID642 | 1 | Pedraza-Lara et al., 2010 | HM622595 | HM622601 | Spain: Titaguas (Valencia) |
| **VA3** | ID643 | 1 | Pedraza-Lara et al., 2010 | HM622595 | HM622601 | Spain: Campo de Arriba (Valencia) |
|  | ID644 | 1 | Pedraza-Lara et al., 2010 | HM622595 | HM622601 | Spain: Campo de Arriba (Valencia) |
|  | ID645 | 1 | Pedraza-Lara et al., 2010 | HM622595 | HM622601 | Spain: Campo de Arriba (Valencia) |
| **VALL1** | VALL1_1 | 1, 2 | Martín-Torrijos et al., 2021 | MW327343 | MW325404 | Spain: Adalia (Valladolid) |
|  | VALL1_2 | 1, 2 | Martín-Torrijos et al., 2021 | MW327344 | MW325405 | Spain: Adalia (Valladolid) |
|  | VALL1_3 | 1, 2 | Martín-Torrijos et al., 2021 | MW327345 | MW325406 | Spain: Adalia (Valladolid) |
|  | VALL1_4 | 1, 2 | Martín-Torrijos et al., 2021 | MW327228 | MW325407 | Spain: Adalia (Valladolid) |
|  | VALL1_5 | 1, 2 | Martín-Torrijos et al., 2021 | MW327229 | MW325408 | Spain: Adalia (Valladolid) |
|  | VALL1_6 | 1, 2 | Martín-Torrijos et al., 2021 | MW327230 | MW325409 | Spain: Adalia (Valladolid) |
| **VALL2** | VALL2_10 | 1, 2 | Martín-Torrijos et al., 2021 | MW327231 | MW325346 | Spain: Adalia (Valladolid) |
|  | VALL2_5 | 1, 2 | Martín-Torrijos et al., 2021 | MW327346 | MW325345 | Spain: Adalia (Valladolid) |
| **ZA1** | ZA1_1 | 1, 2 | Matallanas et al., 2016 | JF430574 | FJ897840 | Spain: Santa Eulalia de Gállego (Zaragoza) |
|  | ZA1_2 | 1, 2 | Matallanas et al., 2016 | JF430574 | FJ897840 | Spain: Santa Eulalia de Gállego (Zaragoza) |
|  | ZA1_3 | 1, 2 | Matallanas et al., 2016 | JF430574 | FJ897840 | Spain: Santa Eulalia de Gállego (Zaragoza) |
|  | ZA1_4 | 1, 2 | Matallanas et al., 2016 | JF430574 | FJ897840 | Spain: Santa Eulalia de Gállego (Zaragoza) |
|  | ZA1_5 | 1, 2 | Matallanas et al., 2016 | JF430574 | FJ897840 | Spain: Santa Eulalia de Gállego (Zaragoza) |
|  | ZA1_6 | 1, 2 | Matallanas et al., 2016 | JF430574 | FJ897841 | Spain: Santa Eulalia de Gállego (Zaragoza) |
|  | ZA1_7 | 1, 2 | Matallanas et al., 2016 | JF430574 | FJ897841 | Spain: Santa Eulalia de Gállego (Zaragoza) |
|  | ZA1_8 | 1, 2 | Matallanas et al., 2016 | EF489427 | EF485041 | Spain: Santa Eulalia de Gállego (Zaragoza) |
|  | ZA1_9 | 1, 2 | Matallanas et al., 2016 | EF489427 | EF485041 | Spain: Santa Eulalia de Gállego (Zaragoza) |
|  | ZA1_10 | 1, 2 | Matallanas et al., 2016 | EF489427 | EF485041 | Spain: Santa Eulalia de Gállego (Zaragoza) |
| **ZA2** | ZA2_1 | 1, 2 | In this study | OR195307 | OR197516 | Spain: Uncastillo (Zaragoza) |
|  | ZA2_2 | 1, 2 | In this study | OR195308 | OR197517 | Spain: Uncastillo (Zaragoza) |
|  | ZA2_3 | 1, 2 | In this study | OR195309 | OR197518 | Spain: Uncastillo (Zaragoza) |
| **ZA3** | ZA3_1 | 1, 2 | In this study | OR195310 | OR197519 | Spain: Cervezuela (Zaragoza) |
|  | ZA3_2 | 1, 2 | In this study | OR195311 | OR197520 | Spain: Cervezuela (Zaragoza) |
|  | ZA3_3 | 1, 2 | In this study | OR195312 | OR197521 | Spain: Cervezuela (Zaragoza) |
|  | ZA3_4 | 1, 2 | In this study | OR195313 | OR197522 | Spain: Cervezuela (Zaragoza) |
|  | ZA3_5 | 1, 2 | In this study | OR195314 | OR197523 | Spain: Cervezuela (Zaragoza) |
| **ZA4** | ZA4_1 | 1, 2 | In this study | OR195315 | OR197524 | Spain: Orera (Zaragoza) |
|  | ZA4_2 | 1, 2 | In this study | OR195316 | OR197525 | Spain: Orera (Zaragoza) |
|  | ZA4_3 | 1, 2 | In this study | OR195317 | OR197526 | Spain: Orera (Zaragoza) |
|  | ZA4_4 | 1, 2 | In this study | OR195318 | OR197527 | Spain: Orera (Zaragoza) |
|  | ZA4_5 | 1, 2 | In this study | OR195319 | OR197528 | Spain: Orera (Zaragoza) |
| **ZA5** | ID560 | 1 | Pedraza-Lara et al., 2010 | HM622595 | HM622601 | Spain: Rigobundo (Zaragoza) |
|  | ID561 | 1 | Pedraza-Lara et al., 2010 | HM622595 | HM622601 | Spain: Rigobundo (Zaragoza) |
|  | ID562 | 1 | Pedraza-Lara et al., 2010 | HM622595 | HM622601 | Spain: Rigobundo (Zaragoza) |
| **VIZ2** | ID288 | 1 | Jelić et al., 2016 | KX370376 | KX369956 | Spain: San Sebastian (Vizcaya) |
|  | ID289 | 1 | Jelić et al., 2016 | KX370377 | KX369957 | Spain: San Sebastian (Vizcaya) |
|  | ID290 | 1 | Jelić et al., 2016 | KX370378 | KX369958 | Spain: San Sebastian (Vizcaya) |
|  | ID291 | 1 | Jelić et al., 2016 | KX370379 | KX369959 | Spain: San Sebastian (Vizcaya) |
|  | ID292 | 1 | Jelić et al., 2016 | KX370380 | KX369960 | Spain: San Sebastian (Vizcaya) |
|  | ID293 | 1 | Jelić et al., 2016 | KX370381 | KX369961 | Spain: San Sebastian (Vizcaya) |
|  | ID294 | 1 | Jelić et al., 2016 | KX370382 | KX369962 | Spain: San Sebastian (Vizcaya) |
|  | ID295 | 1 | Jelić et al., 2016 | KX370383 | KX369963 | Spain: San Sebastian (Vizcaya) |
|  | ID296 | 1 | Jelić et al., 2016 | KX370384 | KX369964 | Spain: San Sebastian (Vizcaya) |
