# Supplementary material for: On the conservation of white-clawed crayfish in the Iberian Peninsula: Unraveling its genetic diversity and structure, and origin
Source: PLoS One. 2023 Oct 13;18(10):e0292679. doi: 10.1371/journal.pone.0292679 (PMC10575519; doi:10.1371/journal.pone.0292679)
Supplement: S2 Table — Nucleotides of the obtained sequences of the cytochrome oxidase subunit I region from the GIR5 population in positions with double peaks (on the left) in alignment with the haplotype sequences obtained for dataset 1 (sequences from the whole geographical distribution range, covering all lineages and clades previously defined to the WCC species complex). Dots indicate identity with the consensus sequence, while the variable position that showed double peaks appears in parenthesis. Asterisks indicate nucleotides previously unreported in that position. (DOCX) [file pone.0292679.s008.docx]

**S2 Table**. **Positions (base pair) with double peaks of the cytochrome oxidase subunit I region from the GIR5 population.** Nucleotides of the obtained sequences of the cytochrome oxidase subunit I region from the GIR5 population in positions with double peaks (on the left) in alignment with the haplotype sequences obtained for dataset 1 (sequences from the whole geographical distribution range, covering all lineages and clades previously defined to the WCC species complex). Dots indicate identity with the consensus sequence, while the variable position that showed double peaks appears in parenthesis. Asterisks indicate nucleotides previously unreported in that position.

|  |  | **ID** | | | | | |
| --- | --- | --- | --- | --- | --- | --- | --- |
| **bp position** | | **GIR5_1** | **GIR5_2** | **GIR5_3** | **GIR5_4** | **GIR5_5** | **GIR5_6** |
| **12** | GGG**(C)**AGT | …C/T… | …C/T… | …C… | …C/T… | …C… | …C/T… |
| **27** | GGA**(C)**GAT | …C/T… | …C/T… | …C… | …C/T… | …C… | …A*/T… |
| **108** | GGG**(G)**TTT | …G/A… | …G/A… | …A… | …G/A… | …A… | …G/A… |
| **137** | ATG**(T)**TAG | …C/T… | …C/T… | …T… | …C/T… | …T… | …C… |
| **145** | AGC**(T)**CCT | …C/T… | …C/T… | …C… | …C/T… | …C… | …T… |
| **151** | TGA**(T)**ATG | …C/T… | …C/T… | …C… | …C/T… | …C… | …T… |
| **196** | TCC**(A)**TTT | …G/A… | …G/A… | …G… | …G/A… | …G… | …A… |
| **209** | ACT**(C)**TAT | …C/T… | …C… | …C… | …C/T… | …C… | …T… |
| **241** | GGG**(A)**GTT | …A… | …A… | …A… | …A/G… | …A… | …G… |
| **250** | GAC**(A)**GGG | …A/G… | …A/G… | …A… | …A… | …A… | …A… |
| **253** | AGG**(G)**TGA | …G/T*… | …G/T*… | …G… | …G… | …G… | …G/A… |
| **262** | TGT**(C)**TAC | …C/T… | …C/T… | …C… | …C/T… | …C… | …C/T… |
| **268** | TCC**(T)**CCT | …C… | …C/T… | …C… | …C/T… | …C… | …C/T… |
| **289** | TGC**(T)**CAC | …C/T… | …C/T… | …T… | …C/T… | …T… | …C/T… |
| **298** | AGG**(G)**GCG | …A/G… | …A/G… | …G… | …A/G… | …G… | …A… |
| **519** | TCG**(T)**AAT | …T/G… | …T/G… | …T… | …T/G… | …T… | …G… |
