# Supplementary material for: On the conservation of white-clawed crayfish in the Iberian Peninsula: Unraveling its genetic diversity and structure, and origin
Source: PLoS One. 2023 Oct 13;18(10):e0292679. doi: 10.1371/journal.pone.0292679 (PMC10575519; doi:10.1371/journal.pone.0292679)
Supplement: S3 Table — Genetic diversity indices of the populations from dataset 1 (sequences from the whole geographical distribution range, covering all lineages and clades previously defined to the WCC species complex) based on 948 bp of the concatenated mitochondrial 16S rRNA and cytochrome oxidase subunit I regions: sample size (n), the number of polymorphic sites (S), the number of haplotypes (H), the haplotype diversity (Hd), the nucleotide diversity (π), the Tajima’s D (D), and Fu’s Fs (Fs). (DOCX) [file pone.0292679.s009.docx]

**S3 Table. Dataset 1 genetic diversity indices.** Genetic diversity indices of the populations from dataset 1 (sequences from the whole geographical distribution range, covering all lineages and clades previously defined to the WCC species complex) based on 948 bp of the concatenated mitochondrial 16S rRNA and cytochrome oxidase subunit I regions: sample size (n), the number of polymorphic sites (S), the number of haplotypes (H), the haplotype diversity (Hd), the nucleotide diversity (π), the Tajima’s D (D), and Fu’s Fs (Fs).

| **Population** | **n** | **S** | **H** | **Hd** | **π** | **D** | **Fs** |
| --- | --- | --- | --- | --- | --- | --- | --- |
| **AL1** | 10 | 0 | **1** (H1) | 0 | 0 | n/c | n/c |
| **AL2** | 2 | 0 | **1** (H1) | 0 | 0 | n/c | n/c |
| **AL3** | 2 | 0 | **1** (H1) | 0 | 0 | n/c | n/c |
| **AS1** | 2 | 0 | **1** (H1) | 0 | 0 | n/c | n/c |
| **AS2** | 10 | 0 | **1** (H1) | 0 | 0 | n/c | n/c |
| **AS3** | 10 | 0 | **1** (H1) | 0 | 0 | n/c | n/c |
| **AS4** | 4 | 0 | **1** (H1) | 0 | 0 | n/c | n/c |
| **AUS1** | 22 | 0 | **1** (H4) | 0 | 0 | n/c | n/c |
| **AV1** | 9 | 0 | **1** (H1) | 0 | 0 | n/c | n/c |
| **AV2** | 8 | 0 | **1** (H1) | 0 | 0 | n/c | n/c |
| **AV3** | 9 | 0 | **1** (H1) | 0 | 0 | n/c | n/c |
| **BCN1** | 4 | 0 | **1** (H6) | 0 | 0 | n/c | n/c |
| **BCN2** | 2 | 0 | **1** (H1) | 0 | 0 | n/c | n/c |
| **BOS10** | 2 | 1 | **2** (H75. H76) | 1 | 0.00106 | n/c | 0 |
| **BOS2** | 3 | 0 | **1** (H79) | 0 | 0 | n/c | n/c |
| **BOS3** | 3 | 1 | **2** (H72. H78) | 0.667 | 0.0007 | n/c | 0.201 |
| **BOS4** | 3 | 1 | **2** (H71. H72) | 0.667 | 0.0007 | n/c | 0.201 |
| **BOS5** | 3 | 0 | **1** (H81) | 0 | 0 | n/c | n/c |
| **BOS6** | 2 | 3 | **2** (H73. H77) | 1 | 0.00317 | n/c | 1.099 |
| **BOS7** | 4 | 0 | **1** (H81) | 0 | 0 | n/c | n/c |
| **BOS8** | 2 | 0 | **1** (H81) | 0 | 0 | n/c | n/c |
| **BOS9** | 2 | 0 | **1** (H82) | 0 | 0 | n/c | n/c |
| **BU22** | 9 | 0 | **1** (H1) | 0 | 0 | n/c | n/c |
| **BU34** | 6 | 0 | **1** (H6) | 0 | 0 | n/c | n/c |
| **BU4** | 10 | 1 | **2** (H1. H16) | 0.356 | 0.00038 | 0.01499 | 0.417 |
| **BU53** | 7 | 1 | **2** (H1. H10) | 0.476 | 0.0005 | 0.55902 | 0.773 |
| **BU58** | 10 | 0 | **1** (H1) | 0 | 0 | n/c | n/c |
| **BU64** | 9 | 0 | **1** (H1) | 0 | 0 | n/c | n/c |
| **BU7** | 3 | 0 | **1** (H1) | 0 | 0 | n/c | n/c |
| **BU82** | 2 | 0 | **1** (H1) | 0 | 0 | n/c | n/c |
| **BU83** | 4 | 0 | **1** (H1) | 0 | 0 | n/c | n/c |
| **BU84** | 5 | 0 | **1** (H1) | 0 | 0 | n/c | n/c |
| **BU85** | 3 | 0 | **1** (H1) | 0 | 0 | n/c | n/c |
| **BU86** | 5 | 0 | **1** (H1) | 0 | 0 | n/c | n/c |
| **BU98** | 10 | 1 | **2** (H1. H13) | 0.2 | 0.00021 | -1.11173 | -0.339 |
| **BU99** | 10 | 3 | **2** (H1. H9) | 0.2 | 0.00063 | -1.56222 | 1.225 |
| **CAS1** | 10 | 0 | **1** (H1) | 0 | 0 | n/c | n/c |
| **CAS2** | 10 | 1 | **2** (H1. H6) | 0.556 | 0.0059 | 1.46364 | 1.096 |
| **CAS3** | 3 | 0 | **1** (H1) | 0 | 0 | n/c | n/c |
| **CAS4** | 3 | 0 | **1** (H1) | 0 | 0 | n/c | n/c |
| **COR1** | 3 | 0 | **1** (H1) | 0 | 0 | n/c | n/c |
| **CR2** | 4 | 1 | **2** (H1. H6) | 0.667 | 0.0007 | 1.63299 | 0.54 |
| **CRO1** | 4 | 2 | **2** (H28. H29) | 0.5 | 0.00106 | -0.7099 | 1.099 |
| **CRO10** | 4 | 0 | **1** (H59) | 0 | 0 | n/c | n/c |
| **CRO11** | 8 | 5 | **4** (H80. H83. H86. H87) | 0.75 | 0.002 | -0.08352 | 0.044 |
| **CRO12** | 3 | 0 | **1** (H78) | 0 | 0 | n/c | n/c |
| **CRO13** | 4 | 15 | **2** (H61. H70) | 0.5 | 0.00793 | -0.84729 | 4.944 |
| **CRO14** | 4 | 2 | **2** (H83. H90) | 0.5 | 0.00106 | -0.7099 | 1.099 |
| **CRO17** | 2 | 2 | **2** (H83. H92) | 1 | 0.00211 | n/c | 0.693 |
| **CRO18** | 2 | 1 | **2** (H83. H91) | 1 | 0.00106 | n/c | 0 |
| **CRO19** | 2 | 1 | **2** (H72. H80) | 1 | 0.00106 | n/c | 0 |
| **CRO2** | 7 | 5 | **4** (H48. H53. H54. H55) | 0.714 | 0.00151 | -1.48614 | -0.78 |
| **CRO20** | 3 | 6 | **3** (H74. H75. H88) | 1 | 0.00423 | n/c | 0.134 |
| **CRO21** | 4 | 5 | **3** (H56. H57. H60) | 0.833 | 0.003 | 0.37186 | 0.646 |
| **CRO22** | 3 | 13 | **2** (H53. H72) | 0.667 | 0.00916 | n/c | 3.923 |
| **CRO24** | 7 | 1 | **2** (H72. H79) | 0.571 | 0.0006 | 1.34164 | 0.856 |
| **CRO25** | 2 | 0 | **1** (H65) | 0 | 0 | n/c | n/c |
| **CRO3** | 2 | 2 | **2** (H58. H59) | 1 | 0.00211 | n/c | 0.693 |
| **CRO4** | 4 | 1 | **2** (H83. H84) | 0.5 | 0.00053 | -0.61237 | 0.172 |
| **CRO5** | 3 | 2 | **2** (H85. H89) | 0.667 | 0.00141 | n/c | 1.061 |
| **CRO6** | 2 | 1 | **2** (H83. H91) | 1 | 0.00106 | n/c | 0 |
| **CRO7** | 3 | 3 | **2** (H72. H90) | 0.667 | 0.00211 | n/c | 1.609 |
| **CRO8** | 5 | 13 | **2** (H66. H72) | 0.4 | 0.0055 | -1.21039 | 4.937 |
| **CRO9** | 7 | 38 | **4** (H23. H26. H27. H53) | 0.857 | 0.01903 | 0.92223 | 5.15 |
| **CU1** | 2 | 1 | **2** (H1. H6) | 1 | 0.00106 | n/c | 0 |
| **CU2** | 3 | 0 | **1** (H6) | 0 | 0 | n/c | n/c |
| **CU3** | 2 | 0 | **1** (H6) | 0 | 0 | n/c | n/c |
| **CU4** | 3 | 1 | **2** (H1. H6) | 0.667 | 0.0007 | n/c | 0.201 |
| **CU5** | 4 | 0 | **1** (H6) | 0 | 0 | n/c | n/c |
| **CU6** | 3 | 0 | **1** (H6) | 0 | 0 | n/c | n/c |
| **CU7** | 10 | 1 | **2** (H6. H8) | 0.2 | 0.00021 | -1.11173 | -0.339 |
| **CU8** | 10 | 0 | **1** (H6) | 0 | 0 | n/c | n/c |
| **CU9** | 10 | 2 | **3** (H1. H6. H20) | 0.6 | 0.00077 | 0.1203 | -0.101 |
| **FRA1** | 14 | 1 | **2** (H43. H49) | 0.495 | 0.00052 | 1.21219 | 1.139 |
| **FRA10** | 11 | 1 | **2** (H35. H36) | 0.327 | 0.00035 | -0.10001 | 0.356 |
| **FRA11** | 8 | 0 | **1** (H34) | 0 | 0 | n/c | n/c |
| **FRA13** | 13 | 2 | **3** (H34. H39. H40) | 0.5 | 0.00089 | 0.87897 | 0.436 |
| **FRA14** | 14 | 0 | **1** (H35) | 0 | 0 | n/c | n/c |
| **FRA15** | 8 | 38 | **4** (H6. H42. H43. H52) | 0.786 | 0.01053 | -1.71005 | 4.188 |
| **FRA16** | 9 | 0 | **1** (H34) | 0 | 0 | n/c | n/c |
| **FRA17** | 3 | 0 | **1** (H35) | 0 | 0 | n/c | n/c |
| **FRA18** | 7 | 0 | **1** (H34) | 0 | 0 | n/c | n/c |
| **FRA2** | 4 | 53 | **2** (H6. H33) | 0.5 | 0.02801 | -0.86786 | 8.256 |
| **FRA4** | 7 | 1 | **2** (H31. H32) | 0.476 | 0.0005 | 0.55902 | 0.589 |
| **FRA5** | 8 | 0 | **1** (H35) | 0 | 0 | n/c | n/c |
| **FRA6** | 9 | 1 | **2** (H34. H35) | 0.556 | 0.0059 | 1.40117 | 1.015 |
| **FRA7** | 8 | 0 | **1** (H35) | 0 | 0 | n/c | n/c |
| **FRA8** | 14 | 0 | **1** (H35) | 0 | 0 | n/c | n/c |
| **FRA9** | 11 | 0 | **1** (H35) | 0 | 0 | n/c | n/c |
| **GB1** | 3 | 0 | **1** (H34) | 0 | 0 | n/c | n/c |
| **GIR1** | 3 | 2 | **2** (H6. H10) | 0.667 | 0.00141 | n/c | 1.061 |
| **GIR10** | 21 | 2 | **2** (H41. H43) | 0.381 | 0.00081 | 0.85355 | 2.301 |
| **GIR11** | 3 | 0 | **1** (H1) | 0 | 0 | n/c | n/c |
| **GIR12** | 3 | 0 | **1** (H1) | 0 | 0 | n/c | n/c |
| **GIR2** | 11 | 0 | **1** (H1) | 0 | 0 | n/c | n/c |
| **GIR3** | 12 | 1 | **2** (H1. H6) | 0.485 | 0.00051 | 1.06589 | 1.003 |
| **GIR4** | 11 | 1 | **2** (H1. H6) | 0.182 | 0.00019 | -1.1285 | -0.41 |
| **GIR6** | 11 | 1 | **2** (H1. H6) | 0.509 | 0.00054 | 1.1856 | 1.023 |
| **GIR7** | 10 | 0 | **1** (H1) | 0 | 0 | n/c | n/c |
| **GIR8** | 10 | 0 | **1** (H1) | 0 | 0 | n/c | n/c |
| **GIR9** | 10 | 0 | **1** (H1) | 0 | 0 | n/c | n/c |
| **GRA1** | 10 | 0 | **1** (H1) | 0 | 0 | n/c | n/c |
| **GRA10** | 3 | 0 | **1** (H1) | 0 | 0 | n/c | n/c |
| **GRA11** | 3 | 0 | **1** (H1) | 0 | 0 | n/c | n/c |
| **GRA12** | 3 | 0 | **1** (H1) | 0 | 0 | n/c | n/c |
| **GRA13** | 3 | 0 | **1** (H1) | 0 | 0 | n/c | n/c |
| **GRA14** | 2 | 0 | **1** (H1) | 0 | 0 | n/c | n/c |
| **GRA15** | 3 | 0 | **1** (H1) | 0 | 0 | n/c | n/c |
| **GRA16** | 3 | 0 | **1** (H1) | 0 | 0 | n/c | n/c |
| **GRA17** | 3 | 0 | **1** (H1) | 0 | 0 | n/c | n/c |
| **GRA2** | 6 | 1 | **2** (H1. H11) | 0.333 | 0.00035 | -0.93302 | -0.003 |
| **GRA3** | 6 | 0 | **1** (H1) | 0 | 0 | n/c | n/c |
| **GRA4** | 7 | 1 | **2** (H1. H6) | 0.286 | 0.0003 | -1.00623 | -0.095 |
| **GRA5** | 5 | 0 | **1** (H1) | 0 | 0 | n/c | n/c |
| **GRA6** | 5 | 0 | **1** (H1) | 0 | 0 | n/c | n/c |
| **GRA7** | 6 | 0 | **1** (H1) | 0 | 0 | n/c | n/c |
| **GRA8** | 5 | 0 | **1** (H1) | 0 | 0 | n/c | n/c |
| **GRA9** | 3 | 3 | **3** (H1. H2. H3) | 1 | 0.00211 | n/c | -0.693 |
| **GU1** | 5 | 1 | **2** (H1. H6) | 0.6 | 0.00063 | 1.22474 | 0.626 |
| **GU2** | 10 | 1 | **2** (H1. H6) | 0.556 | 0.00059 | 1.46364 | 1.096 |
| **HU1** | 5 | 0 | **1** (H6) | 0 | 0 | n/c | n/c |
| **HU2** | 2 | 0 | **1** (H1) | 0 | 0 | n/c | n/c |
| **HU3** | 10 | 0 | **1** (H1) | 0 | 0 | n/c | n/c |
| **HU4** | 5 | 1 | **2** (H1. H6) | 0.6 | 0.00063 | 1.22474 | 0.626 |
| **HU5** | 3 | 0 | **1** (H6) | 0 | 0 | n/c | n/c |
| **HU6** | 2 | 0 | **1** (H1) | 0 | 0 | n/c | n/c |
| **HU7** | 3 | 1 | **2** (H1. H6) | 0.667 | 0.0007 | n/c | 0.201 |
| **HU8** | 3 | 0 | **1** (H6) | 0 | 0 | n/c | n/c |
| **IRE1** | 2 | 0 | **1** (H34) | 0 | 0 | n/c | n/c |
| **IRE2** | 4 | 1 | **2** (H34. H38) | 0.5 | 0.00053 | 0.61237 | 0.172 |
| **IRE3** | 5 | 0 | **1** (H34) | 0 | 0 | n/c | n/c |
| **IT1** | 10 | 1 | **2** (H1. H6) | 0.467 | 0.00049 | 0.8198 | 0.818 |
| **IT10** | 2 | 0 | **1** (H64) | 0 | 0 | n/c | n/c |
| **IT11** | 3 | 0 | **1** (H64) | 0 | 0 | n/c | n/c |
| **IT12** | 3 | 0 | **1** (H64) | 0 | 0 | n/c | n/c |
| **IT13** | 12 | 0 | **1** (H1) | 0 | 0 | n/c | n/c |
| **IT14** | 13 | 0 | **1** (H19) | 0 | 0 | n/c | n/c |
| **IT15** | 10 | 0 | **1** (H37) | 0 | 0 | n/c | n/c |
| **IT16** | 2 | 0 | **1** (H64) | 0 | 0 | n/c | n/c |
| **IT17** | 2 | 0 | **1** (H64) | 0 | 0 | n/c | n/c |
| **IT18** | 14 | 2 | **3** (H43. H44. H50) | 0.604 | 0.00071 | 0.17874 | 0.055 |
| **IT19** | 12 | 36 | **3** (H24. H43. H45) | 0.545 | 0.01826 | 2.03628 | 13.041 |
| **IT2** | 4 | 0 | **1** (H18) | 0 | 0 | n/c | n/c |
| **IT20** | 5 | 0 | **1** (H24) | 0 | 0 | n/c | n/c |
| **IT21** | 5 | 0 | **1** (H24) | 0 | 0 | n/c | n/c |
| **IT22** | 5 | 0 | **1** (H25) | 0 | 0 | n/c | n/c |
| **IT23** | 5 | 0 | **1** (H51) | 0 | 0 | n/c | n/c |
| **IT24** | 5 | 0 | **1** (H51) | 0 | 0 | n/c | n/c |
| **IT25** | 5 | 0 | **1** (H51) | 0 | 0 | n/c | n/c |
| **IT26** | 2 | 0 | **1** (H46) | 0 | 0 | n/c | n/c |
| **IT27** | 3 | 0 | **1** (H47) | 0 | 0 | n/c | n/c |
| **IT28** | 10 | 0 | **1** (H14) | 0 | 0 | n/c | n/c |
| **IT29** | 4 | 3 | **2** (H4. H5) | 0.667 | 0.00211 | 2.01187 | 2.197 |
| **IT3** | 2 | 0 | **1** (H18) | 0 | 0 | n/c | n/c |
| **IT30** | 5 | 0 | **1** (H30) | 0 | 0 | n/c | n/c |
| **IT4** | 5 | 2 | **2** (H21. H22) | 0.6 | 0.00127 | 1.45884 | 1.688 |
| **IT5** | 3 | 0 | **1** (H23) | 0 | 0 | n/c | n/c |
| **IT6** | 2 | 7 | **2** (H64. H69) | 1 | 0.0074 | n/c | 1.946 |
| **IT7** | 6 | 0 | **1** (H62) | 0 | 0 | n/c | n/c |
| **IT8** | 4 | 1 | **2** (H67. H68) | 0.5 | 0.00053 | -0.61237 | 0.172 |
| **IT9** | 3 | 0 | **1** (H62) | 0 | 0 | n/c | n/c |
| **JA1** | 2 | 0 | **1** (H1) | 0 | 0 | n/c | n/c |
| **JA10** | 3 | 0 | **1** (H1) | 0 | 0 | n/c | n/c |
| **JA11** | 3 | 1 | **2** (H1. H6) | 0.667 | 0.0007 | n/c | 0.201 |
| **JA12** | 4 | 1 | **2** (H1. H6) | 0.5 | 0.00053 | -0.61237 | 0.172 |
| **JA13** | 3 | 0 | **1** (H1) | 0 | 0 | n/c | n/c |
| **JA14** | 3 | 0 | **1** (H1) | 0 | 0 | n/c | n/c |
| **JA15** | 3 | 0 | **1** (H1) | 0 | 0 | n/c | n/c |
| **JA3** | 5 | 0 | **1** (H1) | 0 | 0 | n/c | n/c |
| **JA4** | 5 | 0 | **1** (H6) | 0 | 0 | n/c | n/c |
| **JA5** | 5 | 1 | **2** (H1. H6) | 0.6 | 0.00063 | 1.22474 | 0.626 |
| **JA6** | 5 | 0 | **1** (H1) | 0 | 0 | n/c | n/c |
| **JA7** | 3 | 1 | **2** (H1. H15) | 0.667 | 0.0007 | n/c | 0.201 |
| **JA8** | 3 | 0 | **1** (H1) | 0 | 0 | n/c | n/c |
| **JA9** | 3 | 0 | **1** (H1) | 0 | 0 | n/c | n/c |
| **LE1** | 10 | 0 | **1** (H1) | 0 | 0 | n/c | n/c |
| **LE3** | 11 | 0 | **1** (H1) | 0 | 0 | n/c | n/c |
| **LER1** | 10 | 0 | **1** (H6) | 0 | 0 | n/c | n/c |
| **LER2** | 10 | 0 | **1** (H6) | 0 | 0 | n/c | n/c |
| **LU1** | 10 | 0 | **1** (H1) | 0 | 0 | n/c | n/c |
| **LU2** | 10 | 0 | **1** (H1) | 0 | 0 | n/c | n/c |
| **MA1** | 5 | 0 | **1** (H1) | 0 | 0 | n/c | n/c |
| **MA2** | 5 | 0 | **1** (H1) | 0 | 0 | n/c | n/c |
| **MA3** | 6 | 0 | **1** (H1) | 0 | 0 | n/c | n/c |
| **MA4** | 3 | 1 | **2** (H1. H2) | 0.667 | 0.0007 | n/c | 0.201 |
| **MA5** | 3 | 0 | **1** (H1) | 0 | 0 | n/c | n/c |
| **MA6** | 3 | 0 | **1** (H1) | 0 | 0 | n/c | n/c |
| **MA7** | 3 | 0 | **1** (H1) | 0 | 0 | n/c | n/c |
| **MAD1** | 3 | 0 | **1** (H1) | 0 | 0 | n/c | n/c |
| **MON1** | 10 | 1 | **2** (H72. H79) | 0.2 | 0.00021 | -1.11173 | n/c |
| **NA2** | 3 | 0 | **1** (H1) | 0 | 0 | n/c | n/c |
| **NA3** | 3 | 1 | **2** (H1. H6) | 0.667 | 0.0007 | N/C | 0.201 |
| **NA4** | 4 | 0 | **1** (H1) | 0 | 0 | n/c | n/c |
| **NA5** | 4 | 0 | **1** (H1) | 0 | 0 | n/c | n/c |
| **NA7** | 2 | 0 | **1** (H1) | 0 | 0 | n/c | n/c |
| **NA8** | 10 | 0 | **1** (H1) | 0 | 0 | n/c | n/c |
| **PA1** | 4 | 1 | **2** (H1. H6) | 0.667 | 0.0007 | 1.63299 | 0.54 |
| **RI1** | 3 | 0 | **1** (H1) | 0 | 0 | n/c | n/c |
| **RI2** | 3 | 0 | **1** (H1) | 0 | 0 | n/c | n/c |
| **RI3** | 3 | 0 | **1** (H1) | 0 | 0 | n/c | n/c |
| **RI4** | 3 | 0 | **1** (H1) | 0 | 0 | n/c | n/c |
| **SLO1** | 5 | 1 | **2** (H63. H64) | 0.4 | 0.00042 | -0.8165 | 0.09 |
| **SO1** | 10 | 1 | **2** (H1. H6) | 0.356 | 0.00038 | 0.01499 | 0.417 |
| **SO15** | 9 | 0 | **1** (H1) | 0 | 0 | n/c | n/c |
| **SO2** | 9 | 1 | **2** (H1. H6) | 0.5 | 0.00053 | 0.98627 | 0.849 |
| **SO3** | 2 | 1 | **2** (H1. H6) | 1 | 0.00106 | n/c | 0 |
| **SO8** | 9 | 1 | **2** (H1. H6) | 0.389 | 0.00041 | 0.15647 | 0.477 |
| **TAR1** | 4 | 0 | **1** (H6) | 0 | 0 | n/c | n/c |
| **TAR2** | 3 | 0 | **1** (H6) | 0 | 0 | n/c | n/c |
| **TAR3** | 10 | 1 | **2** (H1. H6) | 0.467 | 0.00049 | 0.8198 | 0.818 |
| **TE1** | 2 | 1 | **2** (H1. H12) | 1 | 0.00106 | n/c | 0 |
| **TE10** | 4 | 0 | **1** (H1) | 0 | 0 | n/c | n/c |
| **TE11** | 5 | 1 | **2** (H1. H6) | 0.4 | 0.00042 | -0.8165 | 0.09 |
| **TE12** | 5 | 0 | **1** (H1) | 0 | 0 | n/c | n/c |
| **TE13** | 5 | 0 | **1** (H1) | 0 | 0 | n/c | n/c |
| **TE14** | 5 | 0 | **1** (H6) | 0 | 0 | n/c | n/c |
| **TE15** | 7 | 1 | **2** (H1. H6) | 0.286 | 0.0003 | -1.00623 | -0.095 |
| **TE16** | 3 | 0 | **1** (H1) | 0 | 0 | n/c | n/c |
| **TE17** | 3 | 0 | **1** (H6) | 0 | 0 | n/c | n/c |
| **TE18** | 3 | 0 | **1** (H1) | 0 | 0 | n/c | n/c |
| **TE2** | 10 | 0 | **1** (H1) | 0 | 0 | n/c | n/c |
| **TE3** | 10 | 1 | **2** (H1. H6) | 0.467 | 0.00049 | 0.8198 | 0.818 |
| **TE5** | 10 | 2 | **3** (H1. H6. H16) | 0.644 | 0.00077 | 0.1203 | -0.101 |
| **TE6** | 4 | 1 | **2** (H1. H6) | 0.5 | 0.00053 | -0.61237 | 0.172 |
| **TE7** | 3 | 2 | **3** (H1. H6. H17) | 1 | 0.00141 | n/c | -1.216 |
| **TE8** | 2 | 0 | **1** (H1) | 0 | 0 | n/c | n/c |
| **TE9** | 4 | 0 | **1** (H1) | 0 | 0 | n/c | n/c |
| **VA1** | 10 | 1 | **2** (H1. H6) | 0.356 | 0.00038 | 0.01499 | 0.417 |
| **VA2** | 3 | 0 | **1** (H1) | 0 | 0 | n/c | n/c |
| **VA3** | 3 | 0 | **1** (H1) | 0 | 0 | n/c | n/c |
| **VALL1** | 6 | 0 | **1** (H1) | 0 | 0 | n/c | n/c |
| **VALL2** | 2 | 0 | **1** (H1) | 0 | 0 | n/c | n/c |
| **VIZ1** | 11 | 0 | **1** (H1) | 0 | 0 | n/c | n/c |
| **VIZ2** | 9 | 0 | **1** (H1) | 0 | 0 | n/c | n/c |
| **ZA1** | 10 | 1 | **2** (H1. H6) | 0.356 | 0.00038 | 0.01499 | 0.417 |
| **ZA2** | 3 | 1 | **2** (H1. H6) | 0.667 | 0.0007 | n/c | 0.201 |
| **ZA3** | 5 | 1 | **2** (H1. H6) | 0.4 | 0.00042 | -0.8165 | 0.09 |
| **ZA4** | 5 | 2 | **3** (H1. H6. H7) | 0.8 | 0.00106 | 0.24314 | -0.475 |
| **ZA5** | 3 | 0 | **1** (H1) | 0 | 0 | n/c | n/c |
