# Supplementary material for: On the conservation of white-clawed crayfish in the Iberian Peninsula: Unraveling its genetic diversity and structure, and origin
Source: PLoS One. 2023 Oct 13;18(10):e0292679. doi: 10.1371/journal.pone.0292679 (PMC10575519; doi:10.1371/journal.pone.0292679)
Supplement: S4 Table — Genetic diversity indices of the populations from dataset 2 (sequences from the Iberian and Italian peninsulas) based on 2449 bp of the concatenated mitochondrial 16S rRNA and cytochrome oxidase subunit I regions: sample size (n), the number of polymorphic sites (S), the number of haplotypes (H), the haplotype diversity (Hd), the nucleotide diversity (π), the Tajima’s D (D), and Fu’s Fs (Fs). (DOCX) [file pone.0292679.s010.docx]

**S4 Table. Dataset 2 genetic diversity indices** Genetic diversity indices of the populations from dataset 2 (sequences from the Iberian and Italian peninsulas) based on 2449 bp of the concatenated mitochondrial 16S rRNA and cytochrome oxidase subunit I regions: sample size (n), the number of polymorphic sites (S), the number of haplotypes (H), the haplotype diversity (Hd), the nucleotide diversity (π), the Tajima’s D (D), and Fu’s Fs (Fs).

| **Population** | **n** | **S** | **H** | **Hd** | **π** | **D** | **Fs** |
| --- | --- | --- | --- | --- | --- | --- | --- |
| **AL1** | 10 | 0 | **1** (H24) | 0 | 0 | n/c | n/c |
| **AS1** | 2 | 0 | **1** (H30) | 0 | 0 | n/c | n/c |
| **AS2** | 10 | 0 | **1** (H30) | 0 | 0 | n/c | n/c |
| **AS3** | 10 | 0 | **1** (H30) | 0 | 0 | n/c | n/c |
| **AV1** | 9 | 0 | **1** (H1) | 0 | 0 | n/c | n/c |
| **AV2** | 8 | 0 | **1** (H1) | 0 | 0 | n/c | n/c |
| **AV3** | 9 | 0 | **1** (H1) | 0 | 0 | n/c | n/c |
| **BU22** | 9 | 3 | **4** (H1, H9, H13, H15) | 0.833 | 0.00054 | 0.79438 | -0.45 |
| **BU34** | 6 | 0 | **1** (H16) | 0 | 0 | n/c | n/c |
| **BU4** | 10 | 1 | **2** (H30, H32) | 0.356 | 0.00014 | 0.01499 | 0.417 |
| **BU53** | 7 | 4 | **4** (H24, H26, H27, H28) | 0.81 | 0.00062 | -0.3187 | -0.655 |
| **BU58** | 10 | 1 | **2** (H24, H30) | 0.533 | 0.00022 | 1.30268 | 1.029 |
| **BU64** | 9 | 1 | **2** (H1, H13) | 0.5 | 0.0002 | 0.98627 | 0.849 |
| **BU7** | 3 | 0 | **1** (H24) | 0 | 0 | n/c | n/c |
| **BU82** | 2 | 1 | **2** (H29, H30) | 1 | 0.00041 | n/c | 0 |
| **BU83** | 4 | 0 | **1** (H30) | 0 | 0 | n/c | n/c |
| **BU84** | 5 | 0 | **1** (H30) | 0 | 0 | n/c | n/c |
| **BU85** | 3 | 0 | **1** (H30) | 0 | 0 | n/c | n/c |
| **BU86** | 5 | 2 | **3** (H30, H34, H35) | 0.7 | 0.00041 | 0.24314 | -0.475 |
| **BU98** | 10 | 1 | **2** (H30, H31) | 0.2 | 0.00008 | -1.11173 | -0.339 |
| **BU99** | 10 | 6 | **2** (H21, H30) | 0.2 | 0.00049 | -1.79631 | 2.607 |
| **CAS1** | 10 | 0 | **1** (H1) | 0 | 0 | n/c | n/c |
| **CAS2** | 10 | 2 | **3** (H1, H5, H16) | 0.644 | 0.00044 | 1.74286 | 0.643 |
| **CR2** | 4 | 1 | **2** (H1, H16) | 0.667 | 0.00027 | 1.63299 | 0.54 |
| **CU1** | 2 | 1 | **2** (H1, H16) | 1 | 0.00041 | n/c | 0 |
| **CU2** | 3 | 0 | **1** (H16) | 0 | 0 | n/c | n/c |
| **CU3** | 2 | 1 | **2** (H16, H19) | 1 | 0.00041 | n/c | 0 |
| **CU4** | 3 | 3 | **3** (H1, H16, H24) | 1 | 0.00081 | n/c | -0.693 |
| **CU5** | 4 | 0 | **1** (H16) | 0 | 0 | n/c | n/c |
| **CU6** | 3 | 0 | **1** (H16) | 0 | 0 | n/c | n/c |
| **CU7** | 10 | 1 | **2** (H16, H17) | 0.2 | 0.00008 | -1.11173 | -0.339 |
| **CU8** | 10 | 0 | **1** (H16) | 0 | 0 | n/c | n/c |
| **CU9** | 10 | 2 | **3** (H1, H4, H16) | 0.6 | 0.0003 | 0.1203 | -0.101 |
| **GIR1** | 3 | 3 | **2** (H14, H16) | 0.667 | 0.00081 | n/c | 1.609 |
| **GIR2** | 11 | 1 | **2** (H1, H2) | 0.327 | 0.00013 | -0.10001 | 0.356 |
| **GIR3** | 12 | 1 | **2** (H1, H16) | 0.485 | 0.0002 | 1.06589 | 1.003 |
| **GIR4** | 11 | 3 | **3** (H1, H10, H20) | 0.345 | 0.00022 | -1.59996 | -0.537 |
| **GIR6** | 11 | 2 | **3** (H1, H2, H16) | 0.564 | 0.00028 | 0.03616 | -0.113 |
| **GIR7** | 10 | 1 | **2** (H1, H6) | 0.2 | 0.00008 | -1.11173 | -0.339 |
| **GRA1** | 10 | 0 | **1** (H1) | 0 | 0 | n/c | n/c |
| **GRA2** | 5 | 1 | **2** (H1, H43) | 0.4 | 0.00016 | -0.8165 | 0.09 |
| **GRA3** | 6 | 0 | **1** (H1) | 0 | 0 | n/c | n/c |
| **GRA4** | 7 | 1 | **2** (H1, H16) | 0.286 | 0.00012 | -1.00623 | -0.095 |
| **GRA5** | 5 | 1 | **2** (H1, H44) | 0.6 | 0.00024 | 1.22474 | 0.626 |
| **GRA6** | 5 | 0 | **1** (H1) | 0 | 0 | n/c | n/c |
| **GRA7** | 6 | 0 | **1** (H1) | 0 | 0 | n/c | n/c |
| **GRA8** | 5 | 0 | **1** (H1) | 0 | 0 | n/c | n/c |
| **GU1** | 5 | 1 | **2** (H1, H16) | 0.6 | 0.00024 | 1.22474 | 0.626 |
| **GU2** | 10 | 1 | **2** (H1, H16) | 0.556 | 0.00023 | 1.46364 | 1.096 |
| **HU1** | 5 | 0 | **1** (H16) | 0 | 0 | n/c | n/c |
| **HU2** | 2 | 0 | **1** (H1) | 0 | 0 | n/c | n/c |
| **HU3** | 10 | 7 | **4** (H1, H3, H12, H24) | 0.711 | 0.00708 | -0.92618 | 0.517 |
| **HU4** | 5 | 1 | **2** (H1, H16) | 0.6 | 0.00024 | 1.22474 | 0.626 |
| **HU5** | 3 | 0 | **1** (H16) | 0 | 0 | n/c | n/c |
| **HU6** | 2 | 3 | **2** (H1, H36) | 1 | 0.00122 | n/c | 1.099 |
| **IT1** | 10 | 3 | **3** (H1, H37, H38) | 0.733 | 0.00057 | 1.15198 | 1.16 |
| **IT2** | 4 | 0 | **1** (H39) | 0 | 0 | n/c | n/c |
| **IT3** | 2 | 0 | **1** (H39) | 0 | 0 | n/c | n/c |
| **IT4** | 5 | 7 | **3** (H40, H41, H42) | 0.8 | 0.00138 | 0.08298 | 1.569 |
| **JA1** | 2 | 0 | **1** (H1) | 0 | 0 | n/c | n/c |
| **JA3** | 5 | 0 | **1** (H1) | 0 | 0 | n/c | n/c |
| **JA4** | 5 | 0 | **1** (H16) | 0 | 0 | n/c | n/c |
| **JA5** | 5 | 1 | **2** (H1, H16) | 0.6 | 0.00024 | 1.22474 | 0.626 |
| **JA6** | 5 | 0 | **1** (H1) | 0 | 0 | n/c | n/c |
| **LE1** | 10 | 3 | **3** (H1, H24, H30) | 0.6 | 0.00035 | -0.65748 | 0.206 |
| **LE3** | 11 | 1 | **2** (H1, H13) | 0.436 | 0.00018 | 0.67135 | 0.779 |
| **LER1** | 10 | 2 | **3** (H16, H22, H23) | 0.622 | 0.00029 | 0.01889 | -0.156 |
| **LU1** | 10 | 1 | **2** (H1, H8) | 0.2 | 0.00008 | -1.11173 | -0.339 |
| **LU2** | 10 | 0 | **1** (H1) | 0 | 0 | n/c | n/c |
| **MA1** | 5 | 0 | **1** (H1) | 0 | 0 | n/c | n/c |
| **MA2** | 5 | 0 | **1** (H1) | 0 | 0 | n/c | n/c |
| **MA3** | 6 | 0 | **1** (H1) | 0 | 0 | n/c | n/c |
| **MAD1** | 3 | 0 | **1** (H1) | 0 | 0 | n/c | n/c |
| **NA2** | 3 | 1 | **2** (H1) | 0.667 | 0.00027 | n/c | 0.201 |
| **NA3** | 3 | 3 | **2** (H24, H30) | 0.667 | 0.00081 | n/c | 1.609 |
| **NA4** | 4 | 0 | **1** (H24) | 0 | 0 | n/c | n/c |
| **NA5** | 4 | 1 | **2** (H24, H30) | 0.5 | 0.0002 | -0.61237 | 0.172 |
| **NA7** | 2 | 1 | **2** (H24, H30) | 1 | 0.00041 | n/c | 0 |
| **NA8** | 10 | 3 | **2** (H11, H24) | 0.2 | 0.00024 | -1.56222 | 1.225 |
| **PA1** | 4 | 4 | **2** (H16, H30) | 0.667 | 0.00108 | 2.08033 | 2.719 |
| **SO1** | 10 | 1 | **2** (H1, H16) | 0.356 | 0.00014 | 0.01499 | 0.417 |
| **SO15** | 9 | 0 | **1** (H24) | 0 | 0 | n/c | n/c |
| **SO2** | 9 | 1 | **2** (H1, H16) | 0.5 | 0.0002 | 0.98627 | 0.849 |
| **SO3** | 2 | 1 | **2** (H1, H16) | 1 | 0.00041 | n/c | 0 |
| **SO8** | 9 | 2 | **3** (H1, H13, H16) | 0.556 | 0.00025 | -0.58325 | -0.532 |
| **TE1** | 2 | 1 | **2** (H1, H7) | 1 | 0.00041 | n/c | 0 |
| **TE10** | 4 | 0 | **1** (H1) | 0 | 0 | n/c | n/c |
| **TE11** | 5 | 1 | **2** (H1, H16) | 0.4 | 0.00016 | -0.8165 | 0.09 |
| **TE12** | 5 | 1 | **2** (H1, H46) | 0.4 | 0.00016 | -0.8165 | 0.09 |
| **TE13** | 5 | 0 | **1** (H1) | 0 | 0 | n/c | n/c |
| **TE14** | 5 | 0 | **1** (H16) | 0 | 0 | n/c | n/c |
| **TE15** | 4 | 1 | **2** (H1, H16) | 0.5 | 0.0002 | -0.61237 | 0.172 |
| **TE2** | 10 | 0 | **1** (H1) | 0 | 0 | n/c | n/c |
| **TE3** | 10 | 1 | **2** (H1, H16) | 0.467 | 0.00019 | 0.8198 | 0.818 |
| **TE5** | 10 | 7 | **5** (H1, H8, H16, H18, H33) | 0.8 | 0.00077 | -1.00226 | -0.733 |
| **TE6** | 4 | 1 | **2** (H1, H16) | 0.5 | 0.0002 | -0.61237 | 0.172 |
| **TE7** | 3 | 2 | **3** (H1, H16, H45) | 1 | 0.00054 | n/c | -1.216 |
| **TE8** | 2 | 0 | **1** (H24) | 0 | 0 | n/c | n/c |
| **TE9** | 4 | 0 | **1** (H1) | 0 | 0 | n/c | n/c |
| **VA1** | 10 | 4 | **2** (H1, H25) | 0.356 | 0.00058 | 0.02248 | 3.025 |
| **VALL1** | 6 | 0 | **1** (H1) | 0 | 0 | n/c | n/c |
| **VALL2** | 2 | 0 | **1** (H1) | 0 | 0 | n/c | n/c |
| **ZA1** | 10 | 3 | **3** (H1, H16, H24) | 0.689 | 0.00052 | 0.77501 | 0.985 |
| **ZA2** | 3 | 5 | **3** (H24, H47, H48) | 1 | 0.00135 | n/c | -0.077 |
| **ZA3** | 5 | 1 | **2** (H1, H16) | 0.4 | 0.00016 | -0.8165 | 0.09 |
| **ZA4** | 5 | 2 | **3** (H1, H16, H49) | 0.8 | 0.00041 | 0.24314 | -0.475 |
