# Supplementary material for: On the conservation of white-clawed crayfish in the Iberian Peninsula: Unraveling its genetic diversity and structure, and origin
Source: PLoS One. 2023 Oct 13;18(10):e0292679. doi: 10.1371/journal.pone.0292679 (PMC10575519; doi:10.1371/journal.pone.0292679)
Supplement: S5 Table — Population groups obtained for the optimum genetic structure (K = 20) of the dataset 1 (sequences from the whole geographical distribution range, covering all lineages and clades previously defined to the WCC species complex) based on 948 bp of the concatenated mitochondrial 16S rRNA and cytochrome oxidase subunit I regions. (DOCX) [file pone.0292679.s011.docx]

**S5 Table**. **Genetic structure** **population groups for dataset 1.** Population groups obtained for the optimum genetic structure (K=20) of the dataset 1 (sequences from the whole geographical distribution range, covering all lineages and clades previously defined to the WCC species complex) based on 948 bp of the concatenated mitochondrial 16S rRNA and cytochrome oxidase subunit I regions.

| **Group** | **Populations** | **N indiv.** |
| --- | --- | --- |
| **G_d1_1** | **162** (IT2, IT3, IT4, AL1, AS1, AS2, AS3, AV1, AV2, AV3, BU4, BU7, BU22, BU34, BU53, BU58, BU64, BU82, BU83, BU84, BU85, BU86, BU98, BU99, CAS1, CAS2, CR2, CU1, CU2, CU3, CU4, CU5, CU6, CU7, CU8, CU9, GIR1, GIR2, GIR3, GIR4, GIR6, GIR7, GRA1, GRA2, GRA3, GRA4, GRA5, GRA6, GRA7, GRA8, GU1, GU2, HU1, HU2, HU3, HU4, HU5, HU6, IT1, JA1, JA3, JA4, JA5, JA6, LE1, LE3, LER1, LU1, LU2, MA1, MA2, MA3, MAD1, NA2, NA3, NA4, NA5, NA7, NA8, PA1, SO1, SO2, SO3, SO8, SO15, TE1, TE2, TE3, TE5, TE6, TE7, TE8, TE9, TE10, TE11, TE12, TE13, TE14, TE15, VA1, VALL1, VALL2, ZA1, ZA2, ZA3, ZA4, GIR11, GIR12, GIR8, GIR9, GRA10, GRA11, GRA12, GRA13, GRA14, GRA15, GRA16, GRA17, GRA9, HU7, HU8, IT13, IT14, IT28, IT29, JA10, JA11, JA12, JA13, JA14, JA15, JA7, JA8, JA9, LER2, MA4, MA5, MA6, MA7, RI1, RI2, RI3, RI4, TAR1, TAR2, TAR3, TE16, TE17, TE18, VA2, VA3, VIZ1, VIZ2, ZA5, AL2, AL3, AS4, BCN1, BCN2, CAS3, CAS4, COR1) | 906 |
| **G _d1_2** | **1** (IT30) | 5 |
| **G _d1_3** | **5** (CRO1, IT20, IT21, IT22, IT5) | 22 |
| **G _d1_4** | **8** (FRA10, FRA14, FRA17, FRA2, FRA4, FRA5, FRA6, FRA9) | 67 |
| **G _d1_5** | **3** (FRA7, FRA8, IT15) | 32 |
| **G _d1_6** | **3** (IT6, IT11, IT12) | 12 |
| **G _d1_7** | **6** (IT18, IT23, IT24, IT25, IT26, IT27) | 34 |
| **G _d1_8** | 1 (AUS1) | 22 |
| **G _d1_9** | **7** (CRO8, MON1, BOS3 BOS5, BOS6, BOS7, CRO12) | 32 |
| **G _d1_10** | **2** (IT8, IT10) | 6 |
| **G _d1_11** | **8** (FRA11, FRA13, FRA16, FRA18, GB1, IRE1, IRE2, IRE3) | 51 |
| **G _d1_12** | **2** (SLO1, CRO25) | 7 |
| **G _d1_13** | **2** (FRA15, GIR10) | 29 |
| **G _d1_14** | **2** (IT7, IT9) | 9 |
| **G _d1_15** | **1** (FRA1) | 14 |
| **G _d1_16** | **8** (CRO7, BOS2, BOS4, BOS8, BOS10, CRO11, CRO20, CRO24) | 31 |
| **G _d1_17** | **1** (CRO9) | 7 |
| **G _d1_18** | **6** (CRO2, CRO3, CRO10, CRO13, CRO21, CRO22) | 24 |
| **G _d1_19** | **7** (CRO4, CRO5, CRO6, CRO14, CRO17, CRO18, CRO19) | 19 |
| **G _d1_20** | **1** (IT19) | 12 |
