# Supplementary material for: On the conservation of white-clawed crayfish in the Iberian Peninsula: Unraveling its genetic diversity and structure, and origin
Source: PLoS One. 2023 Oct 13;18(10):e0292679. doi: 10.1371/journal.pone.0292679 (PMC10575519; doi:10.1371/journal.pone.0292679)
Supplement: S6 Table — Population groups obtained for the optimum genetic structure (K = 18) of the dataset 2 (sequences from the Iberian and Italian peninsulas) based on 2449 bp of the concatenated mitochondrial 16S rRNA and cytochrome oxidase subunit I regions. (DOCX) [file pone.0292679.s012.docx]

**S6 Table**. **Genetic structure** **population groups for dataset 2.** Population groups obtained for the optimum genetic structure (K=18) of the dataset 2 (sequences from the Iberian and Italian peninsulas) based on 2449 bp of the concatenated mitochondrial 16S rRNA and cytochrome oxidase subunit I regions.

| **Group** | **Populations** | **N indv.** |
| --- | --- | --- |
| **G _d2_1** | **62** (AV1, AV2, AV3, BU22, BU34, BU64, CAS1, CAS2, CR2, CU1, CU4, CU9, GIR3, GIR4, GIR6, GIR7, GRA1, GRA2, GRA3, GRA4, GRA5, GRA6, GRA7, GU1, GU2, HU2, HU3, HU4, IT1, JA1, GRA8, JA3, JA5, JA6, LE3, LU1, LU2, MA1, MA2, MA3, MAD1, PA1, SO1, SO2, SO3, SO8, TE1, TE2, TE3, TE5, TE6, TE7, TE10, TE11, TE12, TE13, TE15, VALL1, VALL2, ZA1, ZA3, ZA4) | 416 |
| **G _d2_2** | **1** (GIR2) | 11 |
| **G _d2_3** | **1** (IT4) | 5 |
| **G _d2_4** | **11** (AS1, AS2, AS3, BU4, BU82, BU83, BU84, BU85, BU86, BU98, BU99) | 71 |
| **G _d2_5** | **7** (CU2, CU3, CU5 CU6, CU7, CU8, JA4) | 38 |
| **G _d2_6** | **2** (NA3, ZA2) | 6 |
| **G _d2_7** | **2** (IT2, IT3) | 6 |
| **G _d2_8** | **1** (LE1) | 10 |
| **G _d2_9** | **2** (TE14, VA1) | 15 |
| **G _d2_10** | **1** (TE9) | 4 |
| **G _d2_11** | **2** (BU7, BU58) | 13 |
| **G _d2_12** | **7** (AL1, NA2, NA4, NA5, NA7, NA8, SO15) | 42 |
| **G _d2_13** | **1** (BU53) | 7 |
| **G _d2_14** | **1** (LER1) | 10 |
| **G _d2_15** | **2** (HU1, HU5) | 8 |
| **G _d2_16** | **1** (TE8) | 2 |
| **G _d2_17** | **1** (HU6) | 2 |
| **G _d2_18** | **1** (GIR1) | 3 |
